# Sequence-encoded tubular architectures in disordered spider silk proteins revealed by multiscale simulations and NMR

**DOI:** 10.1093/pnasnexus/pgaf378

**Published:** 2025-12-03

**Authors:** Christopher J Forman, David Onofrei, Dillan Stengel, Julian E Aldana, Christopher Paolini, Nathan C Gianneschi, Gregory P Holland

**Affiliations:** Department of Chemistry, Northwestern University, Evanston, IL 60208, USA; Department of Chemistry and Biochemistry, San Diego State University (SDSU), San Diego, CA 92182, USA; Department of Chemistry and Biochemistry, San Diego State University (SDSU), San Diego, CA 92182, USA; Department of Chemistry and Biochemistry, San Diego State University (SDSU), San Diego, CA 92182, USA; Department of Electrical and Computer Engineering, San Diego State University (SDSU), San Diego, CA 92182, USA; Department of Chemistry, Northwestern University, Evanston, IL 60208, USA; Department of Materials Science and Engineering, Northwestern University, Evanston, IL 60208, USA; Department of Biomedical Engineering, Northwestern University, Evanston, IL 60208, USA; Department of Chemistry and Biochemistry, San Diego State University (SDSU), San Diego, CA 92182, USA

**Keywords:** spider silk proteins, molecular dynamics, NMR, secondary structure, tertiary structure

## Abstract

Spider silk proteins (spidroins) are large, block-copolymer-like proteins that must remain soluble at high concentration in the spinning dope while being primed for rapid fiber formation. Understanding how these intrinsically disordered proteins organize in solution is key to explaining the transformation from soluble dope to solid fibers with exceptional strength and toughness. Here, we show that major ampullate (Ma) spidroins from the black widow spider form dynamic ensembles that include metastable tubular substructures. Multiscale molecular dynamics (MD) simulations reveal compact, anisotropic monomers with tubular geometries ∼3–4 nm in diameter and 50 nm in contour length. Small-angle X-ray scattering (SAXS) ensemble fitting confirms that a minority population of tubular conformers is required to reproduce experimental scattering profiles. Complementary atomistic MD and solution NMR chemical shift and relaxation analyses show that these tubular conformers are enriched in β-turn and bend motifs, maintaining local flexibility while promoting overall compaction. Mutational simulations further demonstrate that alternating poly(Ala) and Gly-Gly-X sequence patterning drives amphiphilic packing that stabilizes the tubular morphology. Together, these findings reveal that spider silk proteins form dynamic, disordered ensembles with sequence-encoded tubular substructure. This model reconciles SAXS and NMR observations and provides a mechanistic framework for how amphiphilic patterning, metastability, and disorder collectively enable spider silk proteins to remain soluble yet preorganized for hierarchical self-assembly into one of nature's toughest materials.

Significance Statement Spider silk proteins must stay soluble at extremely high concentrations inside the spider's silk gland while remaining ready to assemble into fibers stronger than steel. This study resolves a long-standing paradox: NMR data show that these proteins are highly dynamic and disordered, whereas X-ray scattering indicates compact, anisotropic shapes. By combining multiscale simulations with NMR and SAXS analysis, we show that spider silk proteins form dynamic ensembles that include transient tubular structures encoded by their sequence patterning. These metastable tubules explain how the proteins remain soluble yet preorganized for fiber formation, revealing general molecular design principles for creating adaptive, self-assembling biomaterials.

## Introduction

Spider silk has attracted scientific attention for decades because of its exceptional combination of strength, extensibility, and toughness—properties that surpass most synthetic materials. These remarkable fibers have inspired a wide range of potential applications in medicine, defense, and materials engineering (1–6). Among the multiple types of silk that spiders produce, the dragline silk used for lifelines and web frameworks is the most extensively studied. It is synthesized in the major ampullate (Ma) gland as a concentrated protein solution, or spinning dope, composed primarily of large structural proteins known as spidroins (1, 5).

Major ampullate spidroins (MaSp) are high-molecular weight (250–350 kDa) proteins built from highly repetitive core domains containing poly(Ala), poly(Gly-Ala), and Gly-Gly-X motifs, where X is typically a polar amino acid such as Gln, Tyr, Ser, or Arg (7). This repetitive organization gives the proteins a block-copolymer-like architecture, in which hydrophobic poly(Ala) regions alternate with more hydrophilic Gly-Gly-X segments (8). In contrast to the repetitive core, the MaSp termini are highly conserved, nonrepetitive helical bundle domains that are critical in the spider silk spinning process through a pH sensitive dimerization mechanism (9, 10). The conformational structure of the spidroin proteins in fiber form have been extensively characterized through solid-state NMR illustrating that the fiber is rich in nanocrystalline poly(Ala) and poly(Gly-Ala) β-sheet domains that can also include Ser while, the Gly-Gly-X repeats are believed to form disordered 3_1_-helical structures (11–20). A second dragline spider silk protein (MaSp2) contains similar β-sheet forming poly(Ala) domains and a unique Gly-Pro-Gly-X-X motif that folds into an elastin-like type II β-turn structure likely contributing to silk fiber extensibility (21).

Although the solid fiber structures of these proteins have been extensively characterized by solid-state NMR and X-ray diffraction, the solution-phase conformations of spidroins within the silk gland remain poorly understood. Solution NMR studies classify native MaSp proteins as intrinsically disordered proteins (IDPs), displaying high flexibility and minimal persistent secondary structure (22–26). Yet, small-angle X-ray scattering (SAXS) measurements reveal compact, anisotropic particles with a radius of gyration (R_g_) around 13 nm (27).

To explain how such large, disordered proteins remain soluble yet ready for fiber formation, it is essential to identify the types of tertiary structures that exist in the silk gland. While the fiber phase has been shown to contain ordered β-sheet nanocrystals formed by poly(Ala) and poly(Gly-Ala) regions (11–20), much less is known about the soluble state of MaSp proteins before spinning. In this environment, extremely high protein concentrations (∼30 wt%) are maintained without aggregation, implying the existence of self-limiting structural motifs that stabilize solubility while permitting assembly under changing conditions of pH, ion concentration, and shear (6, 28–30).

Early SAXS measurements suggested that MaSp proteins adopt compact, anisotropic shapes consistent with dimeric and elongated assemblies (27), whereas NMR studies indicated rapid backbone motion and limited secondary structure, typical of IDPs. This discrepancy points to a missing level of organization between local disorder and global anisotropy—potentially a transient or metastable substructure that has so far eluded direct observation.

Here, we integrate multiscale molecular dynamics (MD) simulations, published SAXS data (27), and solution NMR to investigate the tertiary organization of native MaSp1 and MaSp2 spidroins from the black widow spider (*Latrodectus hesperus*). Using both coarse-grained and atomistic models, we explore how amphiphilic sequence patterning drives folding behavior and compare simulated conformers with experimental scattering and relaxation data. This combined approach reveals that MaSp proteins form dynamic, disordered ensembles containing a minor but critical population of compact, tubular conformers that reconcile SAXS and NMR data. These sequence-encoded tubular architectures provide a structural basis for understanding how silk proteins balance solubility, metastability, and assembly readiness.

## Results and discussion

### Multiscale modeling of spidroin structure

**Fig. 1.**
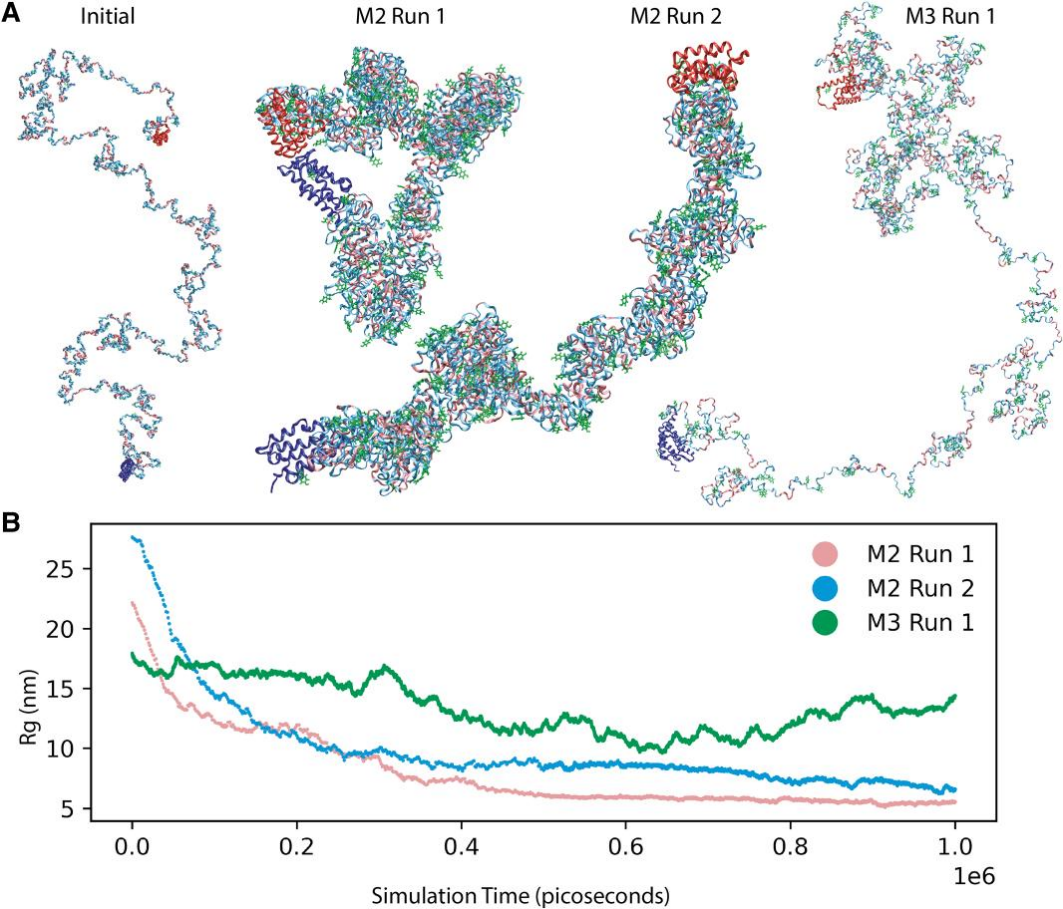
A) The full MaSp1 amino acid sequence modeled with a MARTINI2 (M2) or MARTINI3 (M3) CG force field as shown. The starting configuration of the tandem repeat region is a random coil with N- (blue) and C-terminal (red) regions taken directly from the protein database (PDB codes: 2N3E and 2KHM). For each run, a different randomly generated random coil starting structure was used as illustrated by the structure labeled “initial.” After 1,000 ns of simulation time, the overall shapes are vastly different, yet all the M2 simulations possess a consistent distinctive tubular character which is conserved across all the repeated simulations, that is absent in the M3 simulation where the protein remains disordered and unstructured but also highly anisotropic. The Tyr residues (green) are at or close to the surface in M2 models, while the hydrophobic poly(Ala) units (pink) are primarily buried in the core region. All other residues are colored light blue. B) The radius of gyration (R_g_) converged to similar values that are consistent with R_g_ determined by SAXS on closely related systems (see Discussion) indicating that there are unlikely to be further large-scale refinements to the structure although further folding at much longer simulation times cannot be discounted. Large-scale images of MaSp1 M2 simulations (SI Appendix, Figs. S4–S11), MaSp1 M3 simulations (SI Appendix, Figs. S12–S14) and MaSp2 M2 simulations (SI Appendix, Figs. S15–S19) and are shown in the SI Appendix.

**Fig. 2.**
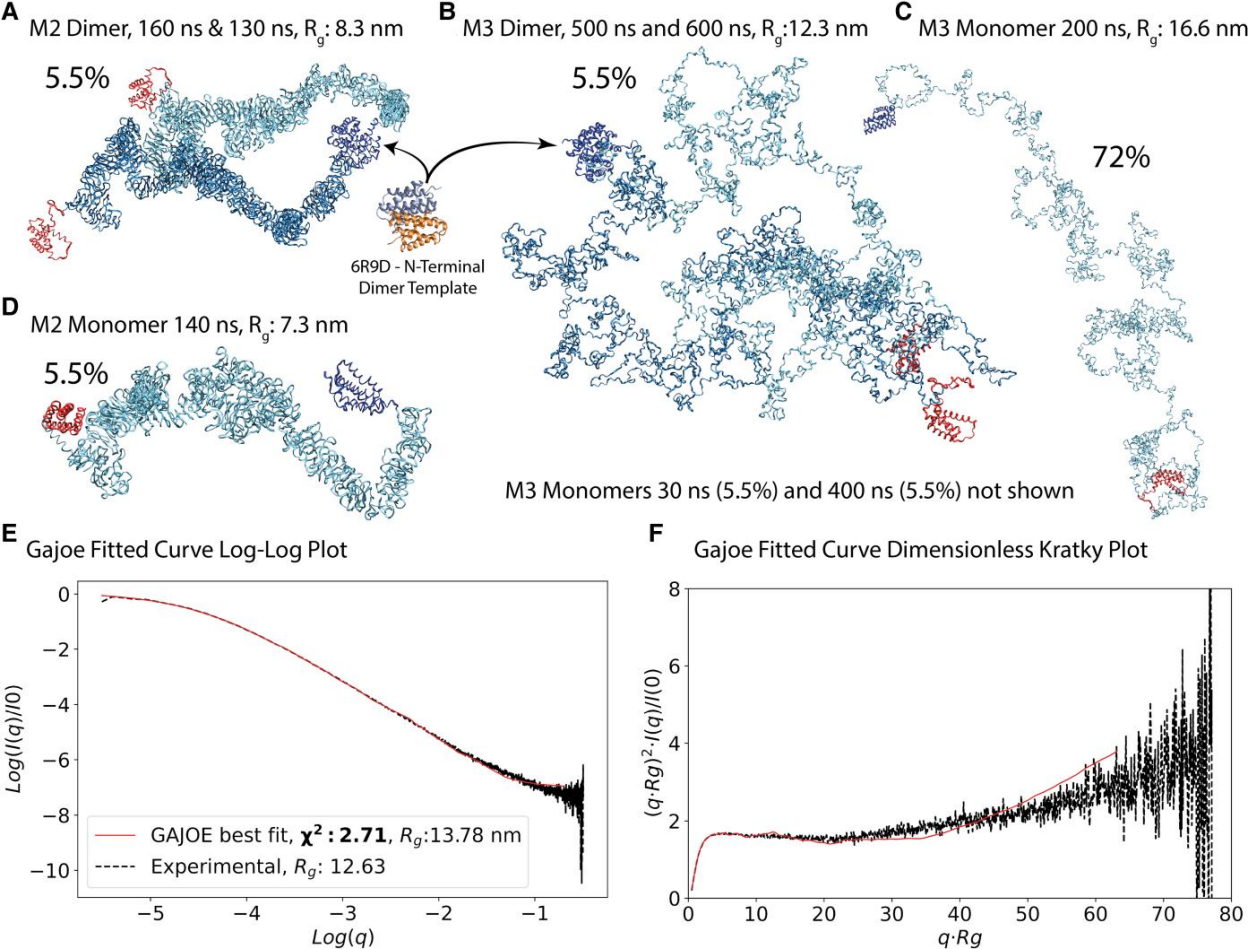
A–D) GAJOE (37) analysis of M2 and M3 conformations. For each conformation in the ensemble of 2,581 conformations (SI Appendix Fig. S1, and Table S1), the CRYSOL (36) software calculates a theoretical SAXS curve. The GAJOE software then uses a genetic algorithm to select a subset of the theoretical curves, and assigns a weight to each curve that reconstructs the experimental curve (black line). With a large pool of monomers and dimers GAJOE was able to find a constructed curve (red line) that fit the experimental data (27) (black line) with a χ^2^ value of 2.7. A–D) Shows five of the seven conformations chosen by the GAJOE algorithm and their associated weight. A) Martini 2 conformations at the 130 and 160 ns timepoints of the 1,000 ns simulation were joined into a dimer by aligning their N termini with a dimer crystal structure (6R9D). N-termini and C-termini are colored blue and red, and the main protein is colored merely to distinguish the monomers. The resulting structure exhibits a high degree of compaction (R_g_ = 8.5 nm) and the tubular structure is readily observed. B) An equivalent dimer pair from the Martini 3 simulations, at 500 and 600 ns time step is much less compact (R_g_ = 12.3 nm). C) An M3 monomer from 200 ns which is weighted by 72% in the ensemble is highly unfolded, R_g_ = 16.6 nm. D) To achieve the GAJOE fit the M2 monomeric tubule from 100 to 200 ns simulation time is necessary. E, F) Log–log plot and Kratky plots of the reconstructed curve plotted over experimental data. The experimental data are best reconstructed in the central region corresponding to mid-range structure from 1 to 10 nm around the knee of the curve in the Kratky plot. Shorter range structures to the right of the graph are much less well described by the model where the uncertainty is much greater. The total R_g_ of the combined ensemble (Guinier analysis of the red curve) was 13.8 nm, in reasonable overall agreement with the experimental R_g_ of 13.3 nm (27).

Our study employs both Martini-2 (M2) (31–33) and Martini-3 (M3) (34) MD simulations in combination with solution NMR and SAXS data. The combined results predict that MaSp proteins consistently adopt tubular shapes composed of disordered β-turns and bends with local dynamics. Generally, the tubular structures were predicted by M2 whereas the M3 MD models, by design (35), tended to predict a more open disordered structure (Fig. 1, SI Appendix Figs. S4–S14). To resolve these differences, and determine which model best described the available SAXS data (27), a pool of 2,581 diverse conformational structures—including both tubular and nontubular features—was generated. Theoretical SAXS curves can be generated for each conformation in the pool using the CRYSOL algorithm (36), which enables the GAJOE algorithm (37, 38) to attempt to recapitulate the experimental data (Figs. 2 and S1, and Table 1). By curating distinct pools with and without tubular structures, we were able to show that the experimental data is explained best when tubular structures are included in the pool. A Guinier analysis of the best GAJOE SAXS curve yields a measurement of R_g_ = 13.8 nm which agrees very well with the R_g_ = 13.3 nm reported from experimental SAXS measurements on native MaSp's, providing considerable credence to our results (27).

**Table 1.** Summary of **χ^2^** statistic from the GAJOE fitting process and the **R_g_ (**nm**)** from the Guinier plots of the GAJOE fitted curve.

Type	Pool size	χ^2^	R_g_
M2 monomers	29	75.1	16.7
M3–M3 dimers	841	38.4	24.8
M2–M2 dimers	841	12.8	8.9
M3 monomers	29	10.4	15.4
M3 monomers and M3–M3 dimers	870	10.0	18.0
M2–M3 dimers	841	6.5	15.1
M2 monomers and M2–M2 dimers	870	5.8	14.4
All	2,581	2.7	13.8

The only set which enables GAJOE to recapitulate **R_g_** is the full set of M2 and M3 monomers and dimers, which has the best **χ^2^** score. With monomers on their own GAJOE over estimates **R_g_**. M3 models on their own also result in over estimates of **R_g_**. M2 by itself requires heavy use of the unfolded starting model to reduce **χ^2^**. The M2–M3 hybrid dimers and M2–M2 dimer forces GAJOE to use the compact tubular structure in every conformation which results in a poor fit. Without monomers or the unfolded M3 structure, the M2–M2 dimers underestimate **R_g._** We conclude from the GAJOE analysis that a mixture of compact and unfolded monomers and dimers are needed to correctly reproduce the long-range experimental SAXS data. More details in SI Appendix Fig. S1 and Table S1.

Shorter lengths scales can be probed by analysis of ensemble structures, determined from solution-NMR data for a highly represented MaSp1 motif. This study illustrates a substantial fraction of MaSp's display a high population of β-turns and bends which helps to explain emergence of tubular tertiary shapes for the proteins. These results also agree with recent solution-NMR investigations for smaller recombinant Ma spider silk proteins where polyproline II helices and β-turns were determined in low population together with the more dominant random coil conformation (39, 40).

Altogether, this work proposes the first tertiary and secondary structure for Ma spider silk proteins which should be of significant interest to those designing spider silk mimetic biomaterials. It also provides a starting point to begin hypothesizing about the fiber assembly mechanism of spider silk proteins where these tubular structures are exposed to various physiochemical conditions (pH, ions, shear stress, dehydration) in the spinning duct of the native spider silk spinning system (6, 28–30). It is also an interesting case study for an IDP in which there exists a well-defined metastable locus on the range of conformations that may be adopted, which nonetheless does not possess a single clear native structure. In terms of energy landscapes such a system might be described as a metabasin containing many low-lying energy barriers. Such a well-defined but broad region of conformation space may be explored while still satisfying tubular long-range order. Such a model explains both the dynamics implicated in NMR data and the anisotropic structure revealed in SAXS experimental data. This overall picture yields the hypothesis that the tubular structure, driven by solvent interactions, is metastable, and can be destabilized by mechanical force and changing solution conditions in the silk-duct spinning process.

The principal reason for the previous absence of solution phase spider silk protein models is that most interest in spider silk has been directed at the postfibrillization solidified structure. Such silk solids contain non-β-sheet regions long thought to possess amorphous character, although disordered 3_1_-helical structures for the Gly-Gly-X region have been proposed as discussed above. These amorphous regions are not only challenging to access experimentally but detailed atomistic models are not expected to reveal much beyond confirming there was no structure to speak of in those regions, with structured domains being dominated by β-sheets. The latter has been shown extensively in the literature through MD modeling of the fibrous form where β-sheet nanostructures dominate (41–44). Modeling the silk dope before the fibrillization process is the goal here, and is entirely new, particularly for long sequences that mimic the whole protein. The coarse grain (CG) approach using M2 and M3 MD models is necessary because the length of the tandem repeat sections of the silk proteins saturates our computational capacity to model them fully in sufficient atomistic detail; the repeating central region of the MaSp1 protein is 2,880 amino acid residues long and 3,503 in MaSp2. Recent solution NMR analysis of smaller recombinant MaSp's suggests there may be important statistical organizational principles at work in these disordered regions that could be associated with preorganizational intermolecular interactions and the presence of protein micelle superstructures observed more recently by DLS and cryo-EM (26, 45). It is hypothesized that these superstructures could play a crucial role in the assembly and dynamics of fibrillization, and these observations created incentive to pursue models of these enormous proteins. The structure of the protein monomer in solution must be understood before hypotheses can be generated about the superstructural organization of the silk proteins that exist in the silk producing gland at very high concentrations (∼30 wt%) (26, 45).

The Martini framework (executed using GROMACS) models certain characteristics of the different residues, while reducing the degrees of freedom of the problem, so that it could run on Nvidia GeForce Ti1080 GPUs within a reasonable time frame (∼1 month). Using this approach, M2 predicts remarkable tertiary structures for both MaSp1 and MaSp2, consistently yielding a conserved tubular architecture and revealing specific roles for residues in the non-β-sheet-forming regions. In contrast, the more complex M3 model exhibits a broader range of behaviors, having been specifically reparameterized to better represent a broad range of proteins including IDPs (34, 35). In our M3 simulations, the large proteins adopt more solvent-exposed, elongated conformations characterized by disordered anisotropic clusters connected by long, flexible linker regions (SI Appendix, Figs. S12–S14, Table S2).

Both the compact tubular and disordered but anisotropic structures are always selected by GAJOE to explain the experimental data when they are available. Solution NMR studies agree and show that one extremely common MaSp1 repetitive motif forms a loose turning coil rich in β-turns and bends that helps to explain the tubular simulation results. Fully atomistic simulations on short (15–100 residue) and medium (474 residue) length sequences agree with the high β-turn and bend content found through solution NMR while still exhibiting local backbone dynamic properties consistent with NMR relaxation. A further mutational study using GROMACS M2 simulations conducted on A100 GPUS at the NU Quest facility demonstrated that the tubular structures require only 400 amino acid residues to emerge, and that comparing distinct knock-out sequences demonstrated quite different behavior using the same M2 model, thus establishing hypotheses for the role of the different residues and their sequence position in the emergence of the tubular tertiary structure. Here, we show that the amino acid sequence of MaSp proteins encodes a metastable tubular substructure that resolves the SAXS-NMR paradox and represents a preorganized intermediate in silk assembly.

CG-MD simulations were conducted with both the MARTINI V2.6 and MARTINI 3.0 force field for the full-length repetitive core of *L. hesperus* (Western black widow) MaSp1 (Fig. 1, and SI Appendix, Figs. S4–S14) and MaSp2 (Figs. S15–S19). Four conformations (Fig. 1) are shown from a total of nine MaSp1 simulations that were all run from distinct random coil initial structures for the core repetitive region. In six simulations, structured terminal regions were taken from the PDB (2KHM for C-terminus (10) and 2N3E for the N-Terminus (46)) and appended to the unstructured core after the simulation. One simulation was run with the wild-type sequence (7) which had completely unstructured termini included at the outset and a video of this trajectory is included in the online database of results. An eighth M2 simulation consisting of just the core region of the silk was also conducted, with the same N- and C-termini manually added using the PDBSTAPLER algorithm. These structures were used later to form the M2 dimers. The ninth simulation used M3 to simulate the core region, and termini were added later also using PDBSTAPLER. Regardless of the starting configuration and the presence or absence of unfolded termini, a consistent tubular tertiary structure emerges in all M2 simulations, with *selective partitioning of specific amino acid motifs* in all cases. While the M3 simulation more closely reflects the expected behavior of disordered proteins (35), they did not yield compact tubular structures, even over long timescales. In contrast, M2 consistently produced anisotropic tubules that matched key experimental observables. We therefore used M2 to populate the conformational pool with shape-anisotropic models necessary to explain SAXS data. This highlights the value of model diversity: although M3 better captures unfolded chains, M2 uniquely samples compact geometries that contribute to the full solution-phase ensemble. A more compact structure is to be expected in the M2 model, as the beads tend to give stronger protein–protein interactions that over-power the solvent interactions.

Indeed, a full investigation into how such tubular morphology arises from intrinsic sequence features was conducted via a series of mutational simulations within the M2 framework. These systematically altered the distribution of Ala, Gly, and polar residues (e.g. Tyr, Gln) to test how the native amphiphilic block pattern governs tertiary structure. The results demonstrate that tubule formation depends on maintaining alternating hydrophobic and polar domains, with polar residues stabilizing the surface and Ala-rich regions driving internal packing. Thus, the emergence of the tubule is governed strongly by such protein–protein interactions. In contrast, the increase in the number of beads and model choices for M3 interactions allow more nuanced emulation of the protein–solvent interactions for a wide range of systems including in IDPs (35) particularly the nonbonded interactions which helps to overcome the excessive protein–protein interactions that tend to collapse M2. In contrast the tubule does not form in the M3 simulation although, as indicated by geometric analysis of the structures (SI Appendix, Table S2), the M3 structures are still highly anisotropic and not spherical, even towards the end of the simulation trajectories. They are best described as anisotropic proto-tubular clusters with long linker regions.

In the M2 tubule structures, the poly(Ala) is primarily buried and specific polar amino acids (Gln, Tyr, Arg) are located almost exclusively on the protein surface (statistical analysis below). The simulations, which deal only with a single monomer, yield a radius of gyration, R_g,_ as a function of simulation time, as shown in Fig. 1B. Interestingly, for M2 the R_g_ of the monomer reaches a rough plateau after around 100 to 400 ns simulation times, with the minimum R_g_ between 5.4 and 8.5 nm. This value is very similar to experimental SAXS studies that determined R_g_ to be 8.8 nm for closely related silk fibroin (47). SAXS studies have also appeared on Ma silk proteins in solution where R_g_ was determined to be 13.3 nm (27). This larger R_g_ is due to protein dimerization as discussed in the reference. The terminal groups are known to form dimers (9, 10) and an experimental structure of this dimerization exists (46, 48) (PDB 6R9D) which enables us to align the N terminal region (2N3E) of two monomeric tubular structures drawn combinatorially from different time points in the simulation into dimers using the PDBSTAPLER algorithm (Fig. 2A and B).

NMR reveals that spider silk proteins exist in a conformationally heterogeneous ensemble, lacking persistent secondary structure and exhibiting dynamics akin to those observed in IDPs (25). The aim rather, is to determine what range of features must be present in such a statistical soup to explain the experimentally observed SAXS data (27). Thus, the goal of the simulations is to generate diversity in the ensemble behavior that we can include in the structural pools, rather than to “perfectly model” the proteins. As discussed above, the M2 model consists of only a handful of CG beads and is known to favor more compact structures (34, 35). The M3 CG system has a much wider range of bead types that more closely capture protein–solvent interactions in a wide range of systems (34). Furthermore, the secondary structure of these kinds of models is determined at the outset. At this stage, we are less interested in the atomistic details and more concerned with the nature of the long-range tertiary structure that we can include in our pools to explain the 1–10 nm mid-range SAXS data.

Eight distinct pools of conformations were developed by taking conformations from a range of sources. Structures were taken every 10 ns between 0 and 200 ns from both an M2 and M3 trajectory. A further set of eight fully “folded” tubular structures were added to the pool for M2 simulations from previous trajectories, and a further set of eight structures were taken every 100 ns from 300 to 1,000 ns from the M3 trajectory. The result is a set of 29 M2 and 29 M3 conformations to which termini were added using the PDBSTAPLER algorithm as necessary. Dimers could then be constructed by drawing pairs of monomers from the pool of 29 and aligning their 2N3E N-termini regions with the 6R9D structure. The result is a set of 841 M2 dimers, 841 M3 dimers and 841 M2–M3 hybrid dimers. When combined with the original 29 M2 and 29 M3 monomers, we yield eight pools.

### SAXS ensemble analysis

The CRYSOL algorithm (36) was used to compute theoretical SAXS curves for each conformation in the pools. Experimental SAXS profiles for native MaSp proteins were taken from Greving *et al.* (27) and used as the reference dataset for all ensemble-fitting analyses described below. No water shell fitting was included against the experimental data so the SAXS curves are purely theoretical. From each ensemble of SAXS curves, the GAJOE algorithm (37) then selected subsets containing 1 to 50 curves that collectively best describe the experimental data for MaSp1 spidroins (Table 1, SI Appendix Table S1 and Fig. S1). Such conformations can be represented multiple times in the subset of 1–50 structures, yielding a weighting for that conformation. Table 1 shows the **χ2** statistic for how well the selected set of conformations chosen by GAJOE from each of the pools match the experimental curve—in most cases, very badly. It is clear from the Table 1 that both monomeric, dimeric, tubular, and unfolded structures are necessary to fully explain the experimental SAXS data.

SI Appendix Table S1 provides additional details about the sets of conformations that the GAJOE algorithm selected. The conformation descriptors take the form of MxMy_xxxx_yyyy or Mx_xxxx where Mx and My refers to either M2 or M3, and xxxx refers to a time stamp in the simulation trajectory (0 to 1,000 ns). Generally, the compactness of the M2 structures depends on the time stamp in the simulation, which is clear from the R_g_ curve in Fig. 1. M2-M3_0010_1000 refers to a dimer comprised of an M2 model at 10-ns of CG simulation and M3 model at 1,000 ns of coarse-grained simulation.

Taking each pool in turn, we can see that M2 monomers (nearly all full tubules) are the least favorable group on their own with a **χ2** statistic of 75.1. Furthermore, GAJOE was obliged to assign a weighting of 88% to the completely unfolded starting structure (M2_0000) that was completely random and had not yet been subjected to the MARTINI force field at all (see SI Appendix Methods). In other words, with 29 mostly compact tubules drawn from a range of 0 to 1,000 ns folding time, GAJOE cannot recapitulate the experimental curve using its genetic algorithm. R_g_ is also overestimated. In contrast the M3 monomers alone fare much better but are still a poor fit (**χ2**  **=** 10.4) and over-estimate R_g._ With M3, GAJOE does not rely on the starting structure as the M3 models are much more unfolded.

A similar comparison of the M3–M3 (**χ^2^**  **=** 38.4), M2–M2 (**χ^2^**  **=** 12.8) and M2–M3 (**χ^2^**  **=**  **6.5)** dimers in Table 1 shows that M2–M2 and M2–M3 have a heavy weighting for the unfolded uncompact models, whereas, the M3–M3 dimers, which are highly disordered, are chosen across the time scale. Altogether, these observations not only indicate that unfolded/disordered structures are necessary to explain the data, but also that dimers are necessary. However, dimers alone are insufficient. M2–M2, dimeric M3–M3 together cannot explain the data. Dimeric M2–M3 performed better than other classes but ultimately only had a **χ2** of 6.5.

Mixtures of monomers and dimers drawn from the same model did not reproduce the experimental SAXS data well. For example, the best fit using only M2 structures yielded a χ² of 5.8 but consisted primarily of unfolded chains, with 86% of the ensemble in fully extended conformations and a combined R_g_ of 14.4 nm (SI Appendix Table S1). Similarly, ensembles drawn exclusively from the M3 pool failed to explain the data, selecting a wide range of unfolded monomeric and dimeric structures from across the trajectory. In contrast, the best-fitting ensemble (Fig. 2) required a combination of folded tubular and disordered structures across multiple models. This ensemble included M2 dimers (160/130 ns), M3 dimers (500/600 ns), M2 monomers (140 ns), and M3 monomers (30, 200, and 400 ns), in a 5.5:5.5:5.5:83 ratio. The resulting R_g_ closely matches experimental values. While the disordered M3 monomer was the most abundant component (83%), the inclusion of both monomeric and dimeric tubular structures (11% total) was essential to achieve a high-quality fit.

### Structural characteristics of tubular conformers

Having observed consistent tubular shapes between different simulations and determining that they are required to sufficiently fit the experimental SAXS data, we can characterize the tubule structure with a range of deeper geometric analysis. SI Appendix Figures S2 and S3 and Table S2 reveal a hierarchical structure of the tubule that is characterized by three different lengths scales for MaSp1. We also ran some simulations for MaSp2 proteins and obtained similar results. Each parameter is defined in detail in the Methods section and SI Appendix. The longest scale is *averaged contour length* (ACL) which relies on the determination of a central tubule axis that matches our intuitive notion of the tubule shape. Such a curvilinear axial trajectory can be determined by taking a local center of mass of 150 residue sequences every 10 residues along the primary protein sequence (SI Appendix, Fig. S2). Such a trajectory smooths out detailed backbone configuration and characterizes the CG model. This approach works because the sequence order is approximately the order that the residues are packed into the tubule. Averages over fewer than 150 residues did not remove sufficient fine detail about the protein backbone, whereas averages over 400 residues lost information about the tubule shape. These considerations suggest an appropriate size for an intermediate CG unit between 150 and 400 residues, corresponding to 5 to 13 repeats of the amphiphilic sequence patterns.

The MaSp1 ACL is around 50 nm yielding a size parameter for the tubule structure which matches our intuition of the length of the tubule structures and corresponds to the length scales for monomeric units suggested elsewhere for closely related silkworm silk fibroin (47). The full length of the protein backbone (∼1,160 nm long for MaSp1, ∼1,400 nm for MaSp2) is considerable and is packed into this 50 nm tubule. The second length scale is the *Max Euclidean* (MaxE) length between any two atoms within the structures. For our MaSp1 structures, Max E is around 16 nm. The overall gradient of the R_g_ plateau suggests longer time scale simulations would find more compact folds of the tubule still. In vivo such higher order folding is sure to be impacted by neighboring proteins in the silk dope that are not present in the simulations here. The third and final characteristic length scale is the tube radius, R_t_, which is between 1.6 and 2 nm for the different conformations analyzed. We define R_t_ as the 90th percentile of the distribution of radial distances between each residue and the nearest point on the axis at the center of the tubule (SI Appendix, Fig. S3 and Methods). The measured diameter for the MaSp1 and MaSp2 tubules is therefore between 3.2 and 4 nm. Intriguingly, this value is very close to the diameters of nanofibrils found in silkworm (49) and Ma spider silk (50) fibers suggesting these nanofibrils could be representative of individual proteins.

In view of the order of magnitude span of hierarchical lengths that characterize the tubule (ACL, MaxE, and R_t_), it is clear that conventional experimental measurements of protein size such as R_g_ (radius of gyration) and R_h_ (hydrodynamic radius) mask significant details about the shape and nature of the spidroin molecule. However, R_g_ and R_h_ do enable meaningful comparison of our models with the literature (51). Taking the ratio of R_g_ and MaxE yields a value of 0.3 which is generally taken as a good figure of merit for the structural anisotropy. The anisotropy of our structures matches the anisotropy of silk dope as measured using SAXS where a value of 0.3 was also found (27). That said, the anisotropy defined as R_g_/MaxE is relatively consistent across all structures we simulated, including the M3 structures (∼0.3) and is therefore a poor discriminator for understanding differences in our structures. We found that R_g_/R_t_ and R_g_/R_h_ (the shape factor) were better order parameters for capturing anisotropy differences between our simulated structures. For a globular (spherical) protein, R_g_/R_h_ is predicted to be ∼0.77 while a coil shape has a value of ∼1.0, and ∼1.7 is an extended rod. The values determined for the simulated structures indicate that the coil is a good description of MaSp1 and MaSp2 monomers which have shape factors in the region 0.9–1.1. The supporting information contains large representations of each conformation, each labeled as in SI Appendix Table S2 (SI Appendix Figs. S4–S19).

As a result of this simple analysis, we expect that models with 400 residues or more should exhibit tube-like properties, since they are sufficiently long to comprise of more than one 150–200 residue sub-units. On their own, such 150–200 residue structures tend to be compact globular structures. Such an observation is borne out in the mutational simulations and size assay discussed below, where shape factors range from spherical to coil depending on the simulation. The R_g_/R_t_ ratio also reveals insightful trends.

For all the tubule structures under consideration, the identity of solvent-exposed residues was determined by the rolling ball method using the GetArea algorithm (52) and the results are presented in SI Appendix Table S3 for M2 and M3 MaSp1 and MaSp2 proteins, and the atomistic simulations. A set of eight MaSp1 conformations, drawn from six M2 simulations, and four MaSp2 conformations, drawn from four M2 simulations, were analyzed, and three M2 MaSp1 proteins at 50, 150, and 1,000 ns. The surface areas for each residue type were summed for each conformation, as well as the relative populations of inside and outside residues of that type.

There are relatively few residue types found in the spidroin proteins. 95% of the sequence is drawn from Gly, Ala, Gln, Tyr, Ser, and Arg in MaSp1 with the addition of Pro in MaSp2. The bulk of the residues are Gly and Ala (75.0% MaSp1 and 64.6% of MaSp2). A simple model (see Methods and SI Appendix) for packing spheres into cylindrical shells suggests a 70–30 split between bulk and surface residues for geometries matching our tubule parameters (50 nm long cylinder with a diameter of 4 nm) and total number of amino acids found in the MaSp proteins (3,102 for MaSp1, 3,779 for MaSp2, and 2,880 for the time series core sequence). The values arising from this simple model are in close agreement to results from solvent exposure analysis when *all* the residues in the protein are considered (blue text in SI Appendix Table S3). On average, 66% of the residues are buried in MaSp1 and 15% solvent exposed. The remaining 19% possess similar solvent exposure ratios as their standard random coil value, and thus lie somewhere between buried and exposed, i.e. near the surface. For MaSp2, these figures are 68%, 14% surface exposed, and 17% somewhere between exposed and buried. Thus, both MaSp1 and MaSp2 solvent exposure analysis agrees well with the 70/30 split from our geometric model (SI Appendix, Figs. S2 and S3). Other dimension cylinders have considerably different surface to volume ratios. Remarkably, the time series shows that these values are adopted very quickly and remain highly conserved from 50 ns onwards.

Comparing sub-populations of different residue types against this standard 70/30 split is illuminating. For MaSp1 we see that Gly and Ala are significantly over-represented in the core (∼76%) and under-represented on the surface (∼22%). Gln, Tyr, and Arg are considerably under-represented in the core with only around 20–50% of those residues within the interior, but 30–31% are fully solvent exposed, and 20% partially exposed (i.e. somewhere between a 20–80 and 50–50 split for interior to exterior). For MaSp2 the situation is similar. Gly, Ala, and Ser all have ∼80% in the core and 5–11% in the surface, compared with Pro, Gln, Tyr, and Arg showing between 20–40% in the core and 17–36% located in the surface region. It is also noteworthy to mention that SSNMR has shown that Ala, Gly, and Ser comprise the nanocrystalline β-sheet domains in *L. hesperus* Ma fibers (19) and the simulated models show that these domains are primed for β-sheet assembly by being buried in the solvent-poor, protein core. Strikingly, in our models, Ser changes character from a surface to a core location between MaSp1 and MaSp2. Pro adopts mostly a surface role as expected. In MaSp1 only 56% of Ser is in the core, compared to 76% in MaSp2. Ser occurs in significant numbers in both proteins.

Finally, it is worth noting that although the bulk of the Ala, and Gly residues are indeed within the core of both proteins, there are still substantial numbers of Ala and Gly with solvent accessibility. In fact, they form some 40% of the outer surface. This may be critical for interprotein interactions in building fibril formations between protein chains, suggesting a role for the outer residues such as Gln, Tyr, and Arg in driving and maintaining the formation of a tubular structure as a means of managing interprotein contacts. Although speculative, this is an interesting prospect that can be tested with future modeling of multichain interactions. These polar surface residues may also play a significant role in mediating the supramolecular assembly (45) of MaSp proteins in the gland and possible liquid–liquid phase separation (53, 54) intermediate steps in the silk spinning duct. The two latter processes are believed to be critical for fiber spinning and are only being explored more recently.

SI Appendix, Fig. S20 shows the results of a simple size assay in which 100, 200, 400 residue sequences were simulated to see how the length of the sequence affected the final geometric tertiary structures. We observed that the tubule structure has emerged by the time the sequence is 400 residues long. The 100 and 200 residues sequences were more like compact and spherical globular proteins which is further evidence to support the idea that the appropriate minimal CG unit for higher order exploration of the tertiary and quaternary structures would be in the 150–200 residue range. Similar numbers emerged spontaneously in our averaging procedure to determine the axial trajectory of the tubule. Such an analysis also supports using 400 residue sections to analyze the radius of the tubule, as this is the minimal length at which the tubule emerges.

### Sequence dependence of tubule formation

A set of MaSp mutants were simulated (SI Appendix, Fig. S21) to reveal the role that each class of residue plays in the emergence and detailed character of the tubule structure. These mutations test whether the tubular tertiary structure arises from intrinsic features of the native sequence. By systematically varying the Ala:Gly ratio and disrupting the placement of polar residues (e.g. Tyr, Gln), we show that the tubule only emerges when the wild-type block-copolymer-like pattern is preserved. In mutants lacking this pattern—such as all-Gly, all-Ala, or randomly scrambled sequences—the resulting structures are either spherical, amyloid-like, or highly disordered. A sequence of 474 residues taken from the wild-type sequence does indeed form tubular structures as expected. The sequence chosen is taken from the main body of the native sequence, and consists of 16 Gly-rich and poly(Ala) repeating motifs. The details of the modeled mutations reveal hypotheses for the nature of the amphiphilic pattern in the primary sequence. Why should there be 5 to 10 residue repeats of Ala and 17 to 25 residues for the Gly-rich repeat unit, and what is the role of the disrupting X-residues such as Gln, Tyr, and Arg which our model suggests are predominantly surface moieties? The starting structure for each of the mutational simulations was a random coil similar to the one shown in Fig. 1A and in the videos in the data repository associated with this work.

When the sequence is 474 Gly (SI Appendix, Fig. S21A) we see the radius of the final tubule is a little narrower than the wild type with a smaller R_g_. However, it is significantly more ordered. The residues bundle into β-sheet-like stacks forming a cuboid shaped tertiary structure with a shape factor of 0.83 (SI Appendix Table S2, R_g_/R_h_ column) which is highly compact and spherical. The details of the highly organized packing are reminiscent of amyloid structure. The length of these Gly strands are around 10 residues long (∼3 nm), which is as long as the poly(Ala) sub-blocks in MaSp proteins. As the structure folds throughout the trajectory, we see it coalesce into beads of 245, 55, and 174 residues whose internal structures end up as highly organized sub-domains that we have colored distinctly in the interim trajectory structures in the figure. Such sizes of intermediate structures are again consistent with the idea that 150–200 residues long are the minimal CG unit.

A similar result happens when the structure is purely Ala as shown in SI Appendix, S21B. In this case the resulting organized strands are 15 to 20 residues long (3.5–4 nm), which is about the length of the amphiphilic blocks of Gly. By the end of the trajectory the pure Ala structure forms a single organized domain of aligned strands with a shape factor of 0.81 (SI Appendix Table S2), categorized as spherical. We note that in our model the Gly strands naturally form structures of the size of the poly(Ala) domains in the spider silk, and that the Ala strands naturally form structures about the size of the Gly domains. We do not know if this is significant or merely a coincidence; the chemical character of Ala and Gly is similar.

When we create a pattern consisting purely of alternating blocks of pure Gly or pure Ala in precisely the same block structure seen in the native silk (we achieve this by mutating all non-Gly or non-Ala residues into Gly) we see similar single domain structure emerge which, again, is almost a cuboid volume and consisting of entirely aligned strands (Fig. S21C). This structure has an R_g_/R_h_ value of 0.77 indicating it is spherical in nature (SI Appendix Table S2). It is likely that the identity of the Gly and Ala is irrelevant in this structure, as it forms very similar structures to pure Ala, and pure Gly alone. However, detailed visual inspection of the structure in VMD (55) suggests that the Ala strands are quite well isolated from each other by Gly regions with fewer Ala-Ala region contacts. This observation suggests that a principle of frustration may be at work, in which the block pattern is selected to disrupt the intrinsic structures that each type of residue adopts singly. Perhaps the irregularity of the repeating pattern is an emergent moiré pattern between the competing natural sizes for each kind of residue and the block-like nature of the pattern in which sometimes the Ala regions occupy a loop, sometimes strands. This pattern would ensure that some Ala are on the surface and not buried in the core. We note that the overall diameter of the mixed Gly-Ala structure is midway between the diameters of the pure Gly and pure Ala structures.

The picture changes radically when we create a sequence in which there are no Ala at all, yet we leave all the disrupting polar X-residues in the structure (SI Appendix, Fig. S21D). In this case, the structure first forms a tubule which then readily collapses and forms a compact globule in which there is no long-range anisotropic structure at all. The shape factor is 0.75 which is the most spherical shape in any of the simulations conducted. The inset highlights the backbone cartoon which contains only hairpin turns throughout, with no β-strand forming regions or alignment between the strands of the type we saw in the first two mutational models. Such cartoons in VMD (55) are quite sophisticated and show the result of analysis with the Stride algorithm (56) which identifies β-strand and α-helical regions. Therefore, our model suggests that spacing of the non-Ala and non-Gly residues has the effect of disrupting the formation of a regular strand-like structure. This is an obvious test target for future studies to see how the amount, number and distribution of X-residues affect the formation of a regular amyloid-like alignment between the poly(Ala) blocks of the spider silk proteins.

It is only when we add all the components—the Ala and Gly in a block pattern and the X-residues—that suddenly a tubule structure re-emerges. Both the Tyr knock-out structure (SI Appendix, Fig. S21E) and the wild type (SI Appendix, Fig. S21F) have shape factors that jump back towards coil (SI Appendix, Table S2). The Tyr knock out shape factor is 0.86 and the Wild type 0.89. It is intriguing to note that the spacing of the disrupting residues, such as Gln and Tyr, (within the Gly blocks) is such that a portion of the Gln and Tyr are exposed to the external surface of the protein. In the Tyr knock out (SI Appendix, Fig. S21E), we see that the tubule structure is still present suggesting that Gln contributes significantly in stabilizing the tubule structure. The Gln residues are predominantly on the surface and there is a much less organized character to the turn and strand structure of the protein. An energetic analysis of different structures may be useful to estimate the stabilizing contribution of Gln-Gln, Tyr-Tyr, and Gln-Tyr contacts on the surface. Thus, we conclude the Tyr and Gln disrupt the natural propensity of the Ala and Gly to form long 3–4 nm strands, resulting in a much more tubular shape with similar radius but much less preorganization between the Ala-rich regions. The latter could be responsible for preventing premature β-sheet aggregation of the proteins in the spider silk gland environment.

The three scrambles (SI Appendix, Fig. S21G) show that a randomized sequence also forms tubules (Shape factor 0.87, 0.93, and 0.97), but there is quite a wide variance in shape factor that brackets the wild type (SI Appendix Table S2). We note that in scramble 1 all the Tyr randomly collected in one half of the structure. This asymmetry we believe resulted in a kink emerging in the tubule, as one half did not have the stabilizing effect of the Tyr at the surface. Although such kinks were observed elsewhere, they were somewhat less pronounced. This experiment of opportunity suggests a hypothesis that the long-range persistence length of the higher order tubule may be affected by the distribution of the disrupting residues.

The results from the second panel of SI Appendix, Fig. S21H–K support the emerging picture. In this sequence of simulations, we selectively rearrange the sequence and move entire blocks of Ala rich regions around, while keeping the overall proportions of residues the same. In this way, we change the block pattern of the protein. We see that when the poly(Ala) is in a single continuous stretch, as in SI Appendix, Fig. S21H, they cause the tubule to have a much larger radius at one end than the other, resulting in a shape factor of 0.81 which is moving back towards spherical (SI Appendix Table S2). This result is intriguing. If there is too much Ala to dominate the structure (as in SI Appendix, Fig. S21B, H, and I) the shape factor becomes more spherical. As we systematically redistribute the Ala back into the 16-mer pattern of the wild type, we see a systematic change in shape factor back to coil. Thus, SI Appendix, Fig. S21J and K in which there are two and four regions of Ala, there is a more coiled character. This trend is direct evidence that the distribution of the Ala-rich regions directly controls the overall geometry of the tubule and that there is a delicate balancing act between the distributions of Ala, Gly, Tyr, and Gln, which yields a tubular structure that is at least metastable.

In summary, we have shown that the interplay between Ala- and Gly-rich regions drives the formation of a tubule-like structure that would probably be akin to amyloids. The polar X-residues of Tyr and Gln disrupt the formation of aligned strand-like structure and force the backbone into tight turns, with a slightly smaller radius tubule. The amphiphilic, block-like nature of the protein drives the overall radius and, due to the hydrophilic nature of Tyr and Gln, these disrupting amino acids are forced to the surface where they may play a role in stabilization and interaction with adjacent proteins and solvent.

Future work in this analysis would be to investigate the energy barriers, and therefore the rates, separating tubular from nontubular conformations and how the distribution of the disrupting residues affects the energy landscape, as well as investigating different proportions of Ala to Gly ratios. Preliminary work in the latter endeavor is included in the supporting information (SI Appendix Fig. S22), in which we change the overall ratio of Ala:Gly in the protein, which does not seem to systematically affect the structure as much as moving the poly(Ala) blocks to other parts of the protein.

In one further attempt to investigate the geometry and structural significance of our system, AlphaFold2 predictions (57) were made for 200, 400, 800, and 1,600 residues drawn from the MaSp1 and MaSp2 sequences (SI Appendix, Figs. S23 and S24). Their geometric results, as conducted with the same analysis we applied to the MARTINI models, are included SI Appendix Table S2 and their figures of merit can be found in SI Appendix, Table S4. The standard pLDDT figure of merit for the simulation is around 0.4 indicating that AlphaFold has low confidence in its prediction (57), which is typical for an IDP. The prediction for the N- and C-termini have much higher confidence (90% for the C-terminus, and 68% for the N-terminus, although the N terminus included a large part of the linker sequence to the main body of the protein). Although AlphaFold remarkably predicts a tubule structure with a radius around 2.5 nm, and similar R_g_'s to our CG-MD simulations with M2, it predicts a very different distribution of residues with regards solvent accessibility. The resulting AlphaFold structures are not physically realistic with respect to the solution phase MaSp structure, but rather predict high β-sheet propensity as expected for the structure of fibrous silk (19). Although intriguing, these AlphaFold results highlight their potential for modeling the solid-state fiber structure and emphasize the importance of other categories of modeling besides AlphaFold, particularly for the IDP solution phase MaSp structure.

### Experimental NMR validation

To probe the relevance of the simulated protein architectures in native Ma silk dope, we used solution NMR to obtain experimental structural data. Solution NMR chemical shifts are a long established and widely used source of secondary structure information (58–61). When combined with sequence information and database-based search analysis of published structures, these chemical shifts can provide full 3D solution structures in many cases. We therefore employed solution NMR to characterize secondary structure and determine low-energy structural ensembles for native MaSp proteins from NMR chemical shifts to further compliment the MD simulated structures with experimental data. The determined solution NMR chemical shifts were used as inputs to NMR computational tools TALOS-N (61) and CS-Rosetta (62 , 63) for secondary structure characterization and structural ensemble determination. Ma glands were enriched in NMR-active nuclei (see Methods), excised from *L. hesperus* spiders, and used for a broad range of multidimensional structural NMR experiments discussed below.

**Fig. 3.**
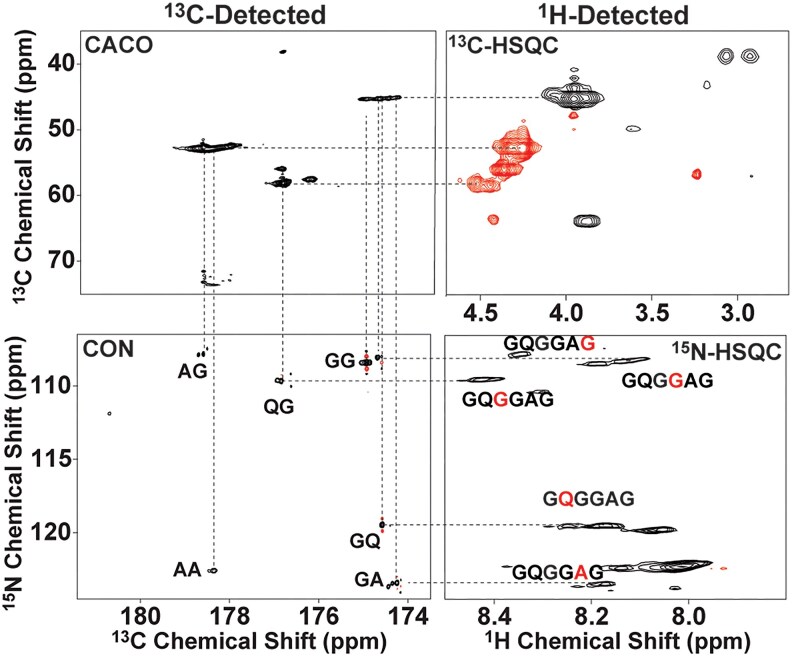
Solution NMR spectra collected for isotopically enriched (^13^C/^15^N) intact Ma glands excised from Western Black Widow (*L. hesperus*) spiders. Sequential assignment for pairs of residues from the correlated X-detect experiments CACO, CON, and traditional ^13^C- and ^15^N-HSQC are shown. The X-detect data were collected at 800 MHz using an X-detect optimized TXO cryo-probe and the ^1^H-detect experiments were collected at 600 MHz with a RT HCN solution NMR probe.

The biggest challenges with NMR analysis of spidroin proteins stem from many factors including their massive size (250–350 kDa), highly repetitive block copolymer nature, and intrinsically disordered conformational state. This results in extremely low chemical shift dispersion and extensive broadening of the observable signals that cannot be overcome simply by going to higher field NMR (although we do see some increase in resolution at high field). In order to tackle these issues, we applied an arsenal of multidimensional experiments including traditional ^1^H-detected 2D and 3D NMR together with 2D X-detect correlation experiments CACO and CON (64, 65). Figure 3 shows the correlation between the two X-detect experiments, and their 2D ^1^H-based complements. The broadening and overlap in the ^1^H-detect experiments can be successfully separated out using the X-detect experiments, which allows for sequential assignment for residue pairs. Two residue carbonyl assignments were made using this combination of X-detected CACO and CON experiments for residue pair sequential assignments. These pairs of assignments were then added to the sequences based on their order of appearance in the full *L. hesperus* MaSp1 sequence (7). Traditional 3D NMR experiments were used to make sequential assignments for noncarbonyl nuclei in a 15-residue repeat sequence of MaSp1. A full 6-residue assignment from the Gly-Gly-X region was assigned and then placed alongside assignments from the poly(Ala) region (see Methods). This resulted in a nearly complete backbone nuclei assignment for the following sequence: GQGGAGAAAAAAAAG. This sequence repeat is highly represented throughout the repetitive core of the *L. hesperus* MaSp1 sequence and appears 39 identical times in the sequence comprising ∼20% of the repetitive core (SI Appendix, Fig. S25). This 15-residue domain contains the Gly-Gly-X (X = Gln) repeat and a long poly(Ala) block containing eight Ala. We have previously reported the chemical shift assignments for five of the backbone nuclei in this short sequence (26). Now we complete the assignment with the addition of the carbonyls (SI Appendix, Table S5).

From these NMR chemical shift assignments, a combination of computational techniques (TALOS-N and CS-ROSETTA) were used to make determinations about probable structural ensembles from the NMR data and conduct secondary structure analysis. The top 10 lowest energy CS-ROSETTA structures determined for the 15-residue domain and longer 120-residue (comprised of eight 15-residue repeats linked together) are shown in SI Appendix, Fig. S26 along with their Ramachandran plots. Close inspection of the Ramachandran plots reveals a high population of type I and type II β-turns with very low β-sheet and helical content (Appendix SI, Fig. S27), which is consistent with the MD models presented above. The top 10 lowest energy CS-ROSETTA structures for the 15-residue and 120-residue repeats are shown in the SI Appendix Figs. S28 and S29 along with color coded secondary structure assignment. Complete secondary structure quantification for the CS-ROSETTA structures was conducted with the DSSP algorithm (Table 2) (66, 67). The results from DSSP also show that the structures on average contain high populations of β-turns (defined H-bond) and bends (no defined H-bond) along with the more dominant unstructured (random coil) conformation. β-turns and bends represent greater than 50% of the secondary structure with unstructured regions accounting for 30–40% in both 15-residue and 120-residues structures. Essentially no β-sheet structure is detected in any of the low-energy CS-ROSETTA structures with a slightly higher helical content observed in the 120-residue structures but, still accounting for <5% of the total conformation. Although this is not a complete secondary structure assessment of the MaSp proteins, the high occurrence of this 15-residue repeat (SI Appendix, Fig. S25) in MaSp1 and the substantial β-turn/bend fraction observed in the NMR-determined structural ensembles, one can easily envision that this could lead to the tubular shaped tertiary structure from the CG-MD simulations (Fig. 1).

**Table 2.** Secondary structure quantification for the NMR-determined CS-ROSETTA ensemble structures.

Secondary structure	15-residue	120-residue
α-Helix	4.0	3.8
β-Bridge	0.0	3.3
β-Strand	0.0	0.6
3_10_-Helix	2.0	5.1
π-Helix	0.0	0.0
β-Turn	22.0	27.9
Bend	30.7	25.2
PP-helix	0.0	1.0
Unstructured	41.3	33.1

The top 10 lowest energy 15- and 120-residue structures were analyzed with DSSP and secondary structure quantification is expressed as a percent with the average reported (see Methods).

Additional atomistic MD simulations were conducted to further support the NMR ensemble structures determined with CS-ROSETTA and CG-MD simulations. Atomistic simulations were conducted with two different force fields that have proven to be effective for IDPs: CHARMM36m (68) and CHARMMIDPSFF (69). Since the CG-M2 simulations indicated ∼400 residues were required for tubular emergence, we also created a 474-residue CHARMM36m atomistic model starting from early CG-M2 timestamps (10, 15 and 25 ns). Average structures from atomistic MD simulations of short (15–100 residue) and long (474 residue) chains are shown in SI Appendix, Figs. S30 and S34; corresponding contact maps for short chains are in SI Appendix, Fig. S31. The DSSP secondary structure analysis of the atomistic simulations is displayed in SI Appendix Table S6. The results of these simulations strongly agree with the NMR structures (Table 2) in terms of secondary structure quantification where the most common secondary structure is the random coil (unstructured) followed by β-turns and bends being the next most common conformation with very low β-strand content observed. We find better agreement with the CHARMMIDPSFF force field simulations particularly for β-turn quantity. In addition, the atomistic simulations have a tendency to over-determine the helical content with some considerable PP II helix observed (5–15%). The biases of these types of MD simulation towards helical structures have been discussed in the literature (69). Nonetheless, strong agreement is observed between the NMR-determined lowest energy structural ensembles and the atomistic simulations in terms of secondary structure where unstructured regions dominate together with β-turns and bends. β-Strand structure is extremely low as are α-helical structures particularly when one considers that these force fields are known to bias towards helices. It is also interesting to note that the secondary structure results for the CHARMMIDPSFF 100-residue simulation are similar to those for the 15- and 120-residue repeat NMR structures (Table 2). This 100-residue sequence appears seven times in the MaSp1 sequence and is completely continuous where the 15-residue repeat, although it occurs often (39 times), is not continuous making the longer 120-residue NMR structures biased towards this 15-residue poly(Ala)-rich domain.

The structures determined by CS-ROSETTA and MD are relatively compact, and the NMR data for Ma silk proteins in native dope resemble IDP's with rapid sub-nanosecond dynamics as revealed by ^15^N NMR relaxation measurements (25). To assess whether these compact structures reproduce the observed dynamics, we calculated ¹⁵N R₁, R₂, and heteronuclear NOE values from 100- and 474-residue atomistic MD trajectories using SpinRelax (70) (SI Appendix, Figs. S32–S37; Table S7). In the 100-residue simulations using CHARMM36 m, R₂ and R₁ values averaged 5.0/1.1 s⁻¹ (800 MHz) and 4.1/1.3 s⁻¹ (500 MHz), with corresponding NOEs of –0.05 (500 MHz) and 0.25 (800 MHz), showing good agreement with experimental trends. Simulations using CHARMM36IDPSFF, which yielded structures more similar in secondary structure (SI Appendix, Table S6) content to CS-ROSETTA models (Table 2), produced slightly higher R₂ values (6.7/1.1 at 800 MHz; 5.4/1.4 at 500 MHz) and more positive NOEs (0.14/0.38), consistent with modestly increased backbone rigidity. These NOE values closely matched experimental values of −0.56/0.34 (500/800 MHz), particularly at 800 MHz, further supporting a dynamic yet partially compact ensemble.

In the 474-residue simulations initialized from CG-M2-derived structures, NOE values remained within the experimental range. Importantly, NOEs increased with simulation starting timestamps—from 0.03/0.39 (500/800 MHz) at 10 ns to 0.33/0.52 at 25 ns—indicating progressive local ordering and reduced backbone flexibility. Among these, only the trajectory initialized from the 10 ns CG-M2 structure retained the anisotropic, tubular morphology (R_g_/R_h_ ∼ 0.91) observed in full-length CG models (SI Appendix Table S2, Fig. S34); later timepoints produced more compact, spherical structures (R_g_/R_h_ ∼ 0.8). R₂ values rose significantly across this series (e.g. from 13.2 to 19.2 s⁻¹ at 800 MHz), consistent with increasing rigidity and decreased internal motion. The 10 ns trajectory, which best matched experimental NOE values, likely reflects a compact but flexible intermediate. However, R₂ values for all trajectories remained elevated compared to experiment—an expected consequence of MD simulations, which do not capture the μs–ms motions that contribute to transverse relaxation in real proteins, especially IDPs, via conformational exchange. These slow fluctuations, which are averaged out in experimental R₂ measurements, are not sampled in MD, leading to systematic overestimation. Conversely, R₁ values are consistently underestimated, as the truncated models do not account for global tumbling and slower collective motions of the full-length ∼300 kDa protein. Among the three parameters, only the heteronuclear NOE shows consistent agreement across force fields and trajectory lengths, particularly at 800 MHz, highlighting that MD accurately captures fast, local dynamics even in larger constructs. Taken together, these data suggest that while the simulated structures are compact, they remain highly dynamic and disordered, with low β-sheet and α-helical content. The strong agreement in NOE supports a model where early-stage tubular assemblies retain substantial internal flexibility, consistent with their proposed role as metastable, preassembly intermediates in the silk spinning process.

The CS-ROSETTA and atomistic MD structures provide critical insight into the emergence of tubular tertiary structure observed in the M2 MD simulations (Fig. 1). The high population of β-turns and bends—comprising over 50% of the secondary structure in the NMR-derived ensemble—helps explain how local disorder can still support compact, anisotropic folding. Notably, β-strand content is essentially absent, and α-helical structure is minimal, with unstructured regions dominating. NMR relaxation parameters calculated from atomistic MD trajectories show strong agreement with experimental values for both 100-residue sequences and a 474-residue simulation initialized from the 10 ns M2 snapshot, which retained a loose tubular geometry. These results demonstrate that although the structures are compact, they maintain internal flexibility consistent with IDP-like dynamics. The combination of continuous β-turns, bends, and disordered coil regions supports the formation of tubular conformations while preserving fast local motion. Lastly, while M3 simulations do not form extended tubular morphologies, they consistently generate compact local beads along the chain with highly anisotropic shape factors (R_g_/R_h_ ∼1.1–1.2). This local compaction combined with a high degree of shape anisotropy may similarly reflect the high β-turn and bend content observed in both NMR and atomistic MD, suggesting a common structural tendency that underlies both ordered and disordered ensembles.

## Conclusions

Together, our multiscale simulations, SAXS ensemble modeling, and NMR data support the structural and dynamic plausibility of tubular conformers in MaSp1 and MaSp2 proteins in spider silk dope. These β-turn- and bend-rich architectures promote compaction while preserving local backbone dynamics characteristic of IDPs. Tubule formation is driven by sequence-encoded amphiphilicity: hydrophobic poly(Ala) segments preferentially pack into the core, while polar residues such as Tyr and Gln remain solvent-exposed, acting as surfactants that stabilize the tubular morphology. Mutational simulations show that the native block-copolymer sequence is essential for tubule formation, while Ala- or Gly-only chains favor wider, β-sheet-prone spherical structures. Solution NMR chemical shift analysis further supports the presence of partially compact structures. CS-Rosetta ensembles generated from experimental backbone shifts display disordered but compact conformations with frequent β-turns and bends. These compact forms assemble into dimers, which are necessary to reproduce features of the experimental SAXS curve, especially in the 1–10 nm q-range. Although tubular conformers represent a minority (∼11%) of the total ensemble, SAXS fitting confirms that their inclusion is essential. The dominant population, consisting of more extended and disordered MARTINI-3-like structures, contributes broader scattering features. These findings suggest that the tubular architecture represents a metastable, sequence-accessible substate that coexists with disordered forms and may serve a distinct role in early assembly. More broadly, they highlight sequence-encoded shape anisotropy as a mechanism by which IDPs can preorganize for self-assembly—relevant not only to silk, but also to biomolecular condensates, fibrils, and synthetic protein materials.

## Supplementary Material

pgaf378_Supplementary_Data

## Data Availability

All data supporting the findings in this study are available in the manuscript and Supporting Information. Models and trajectories have been deposited in Zenodo under DOI: 10.5281/zenodo.16622927, and codes are available at DOI: 10.5281/zenodo.16622909 [PDBSTAPLER] and DOI: 10.5281/zenodo.16622915 [Vesiform].
