# Supplementary material for: Sequence-encoded tubular architectures in disordered spider silk proteins revealed by multiscale simulations and NMR
Source: PNAS Nexus. 2025 Dec 3;4(12):pgaf378. doi: 10.1093/pnasnexus/pgaf378 (PMC12696365; doi:10.1093/pnasnexus/pgaf378)
Supplement: pgaf378_Supplementary_Data [file pgaf378_supplementary_data.pdf]

Supplementary Material for

Sequence-Encoded Tubular Architectures in Disordered Spider Silk Proteins Revealed by Multiscale Simulations and NMR

Christopher J. Forman[a,b,c], David Onofrei[d], Dillan Stengel[d], Julian E. Aldana[d], Christopher Paolini[e], Nathan C. Gianneschi[a,b,c], Gregory P. Holland[d,*]

[a]Department of Chemistry, Northwestern University, Evanston, IL 60208; [b]Department of Materials Science and Engineering, Northwestern University, Evanston, IL 60208; [c]Department of Biomedical Engineering, Northwestern University, Evanston, IL 60208; [d]Department of Chemistry and Biochemistry, San Diego State University (SDSU), San Diego, CA 92182; [e]Department of Electrical and Computer Engineering, San Diego State University (SDSU), San Diego, CA 92182

*Gregory P. Holland

Email: gholland@sdsu.edu

Supplementary Material Includes:

Materials and Methods, Figures S1-S39, and Tables S1-S7.

Materials and Methods

Coarse Grain (CG) Molecular Dynamics (MD) Simulations. Initial random coil structures were created using Profasi or Vesiform[DOI: 10.5281/zenodo.16622915] for Western Black Widow (*L. hesperus)* MaSp1 and MaSp2 proteins (1). Profasi (2) was used with random values for the φ and ψ angles and included the full repetitive core sequence (without N- and C-termini). The N- (PDB Code: 2N3E) and C-terminal (PDB Code: 2KHM) domains are known to form α-helical bundle structures (3, 4) and these were added to the random coils after simulation for completeness using PDBSTAPLER[DOI: 10.5281/zenodo.16622909]. The initial random coil structure prepared using Profasi or Vesiform were passed to the MARTINIZE script to generate the input files for GROMACS MD. Six MaSp1 and four MaSp2 unique random coil structures were attempted this way yielding similar results for MaSp1 and 2, respectively. The coarse-grained protein is solvated using MARTINI CG water and placed in a box with periodic boundary conditions. The system is then charge balanced through the addition of the necessary ions to the solvent, equilibrated for temperature and pressure before being run with the default Verlet scheme for managing non-bonded cutoffs, with a verlet-buffer-tolerance of 0.005, until the radius of gyration (R_g) converges (~1 μs). Simulations were conducted with an Exxact Tensor TWS-289061-GRO (GROMACS Optimized System) – 2x E5-2640 v4, 64GB DDR4, 2x GTX 1080 Ti, CentOS 7 and took approximately one month to complete. Once the simulations were complete, specific frames were extracted and back converted into atomistic models using the CHARMM36 force field to minimize yield pdb files of all structures suitable for analysis. A seventh MaSp1 trajectory was generated that was constructed using a slightly different primary sequence (1) for which a starting backbone conformation was generated using the Vesiform[DOI: 10.5281/zenodo.16622915] software. The φ and ψ angles were placed in a β-sheet conformation and the overall backbone was wound around a large diameter helical space curve. The side chains were added using tleap from Ambertools and the structure was relaxed prior to coarse graining with Martinize script (v 2.6). The remaining coarse grain procedure was subsequently identical to the other six MaSp1 simulations. The eighth simulation of the core 2880 residues was created using the first scheme. All the simulation results and videos can be found in the online database [10.5281/zenodo.16622927].

The Martini-3 trajectory was generated in a similar way using Profasi to generate the initial structure and the MARTINIZE2 script to perform the coarse grain procedure before solvating and equilibrating as usual.

Alphafold. A FASTA sequence file was supplied to AlphaFold2 and run on A100 GPUS using the Quest computer facility at NU. It was found that longer proteins could not be folded within the 48-hour time limit available for simulation and so a set of fragments of the full proteins of gradually increasing length were employed. 1600 residues seemed to be the limit that was possible given resource constraints. We present these results along with their figures of merit.

SAXS Analysis. From two single M2 and M3 MD trajectories, conformations were taken at 10 ns intervals from 0 to 200 ns. Conformations of 8 fully formed tubules were taken from other M2 MD simulations at 1000 ns times. Further longer time scale M3 conformations at 100 ns intervals from 300 ns to 1000 ns were collected. This yielded 29 conformations for M2 monomers and 29 conformations for M3 monomers from 0 to 1000 ns simulation times. These monomers were combinatorially joined into dimers to create pools of 841 M2-M2 dimers, 841 M3-M3 dimers and 841 M2-M3 dimers. PDB files of all conformations were presented to the CRYSOL algorithm which generated theoretical SAXS curves using a solvent density of 0.33440 e$Å^{-3}$, 1275 calculated data points with a maximum scattering angle of 1 $Å^{-1}$ and 20 spherical harmonics. Explicit hydrogens were accounted for and the shell type was directional. The contrast with the solvent shell was 3E-2 e$Å^{-3}$. The pools of dimers were added to the monomers resulting in three pools of M2 only structures, M3 only structures, and all M2 and M3 and M2-M3 Hybrid structures. The corresponding PDBs and theoretical curves were analysed and collated into a single table using SAXSY, our in-house python script [10.5281/zenodo.16622922] to generate and collate the size information (Rg, Porod volume) and intensity curves necessary to give the GAJOE algorithm the information it needed. The GAJOE algorithm used 1500 generations and subsets of 1-50 conformations to perform 100 cycles of a genetic algorithm to try to recreate the experimental data. The best fit set of conformations from all 100 cycles was selected in each case for comparison with the best fit sets from the other pools. The same SAXSY script

plotted all the results using matplotlib, making use of R_g and I_0 values found from Guinier analysis of the GAJOE fitted SAXS curves, that are necessary for plotting the Kratky plot for each curve.

MA Silk Gland Preparation for NMR. Large Western Black Widows (*L. hesperus*) were fed approximately 100 µl of a saturated solution U-[$^{13}C/^{15}N$]-Ala and U-[$^{13}C/^{15}N$]-Phe to label the silk proteins (MaSp1 and MaSp2) for $^{1}H/^{13}C/^{15}N$ solution NMR experiments. Isotope labeling was done three times a week for two weeks. While feeding, the spiders were forcibly silked to deplete the native, unlabeled spidroin supply and encourage the synthesis of labeled proteins. At the end of two weeks the spiders were dissected and the MA glands were extracted in deionized water. For studies on native silk dope, MA glands were rinsed with DI water and transferred directly to a 5 mm Shigemi NMR tube filled with 90:10 H_2O:D_2O.

NMR Experiments and Structure Generation. Solution NMR experiments were collected on a Bruker NEO 800 MHz spectrometer in the UCSD Biomolecular NMR Facility using an X-detect optimized Bruker TXO cryo-probe for X-detect data while, the ^{1}H-detect experiments were collected at 600 MHz with a Bruker RT TXI solution NMR probe in the NMR Facility at SDSU. NMR data was processed with NMRPipe and analyzed with a combination of NMRFAM-Sparky and its successor, Poky (5-7). A combination of traditional ^{1}H-detected and ^{13}C detected solution NMR experiments were used to make sequential assignments for one 15-residue repeat sequence. Despite huge spectral overlap and line broadening, traditional ^{1}H-detect solution NMR pulse sequences HNCACB, CBCAcoNH, ^{15}N-HSQC, and ^{13}C-HSQC experiments were able to resolve 6 residues in the Gly-Gly-X region (8-10). Using an extremely tight matching threshold in NMRFAM-Sparky/Poky a classic backbone walk approach led to a fully assigned sequence GQGGAG. The same approach was able to resolve three other triple-residue repeat: GAA, AAA, and AAG. Using our knowledge of the sequence, we placed the 4 resolved sequences in order of appearance, making the informed assumption that directly adjacent Ala residues within the AAA region would not have markedly different chemical shifts. This allowed us to generate a list of chemical shifts for one fully assigned Gly-Gly-X next to one fully assigned poly(Ala) region of the protein giving us the sequence: **GQGGAGAAAAAAAAG.** Five of the nuclei (Cα, Cβ, NH, ^{15}N, and Hα) for structure ensemble determination in CS-Rosetta were determined in this way. Carbonyl assignments were made using a combination of X-detect CACO and CON experiments for sequential assignments of two-residue pairs (11). These pairs of assignments were then added to the sequences based on their order and appearance in the full MaSp1 sequence. NMR chemical shifts were referenced to DSS.

Isotropic solution NMR chemical shifts for the six backbone nuclei (Cα, Cβ, NH, ^{15}N, Hα, CO) were extracted for the **GQGGAGAAAAAAAAG** motif in *L. hesperus* MaSp1 and used as inputs for φ/ϕ restraints from TALOS-N (12) that were then fed into CS-ROSETTA (13, 14) for low-energy structural ensemble determination of this motif. TALOS-N uses chemical shifts and a combination of neural network analysis and database comparison to find restraints on φ/ϕ angles of individual residues. The output angles are also scored based on how well the prediction is likely to be and how large the expected deviations are. These parameters can then be fed as restraint files into CS-ROSETTA, a set of tools coupled to the ROSETTA *ab initio* structure prediction software. Here the predicted angles and secondary structure are used to select fragments of proteins from the BMRB which most closely resemble the input sequence. From these fragments, Monte Carlo structure generation and relaxation using ROSETTA results in a landscape of predicted structures with various energies. If the resulting top ten lowest energy structures are extremely similar, the results are said to converge, and final structures are probably representative of the true structure. Larger structures were also generated that contained 120 amino acids by linking together the 15-residue repeat 8 times and repeating the chemical shift information. For both the 15-mer and 120-mer, CS-ROSETTA structure calculations converged, indicating high confidence in the structures. Secondary structure was determined from the NMR structures using DSSP. Residue-residue contact maps for the structures were generated utilizing MDAnalysis (15). Contacts were defined with an 8 Å cut-off at residue Cα sites.

Atomistic MD simulations. Atomistic MD simulations were conducted for 15-, 60- and 120-residue structures starting from an initial random coil conformation similar to the MARTINI simulations. These sequences were the same as the ones used for the NMR-determined CS-ROSETTA ensemble structures. An additional 100-residue sequence that appears seven times in the *L. hesperus* MaSp1 sequence and a 474-residue sequence starting from early M2 trajectories timestamps (10, 16 and 25 ns) was also simulated.

The simulations were conducted using two different force fields in GROMACS that have been shown to be effective for IDPs: CHARMM36m (16) and CHARMMIDPSFF (17). The atomistic protein was solvated using explicit waters with the TIP3P forcefield and placed in a box with periodic boundary conditions. Secondary structure was determined from the atomistic simulated structures using DSSP (18). The average secondary structure is reported from 500-1000 ns simulation time for 15- and 60-residue ensemble structures. For the 100-residue structure, simulations were only conducted to 500 ns and secondary structure was analyzed from 250-500 ns. For the 474-residue structure, 200 ns simulation was run and DSSP analysis is from the final trajectories. Residue-residue contact maps for the structures were generated utilizing MDAnalysis (15). Contacts were defined with an 8 Å cut-off at residue Cα sites. NMR relaxation parameters (R_1, R_2, and heteronuclear NOE) were calculated from the 100-residue and 474-residue atomistic MD trajectories using SpinRelax (github.com/zharmad/SpinRelax) (19). Overall rotational diffusion constants (D_{rot}) were estimated with HYDRONMR (20) using the full atomic coordinates from each model. Bond vector time correlation functions (C(t)) were computed for backbone ^{15}N–^{1}H pairs, and relaxation parameters were derived via spectral density mapping. The SpinRelax tool was benchmarked using GB3 CHARMM36m simulations (Figure S38 and S39) with results matching the original manuscript (19). Average values across the spidroin trajectories are summarized for the spidroin sequences in Table S7.

Simulation Analysis. As well as Profasi, in-house software, Vesiform [10.5281/zenodo.16622915], was used to generate starting structures. Vesiform, takes a text sequence of amino acids and creates a prototype $(N\text{-}C\alpha\text{-}C)_n$ backbone that conforms to a specific secondary structure, bond lengths and internal bond angles. Vesiform is a more general package that is designed to create strings of beads that obey arbitrary user specified constraints, such as overall external packing volumes, regions to avoid, and internal bond length, bond angle and dihedral distributions. In this instance, a smooth trajectory is wound around a large cylinder and a string of beads along that trajectory are locally endowed with either α-helix, β-strand, or a mixed character dihedral angles or both, by using the free end of the chain to define a local Frenet frame of a helix and setting the bond lengths, angles and dihedrals of each new particle as we work along the trajectory, satisfying local constraints while avoiding global self-interactions.

The bond lengths, internal angles and omega dihedrals are chosen from very narrow ranges, whereas dihedrals for φ and ψ angles are picked randomly from a wider set of specified ranges, to yield specific secondary structure of the peptide sequence. Wrapping the initial structure around a large cylinder while constraining internal angles is necessary for extremely long sequences like MaSp1 to minimize the overall box size required to solvate the protein in later simulation processes. Also, for proteins stretched out longer than 1000 angstroms, there are often problems in formatting their coordinates in PDB files and importing them into VMD to be rendered. For shorter fragments these considerations are less important.

Vesiform generates only the backbone of the peptide. Once an elongated $(N\text{-}C\alpha\text{-}C)_n$ backbone has been formed with dihedral angles picked from a randomly specified range, a partial PDB file is generated consisting only of the N, Cα and C atoms, alongside residue identity labels derived from the input sequence. The side chain atoms for the partially formed PDB file are then populated using tleap from AmberTools, which completes the structure by fitting standard amino acid residues to the correctly defined backbone positions and generating forcefield parameters for the resulting full protein (Amber FF19SB). The full correctly formatted topology (prmtop) and coordinate files are exported for input into a range of subsequent simulation programs. The initial structures are relaxed using Sander and these can also be used as inputs for GROMACS and Martini. The result is a straightforward way to generate a wide range of starting structures with specific sequences from a text file definition of a protein including sequences and bond angle ranges. This tool is available for public use, but it is not supported and has not had significant alpha or beta public testing.

Tube Axial Trajectory Determination. A range of methods were explored for quantitatively assessing the intuitively obvious tube-like nature of the coarse grain structures. The best method found for revealing the intuitive shape of the tubule structure was to compute the running average for the center of mass of *n* residues at incremental steps of *m* residues. Fig. S2 shows the resulting center of mass trajectory along the protein structure for different values of *n*. When *n* was between 150 to 200 residues the tubular structure

that matches our intuition is revealed. If *n* is too high (>400 residues) then the tube structure itself is smoothed out . For *n*<150, the center of mass trajectory is insufficiently smoothed.

Tube Radius Determination. MaSp1 and MaSp2 structures were divided into sections around 400 residues long and their center of mass line was determined by a running average of 150 residues along each segment. Since these sections are relatively short, a straight line can be fitted to this center line by least squares regression, allowing computation of the shortest distance from each residue in the segment to the centerline. The number of residues in cylinders of varying radii can be counted and summed from the center outwards which reveals a sigmoidal graph that tops out around a radius ~2 nm. Each segment can be overplotted to reveal very similar graphs (Fig. S3). Such an analysis yields a simple way of measuring a nominal tube radius that incorporates positional information from every residue in the entire structure. We quote values for the tube radius that contains 90 percent of the residues of the 400 residue sections. There are edge effects at the end of each segment and so a small number of residues have their radius over estimated. Hence the omission of the top 10% of radius numbers.

Averaged Contour Length. Once a tube axis has been determined a centerline trajectory for the tubules associated with every MaSp1 and MaSp2 structure exists, enabling a contour length to be found, simply by summing the length of the sections of the trajectory.

Max Euclidean Distance. The distance between every amino acid pair is computed and the longest such distance in the protein is noted.

Hydrodynamic Radius (R_h). R_h was computed by submitting the PDB files to an online service using the HullRad algorithm (21).

Radius of Gyration (R_g). R_g was computed using an in-house function contained within the pdbProc (10.5281/zenodo.16622909) utility available from GitHub, that was validated against Pymol R_g output. pdbProc is unsupported and has not had significant Alpha or Beta public testing. This is a command line utility for manipulating PDB files in a variety of ways. R_g can also be computed for a structure with the ATSAS software suite by fitting a Guinier curve to theoretical SAXS data computed using the CRYSOL package.

Surface area analysis. Surface area analysis of PDB files from the simulations was conducted using the rolling ball method via the online algorithm GetArea (22). The radius of the rolling ball was 1.4 Å. A residue is determined to be inside or outside based on the ratio of the solvent exposed surface of the side-chain of each amino acid with the "random coil" value per residue—the average across 30 random conformations of the solvent-accessible surface area of X in the tripeptide Gly-X-Gly. When the ratio exceeds 50% it is outside, and if it is less than 20% it is considered buried.

These values were averaged across 8 or 4 conformations for MaSp1 and MaSp2, respectively, to give a typical value and a standard deviation for the contribution to the overall surface area of each residue type and presented in Table 2 in the main paper. The ratio of average total area for each residue type to the number of residues of each type (the column labelled A/N) gives some indication as to the spatial distribution of each type of residue with respect to the surface or the bulk of the spidroin conformations. A second indicator is the ratio of outside to inside residues. It must be borne in mind that as the radius of the tube geometry increases, the outer surface area also increases, so the behavior of these ratios depends on the overall geometry of the protein. To get some idea of relative function for a given structure, these values can be compared with the values associated with all the residues in the protein to determine if a residue type is over- or under-represented on the surface of the protein. Given the extended nature of the conformations arising from the M3 simulations the required lattice exceeded the capacity of the online system to perform the rolling ball method. Therefore, the surface area analysis could only be conducted on 55% of each conformation.

The Simple Cylindrical Model is a quasi-continuous model that computes the successive volume of concentric cylindrical shells and calculates how many small spheres, representing residues, would be needed to exactly match the volume of each shell. A nominal sphere radius of 0.4 nm was used to represent

each residue. Such a nominal radius was computed from the fact that a globular protein with ~100 amino acids (e.g. cytochrome b562) is 3-5 nm across, which can be crudely modelled as a sphere with radius 4 nm, enabling the volume per residue to be computed and hence a *nominal* radius for a residue. The thickness of concentric shells in this model was taken at 0.4 nm intervals to enable an estimate of the ratio of bulk to surface residues that one would roughly expect for a cylindrical protein structure. We ignore end effects of the cylinder for simplicity, and we assume that each sphere would pack someway into the layer below, so the layer thickness is taken to be the radius of the sphere, rather than the diameter. The main axial length of the cylinder is taken to be 50 nm which is the average contour length of the MaSp1 center of mass trajectory. Choosing geometric parameters for the cylinder that match our tubule analysis and the number of residues that match our sequence, results in a surface area to bulk ratio that matches what we see in our solvent accessible surface area analysis.

Dimerization of Monomers: The PDBProc software [10.5281/zenodo.16622909] contains a stapling algorithm (PDBStapler) that uses interim protein structures to align two different protein structures closely together. One target protein structure is held fixed, and part of the C-C-N backbone of the staple is aligned with similar sized region of the static target using the MinPermDist algorithm (23). Once in position the staple is fixed and a second part of the staple is used as a reference to align with a sub-region of the second target protein. Once the second alignment is achieved, the staple is discarded leaving two target proteins in a spatial relationship that is defined by the staple. This process can be repeated to concatenate multiple proteins. Sub-groups of the combined structure can be rotated around specific dihedrals or bonds to relieve steric clashes. In this work, the experimentally derived structure for the N terminal dimer 6R9D was used as a staple to link together the N termini (2N3E) of monomeric structures.

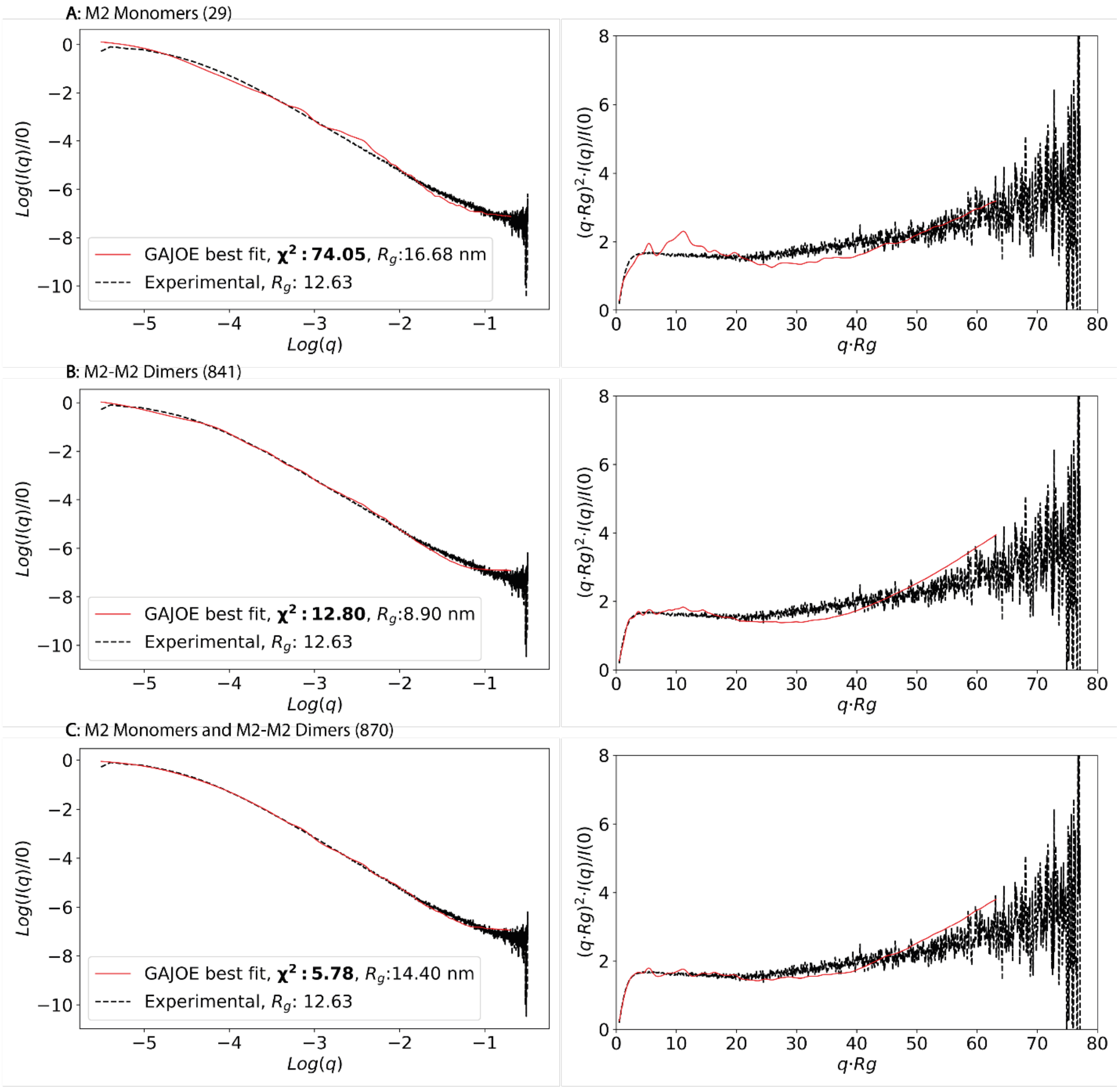

Figure S1, A-C: Eight pools of structures were curated and supplied to GAJOE to fit structures to experimental data. Here we plot the fitted curves against the experimental data, as Log-Log plots (left) and dimensionless Kratky plots (right). A, B, C: Martini 2 Models, D, E, F: Martini 3 models, G: Hybrid M2-M3 models, H: All monomers and dimers.

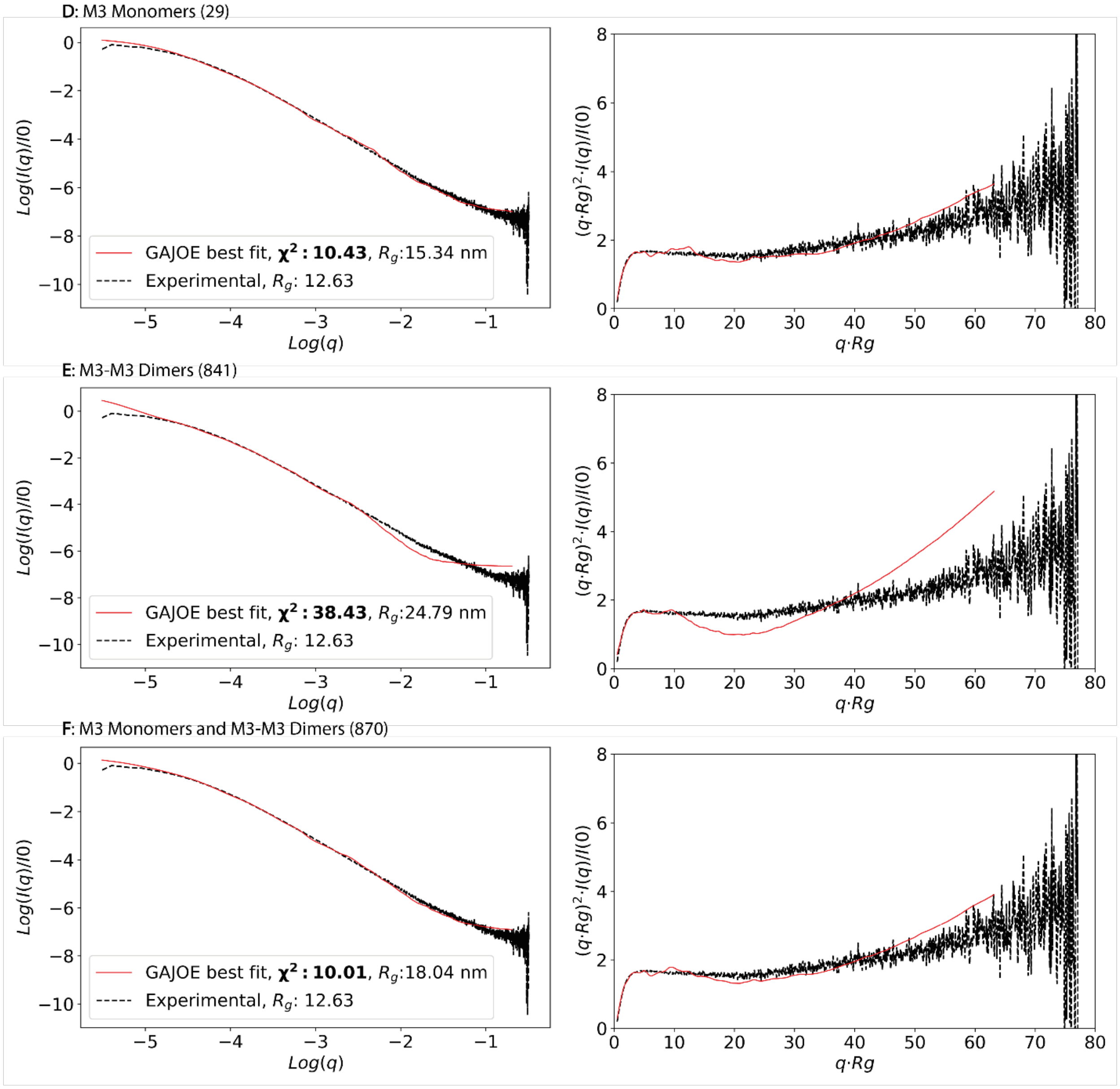

Figure S1, E-F: Eight pools of structures were curated and supplied to GAJOE to fit structures to experimental data. Here we plot the fitted curves against the experimental data, as Log-Log plots (left) and dimensionless Kratky plots (right). A, B, C: Martini 2 Models, D, E, F: Martini 3 models, G: Hybrid M2-M3 models, H: All monomers and dimers

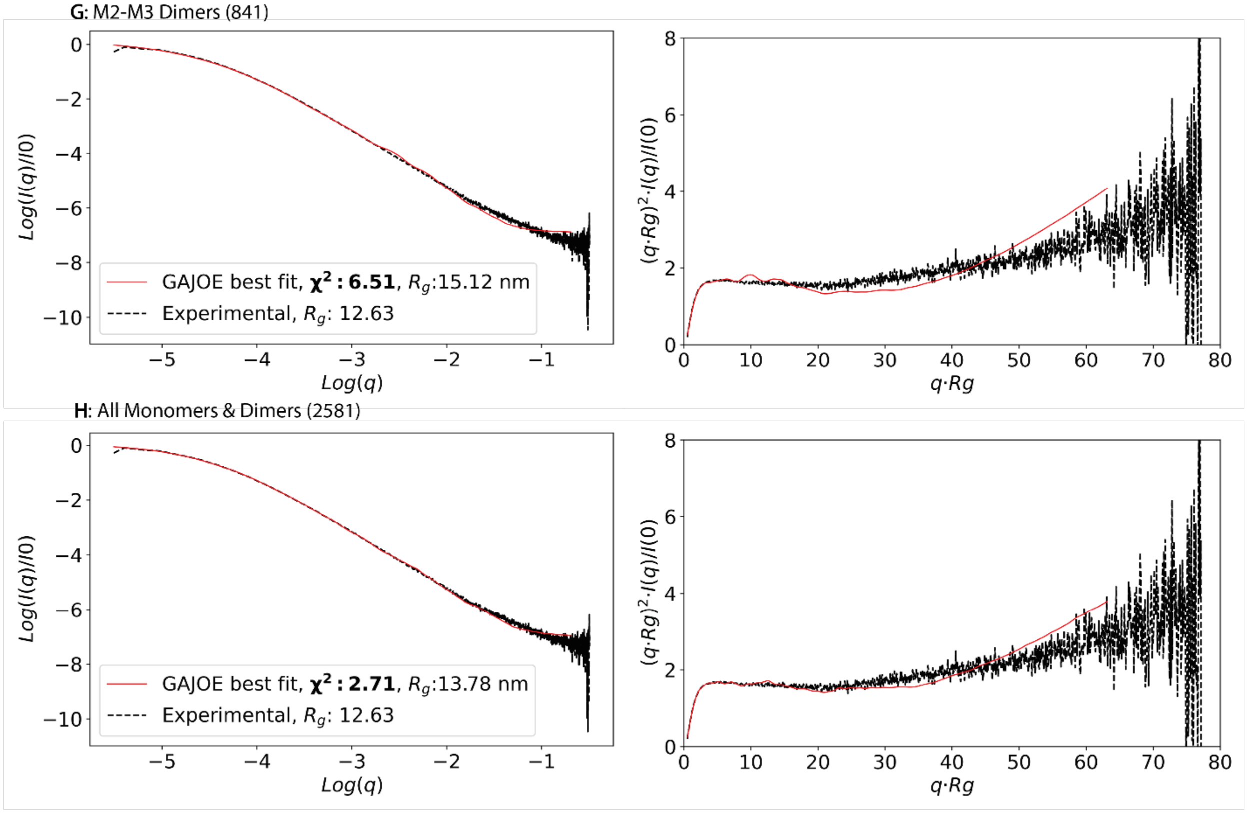

Figure S1 G,H: Eight pools of structures were curated and supplied to GAJOE to fit structures to experimental data. Here we plot the fitted curves against the experimental data, as Log-Log plots (left) and dimensionless Kratky plots (right). A, B, C: Martini 2 Models, D, E, F: Martini 3 models, G: Hybrid M2-M3 models, H: All monomers and dimers.

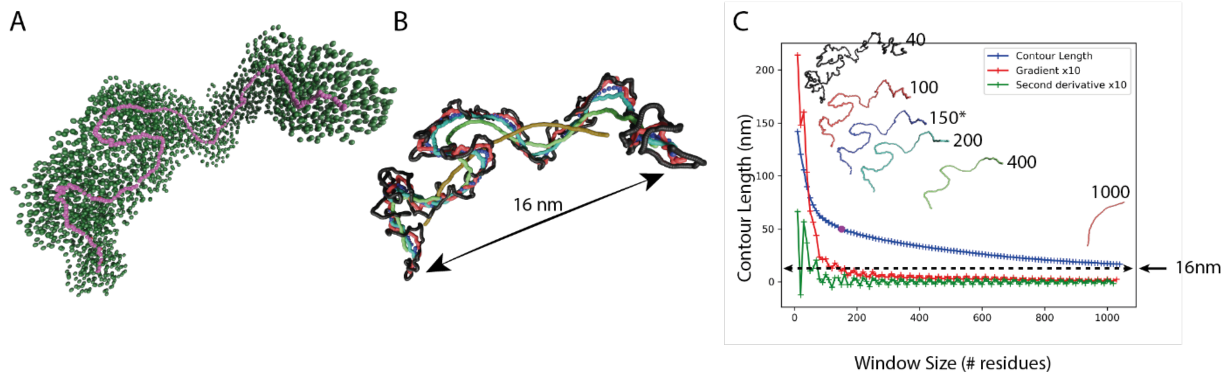

Figure S2. (A) The green spheres are the positions of the α-Carbons in one of the spidroin compact tubule structures. The distribution of residues along the tubule roughly follows the sequence order of the protein so performing a running average of all the α-carbon positions in subsets of n residues in sequence order generates a local center of mass (pink spheres) that follows the intuitive shape of the tubule. (B) A running average of the α-carbon positions with different window sizes smooths out different amounts of the detailed backbone conformation. (C) The contour length of the resulting backbone can be plotted against window size showing a clear knee of the curve around a window size of 100 residues, which is clearer in the first derivative (red line) and second derivative (green line). The position of this discontinuity is likely related to the simulation run time, as disparate parts of the protein would not have had sufficient time to interact. If the window size is increased, the contour length tends towards the end-to-end distance of the protein, and the tubular shape is averaged out. A value of 150 residues was picked as a standard which yields a tubule contour length of 50 nm and suggests a minimum unit size for the simulation time, that is above the discontinuity.

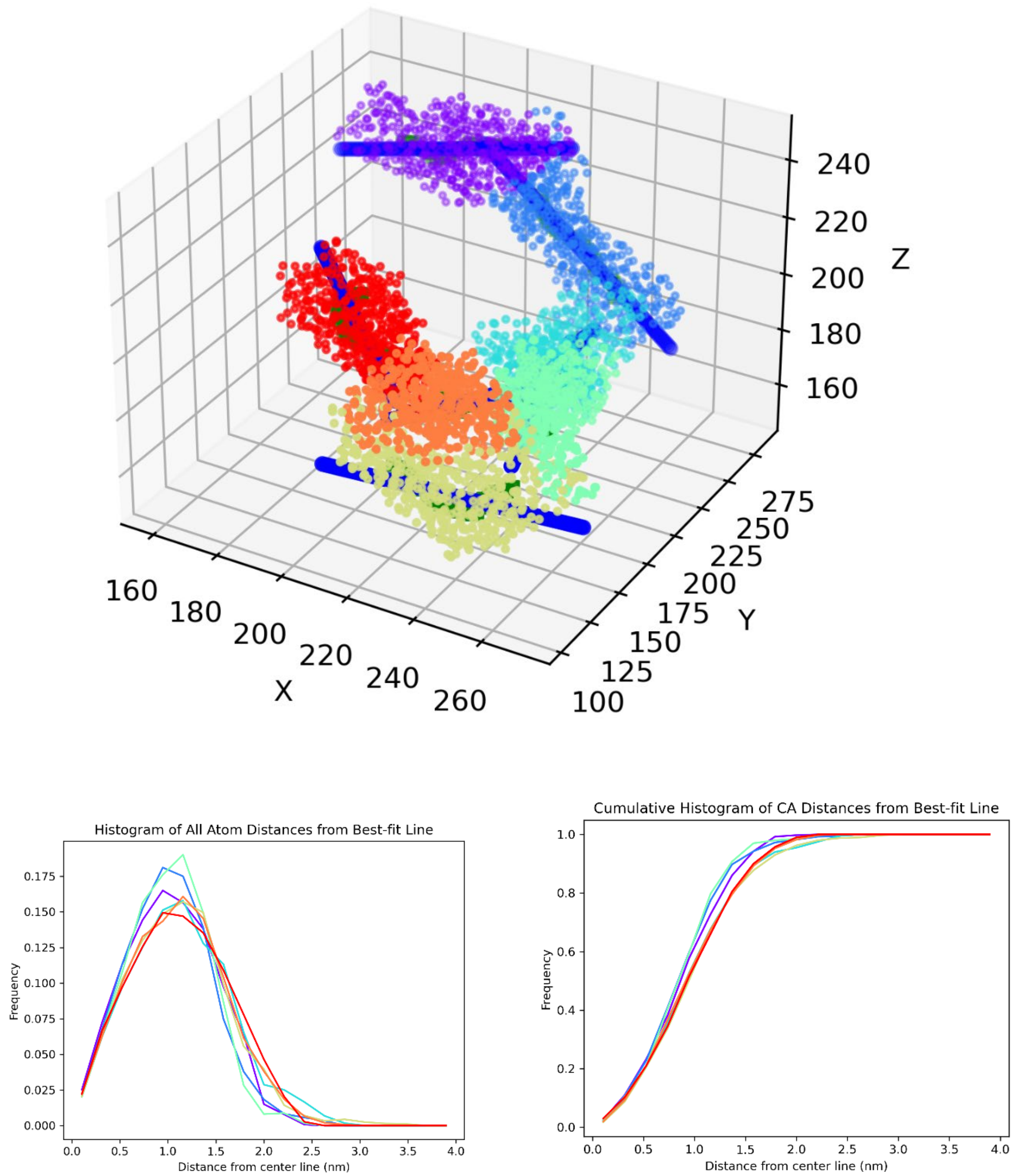

Figure S3. The α-carbon positions of 400 residue sections of the MARTINI model could be fitted with straight lines, yielding a local cylindrical axis, from which distance of residues can be confirmed, and averaged yielding a local tube radius. The results from each section can be overplotted confirming agreement between the segments.

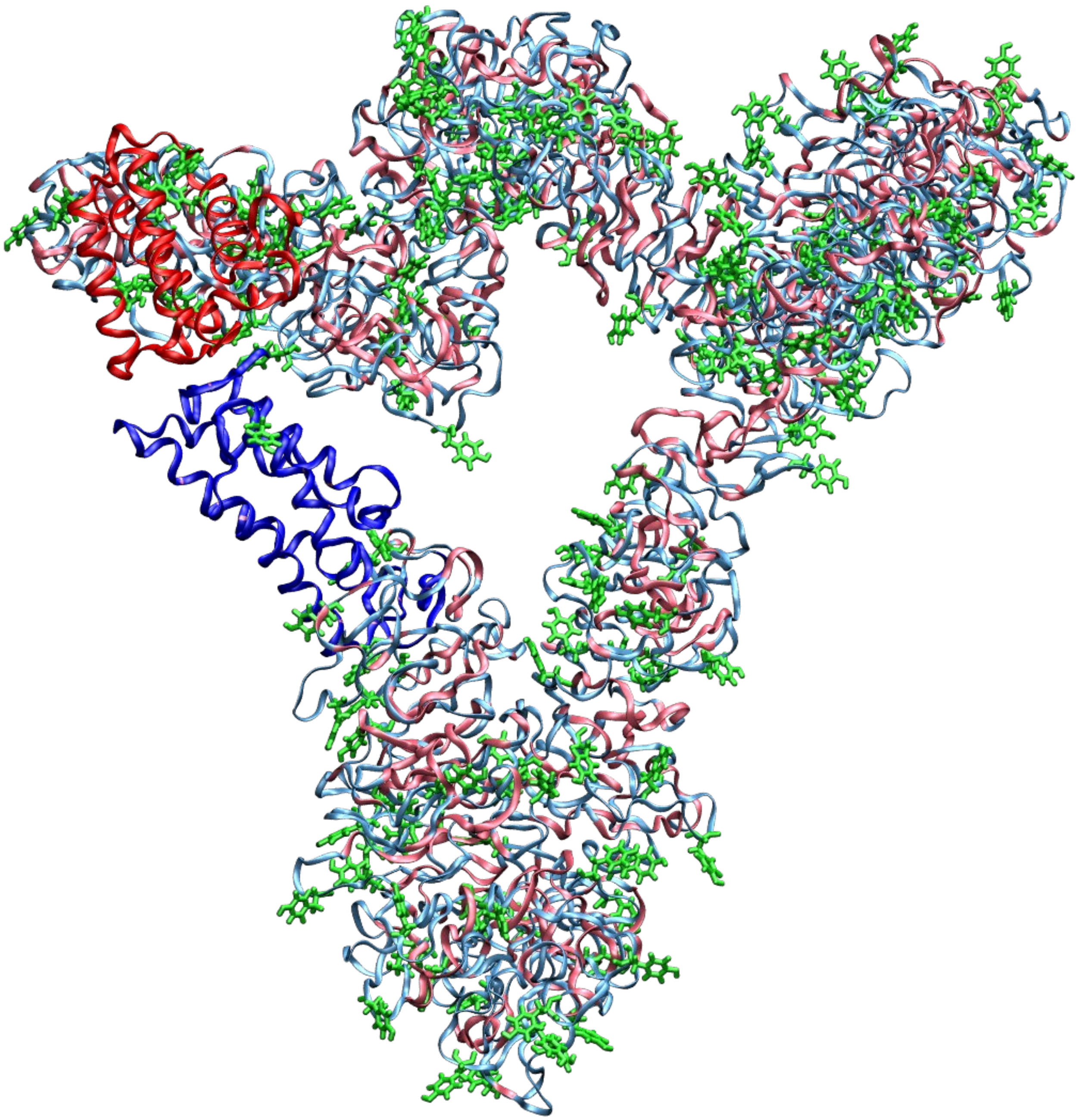

Figure S4. Structure aa_2. Largescale image of the CG-MD model conducted with the MARTINI-2 forcefield in GROMACS for MaSp1 (Main text Fig 1, run 1). The color coding is as follows, green: Tyr, pink: Ala, red: N-terminus, blue: C-terminus, light blue: everything else.

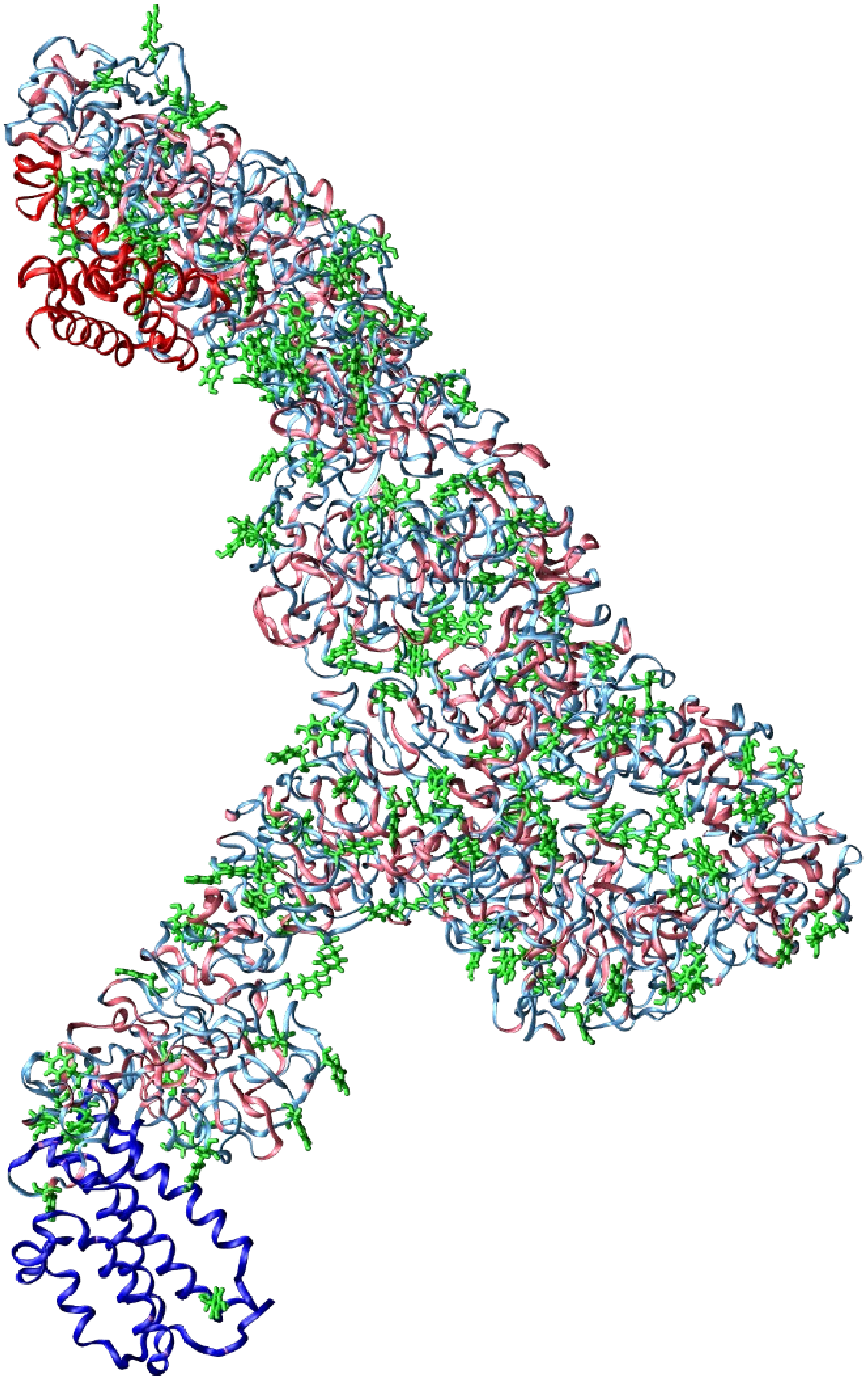

Figure S5. Structure aa_20. Largescale image of the CG-MD model conducted with the MARTINI-2 forcefield in GROMACS for MaSp (Main text, Fig 1, run 2). The color coding is as follows, green: Tyr, pink: Ala, red: N-terminus, blue: C-terminus, light blue: everything else.

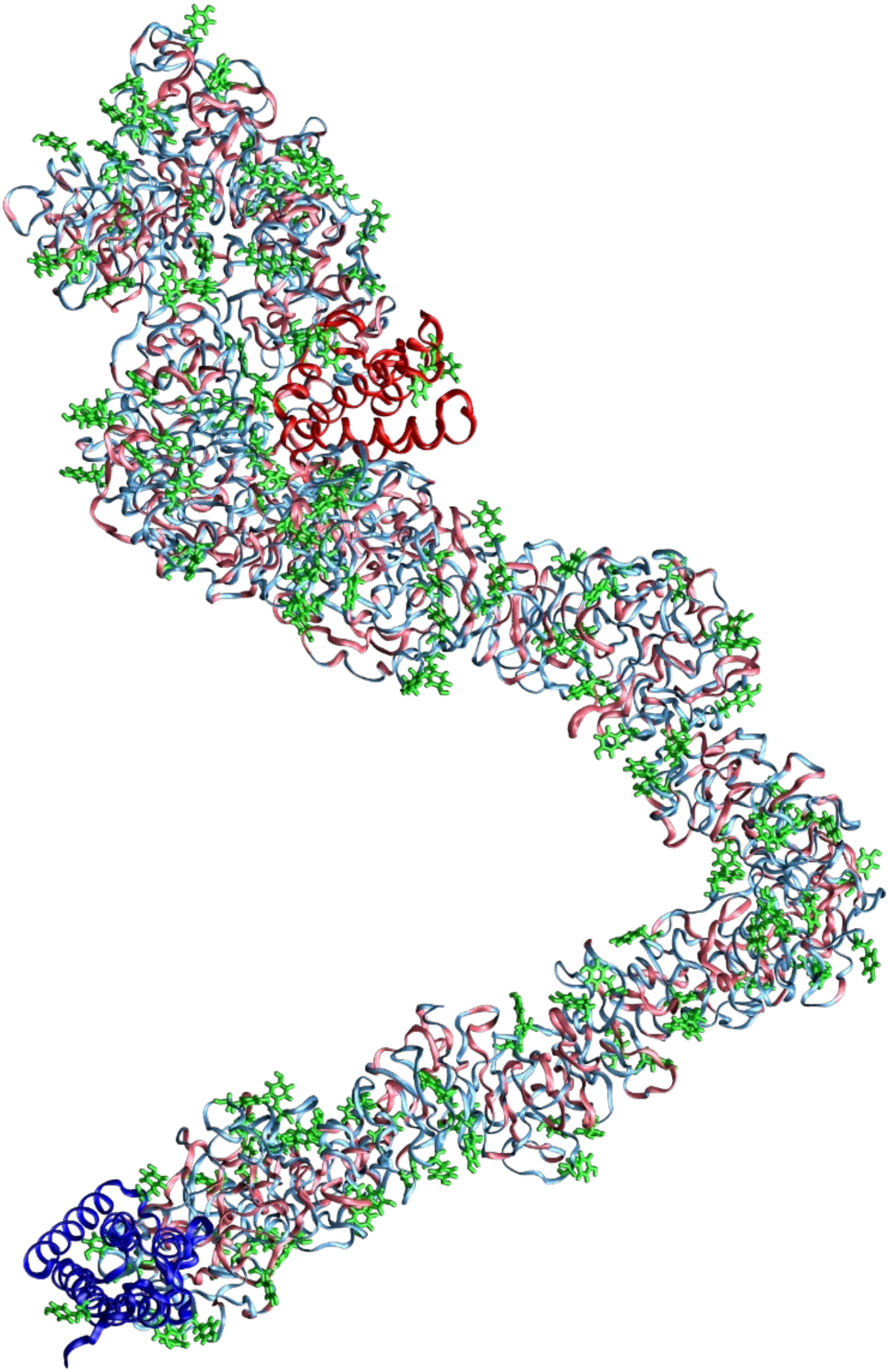

Figure S6. Structure aa_30. Largescale image of the CG-MD model conducted with the MARTINI-2 forcefield in GROMACS for MaSp1 (Main text, Fig 1, run 3). The color coding is as follows, green: Tyr, pink: Ala, red: N-terminus, blue: C-terminus, light blue: everything else.

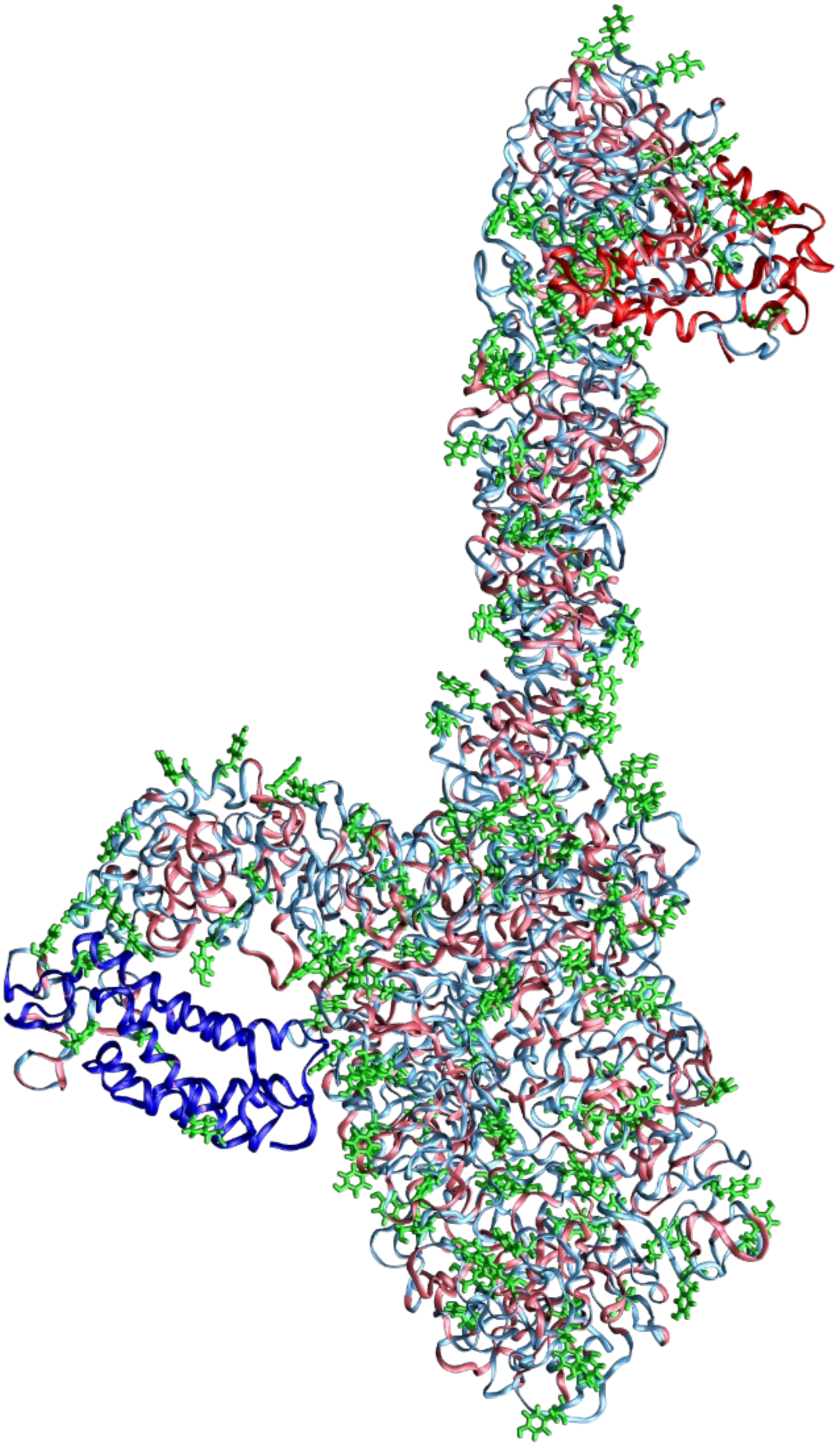

Figure S7. Structure aa_5. Largescale images of the CG-MD models conducted with the MARTINI-2 forcefield in GROMACS for MaSp1 (Run 4). The color coding is as follows, green: Tyr, pink: Ala, red: N-terminus, blue: C-terminus, light blue: everything else.

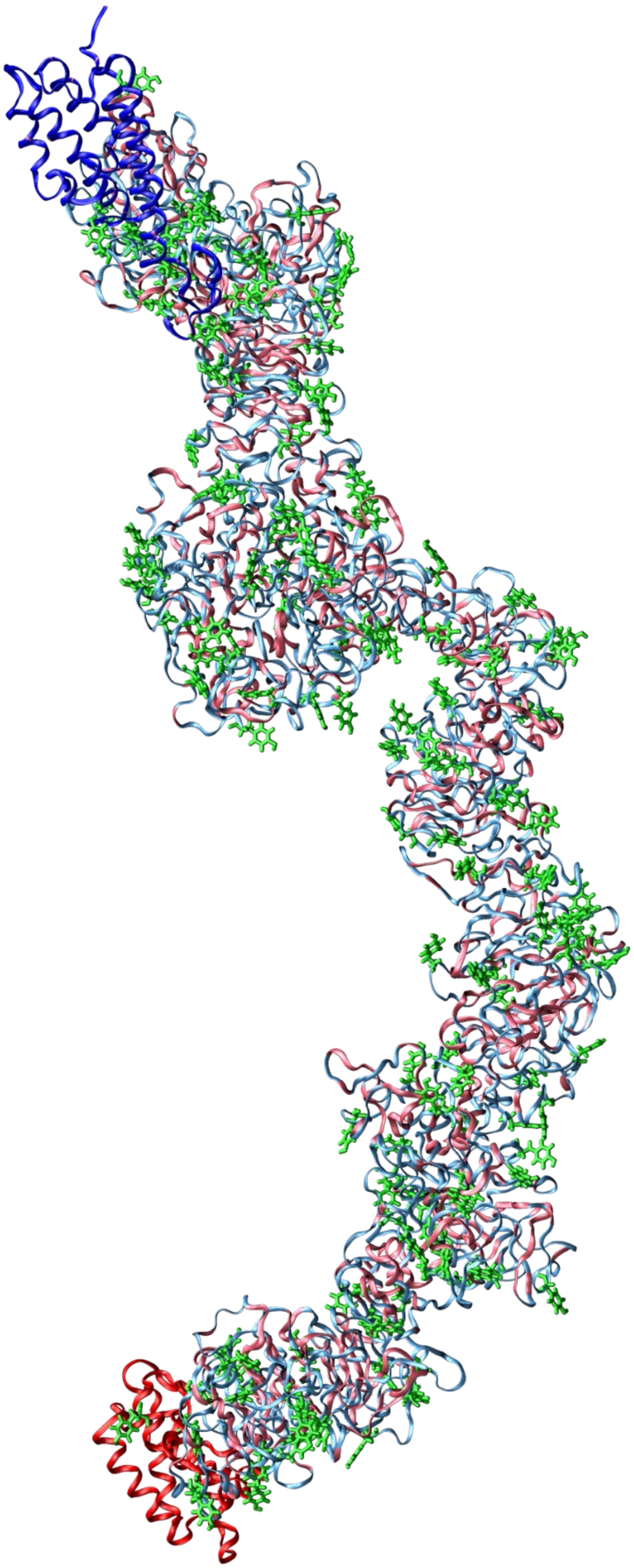

Figure S8. Structure aa_10. Largescale image of the CG-MD model conducted with the MARTINI-2 forcefield in GROMACS for MaSp1 (Run 5). The color coding is as follows, green: Tyr, pink: Ala, red: N-terminus, blue: C-terminus, light blue: everything else.

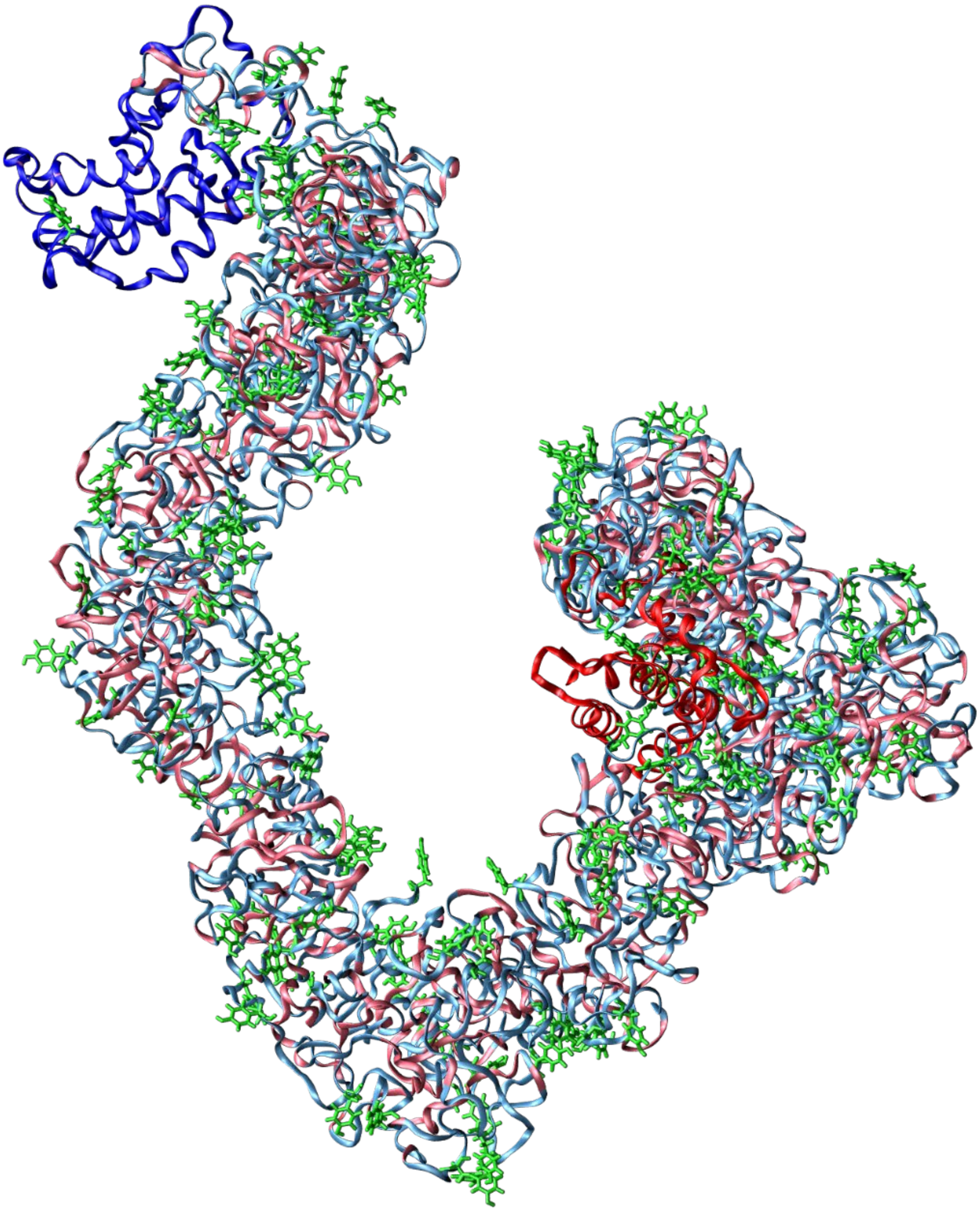

Figure S9. Structure b_1. Largescale image of the CG-MD model conducted with the MARTINI-2 forcefield in GROMACS for MaSp1 (Run 6, Conformation 1). The color coding is as follows, green: Tyr, pink: Ala, red: N-terminus, blue: C-terminus, light blue: everything else.

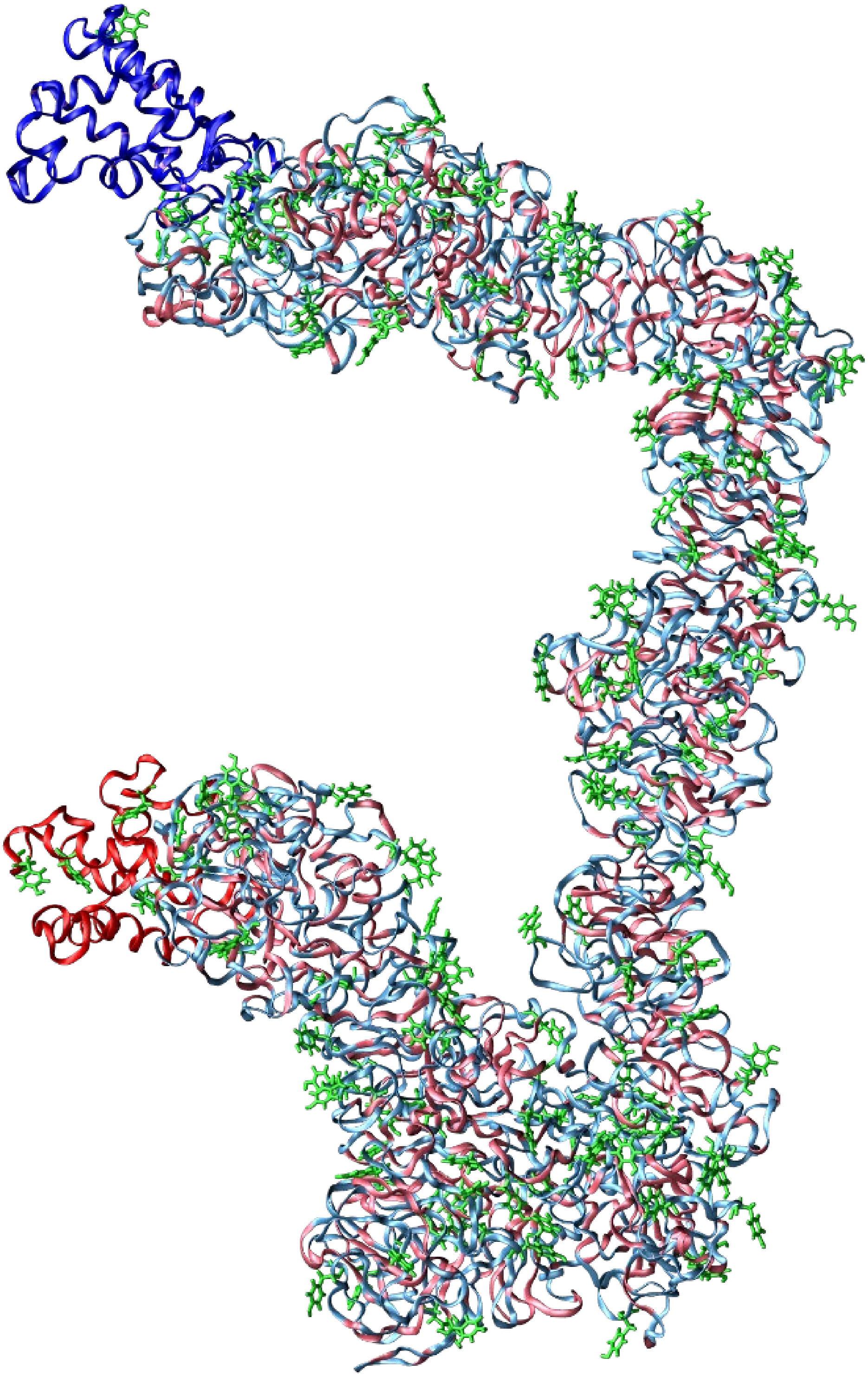

Figure S10. Structure b_2. Largescale image of the CG-MD model conducted with the MARTINI-2 forcefield in GROMACS for MaSp1 (Run 6, Conformation 2). The color coding is as follows, green: Tyr, pink: Ala, red: N-terminus, blue: C-terminus, light blue: everything else.

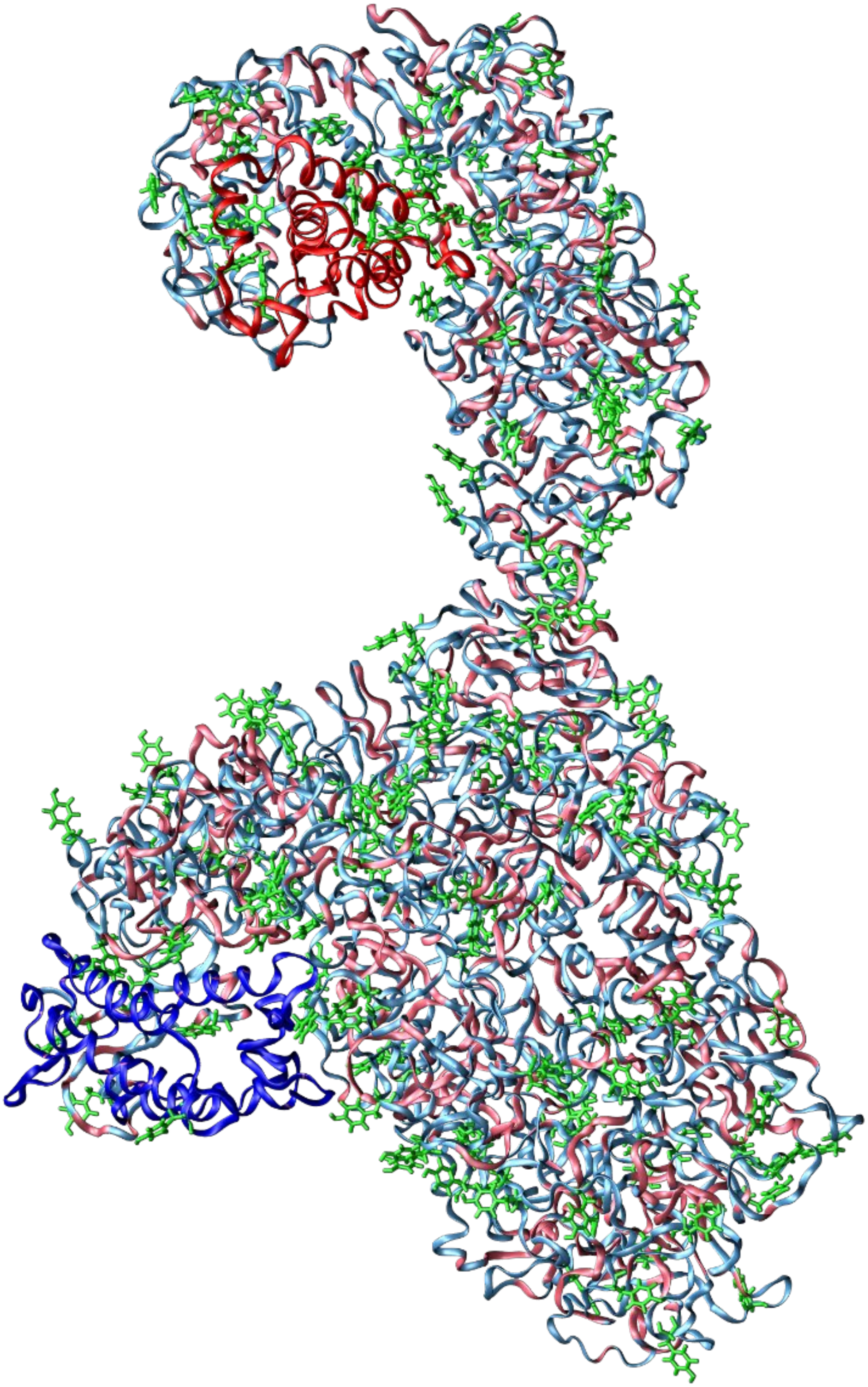

Figure S11. Structure b_3. Largescale image of the CG-MD model conducted with the MARTINI-2 forcefield in GROMACS for MaSp1 (Run 6, Conformation 3). The color coding is as follows, green: Tyr, pink: Ala, red: N-terminus, blue: C-terminus, light blue: everything else.

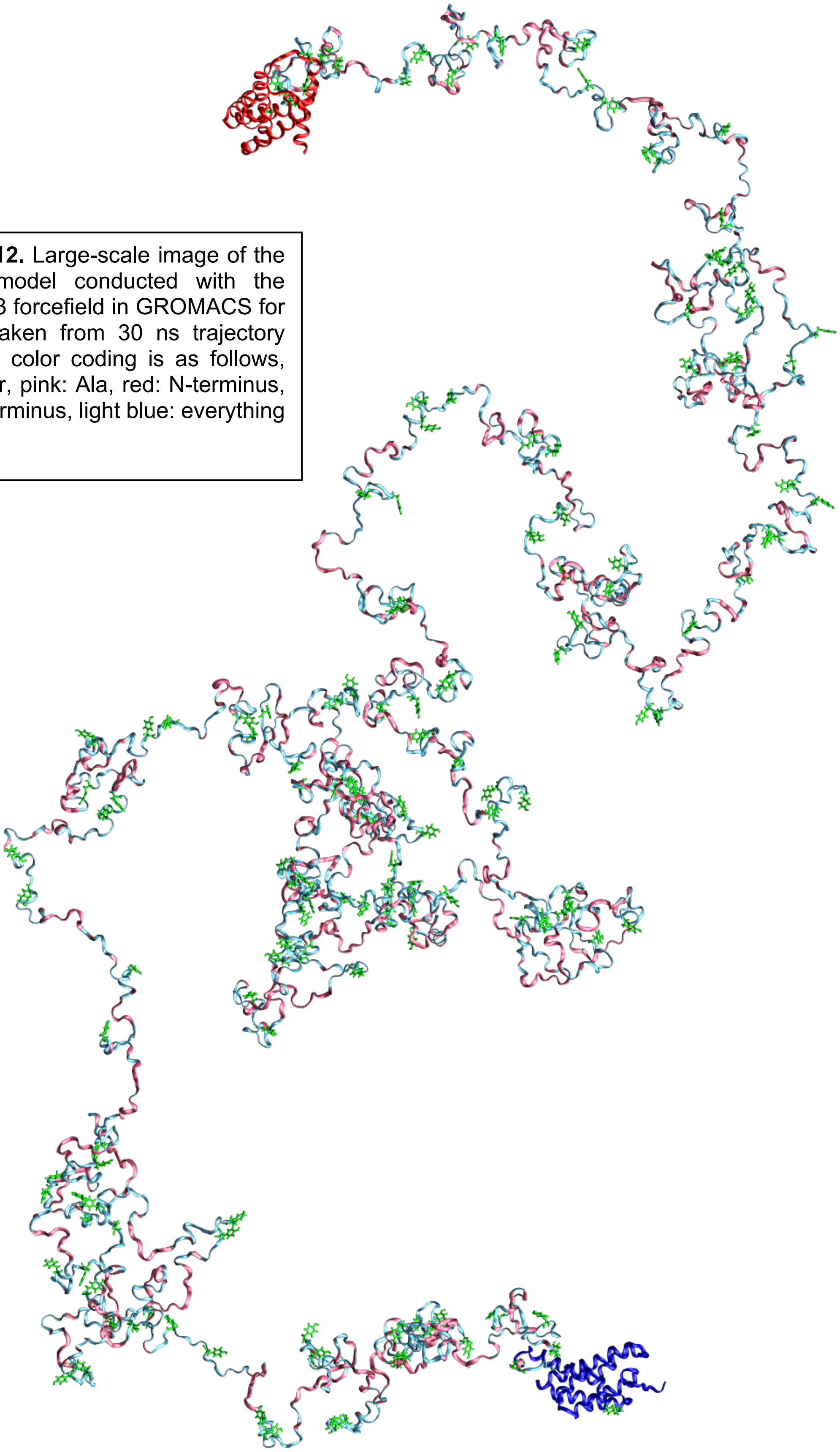

Figure S12. Large-scale image of the CG-MD model conducted with the MARTINI3 forcefield in GROMACS for MaSp1, taken from 30 ns trajectory time. The color coding is as follows, green: Tyr, pink: Ala, red: N-terminus, blue: C-terminus, light blue: everything else.

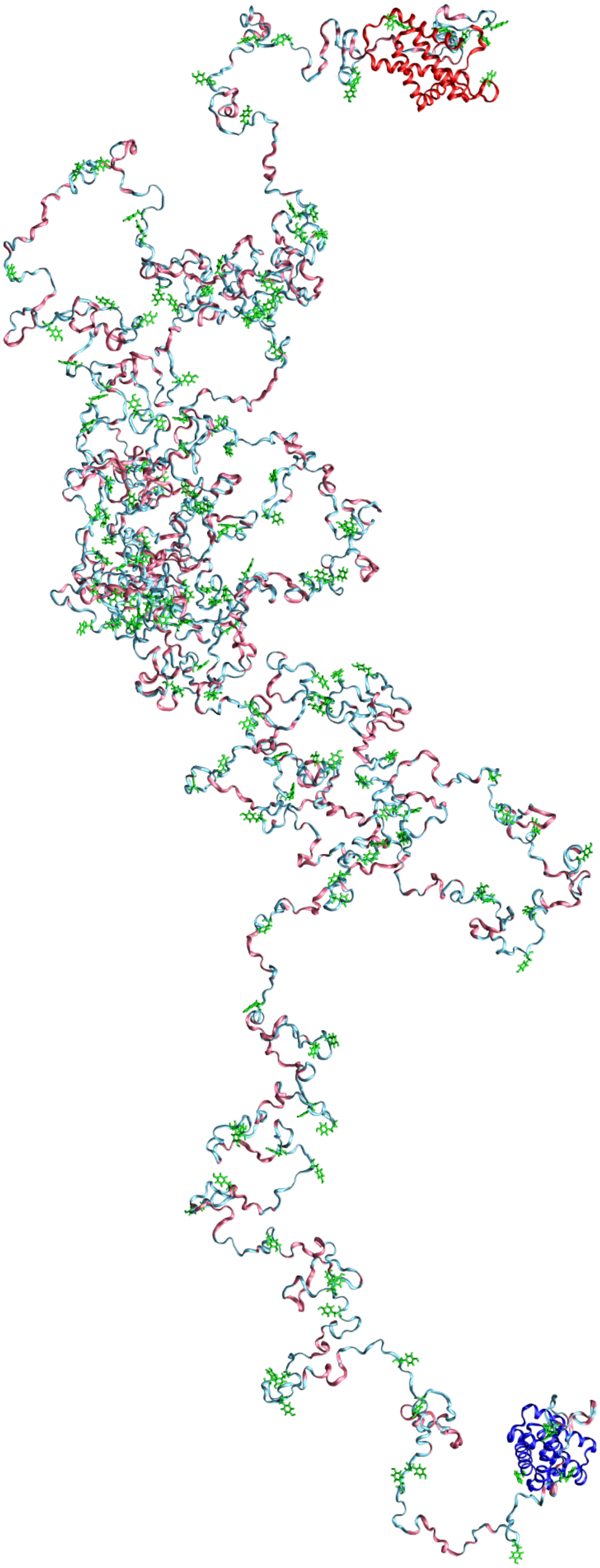

Figure S13. Large-scale image of the CG-MD model conducted with the MARTINI3 forcefield in GROMACS for MaSp1, taken from 200 ns trajectory time. The color coding is as follows, green: Tyr, pink: Ala, red: N-terminus, blue: C-terminus, light blue: everything else.

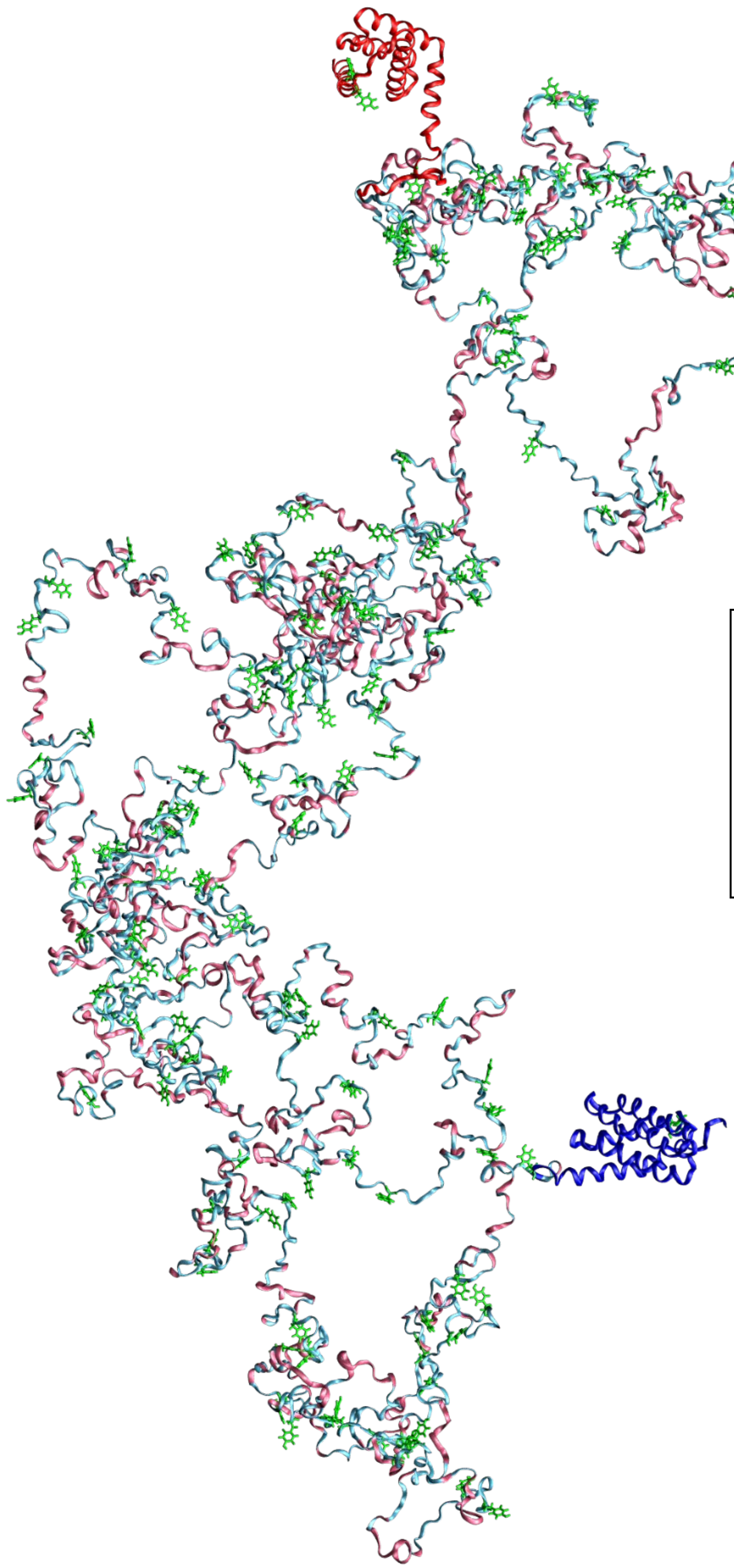

Figure S14. Large-scale image of the CG-MD model conducted with the MARTINI3 forcefield in GROMACS for MaSp1 taken from 400ns trajectory time. The color coding is as follows, green: Tyr, pink: Ala, red: N-terminus, blue: C-terminus, light blue: everything else.

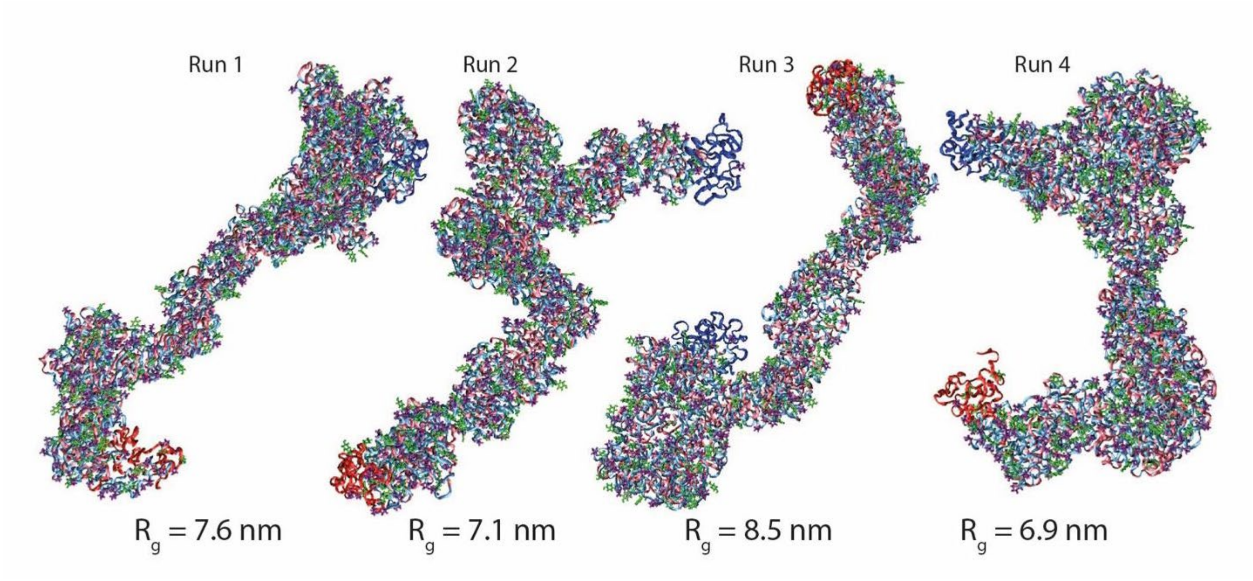

Figure S15. (A) The full MaSp2 amino acid sequence is modeled with a MARTINI-2 coarse grain force field. The starting configuration of the tandem repeat region is a random coil. For each run, a different randomly generated structure was used analogous to the MaSp1 models shown in Fig. 1 of the main paper. After 1000 ns of simulation time, the overall shapes are vastly different, yet possess a consistent distinctive tubular character which is conserved across all the repeated simulations and similar to MaSp1 simulations (Fig.1 of the main paper). The Tyr residues (green) and Pro (purple) are at or close to the surface (see Table 3), burying the hydrophobic poly(Ala) units (pink) primarily in the core region. The radius of gyration (R_g) converged to similar values and are indicated below each structure.

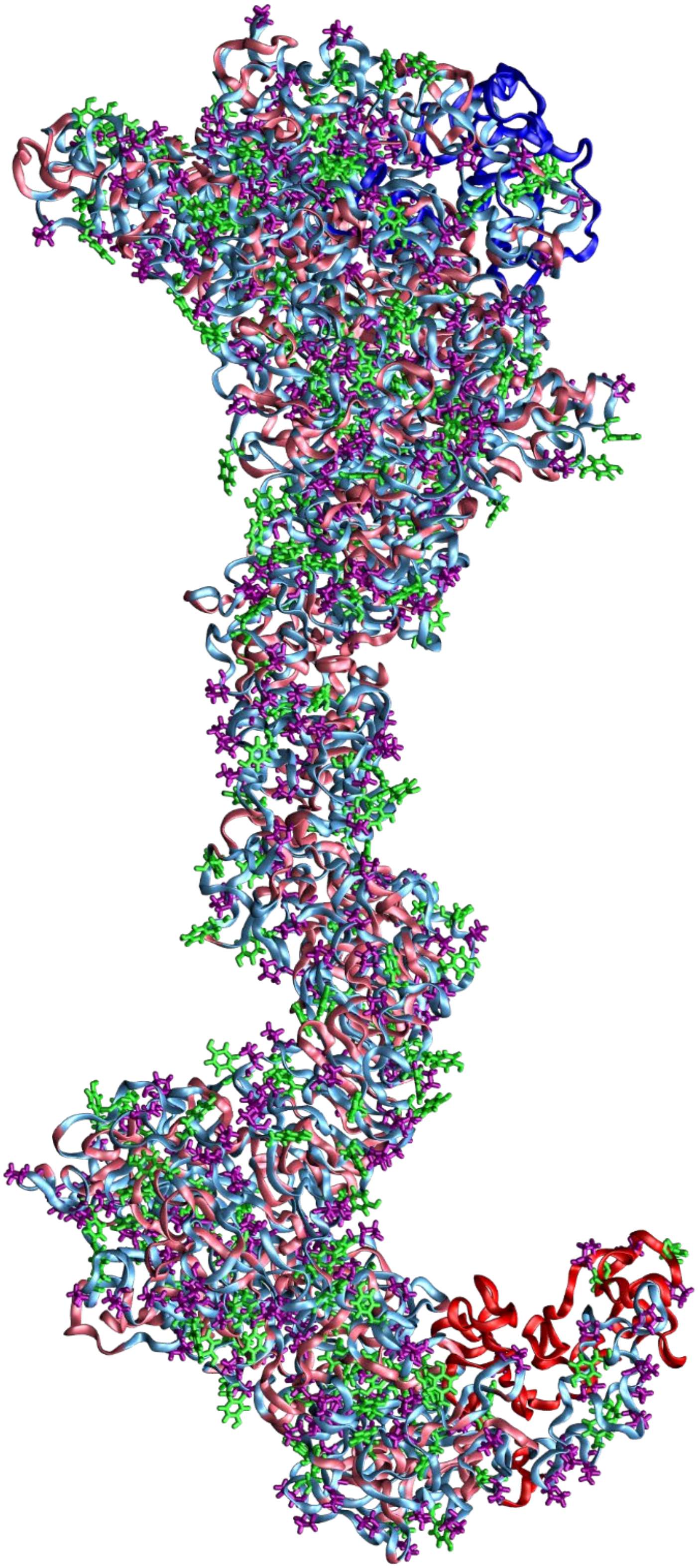

Figure S16. Structure aa_1. Largescale image of the CG-MD models conducted with the MARTINI-2 forcefield in GROMACS for MaSp2 (Run 1). The color coding is as follows, green: Tyr, purple: Pro, pink: Ala, red: N-terminus, blue: C-terminus, light blue: everything else.

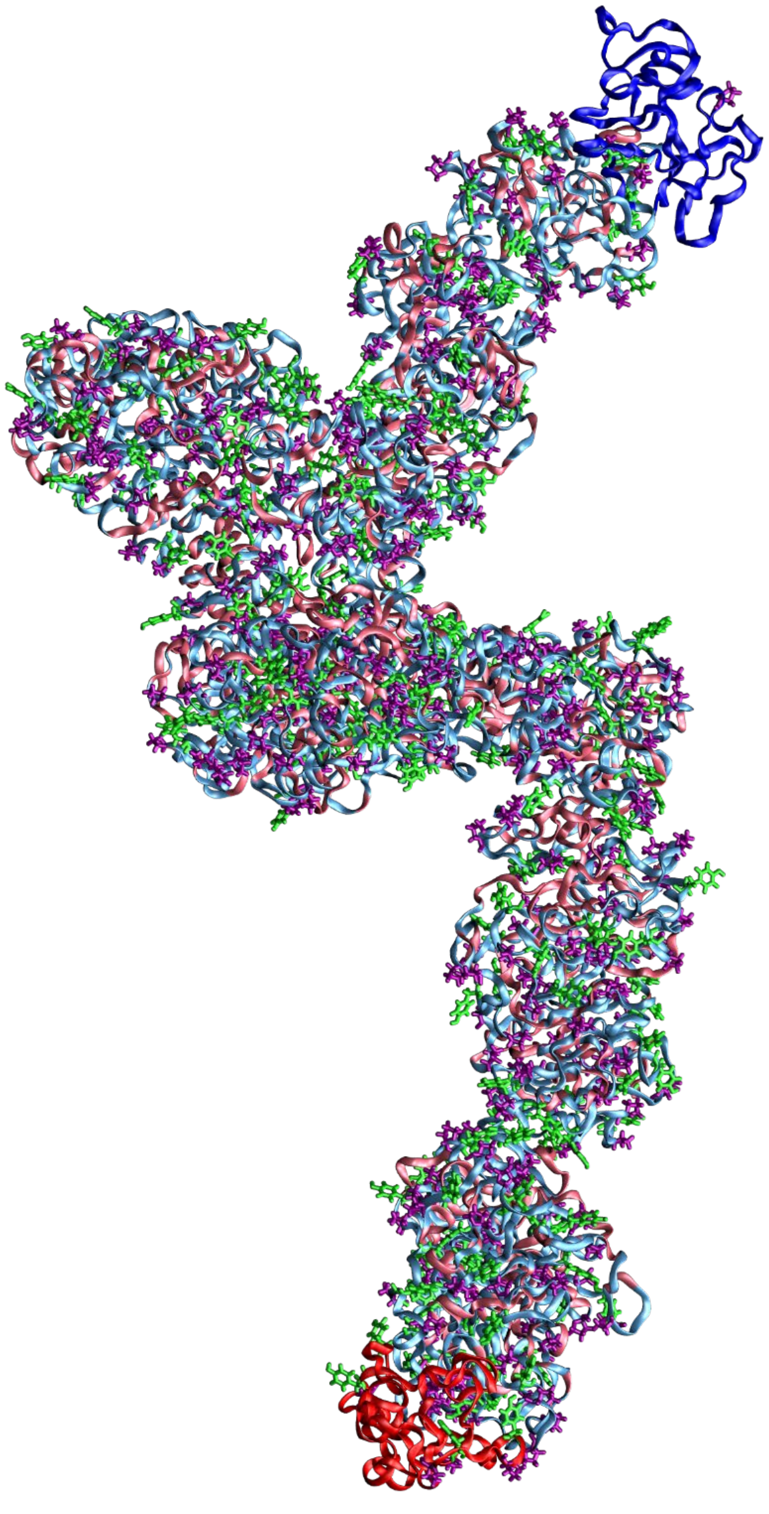

Figure S17. Structure aa_2. Largescale image of the CG-MD model conducted with the MARTINI-2 forcefield in GROMACS for MaSp2 (Run 2). The color coding is as follows, green: Tyr, purple: Pro, pink: Ala, red: N-terminus, blue: C-terminus, light blue: everything else.

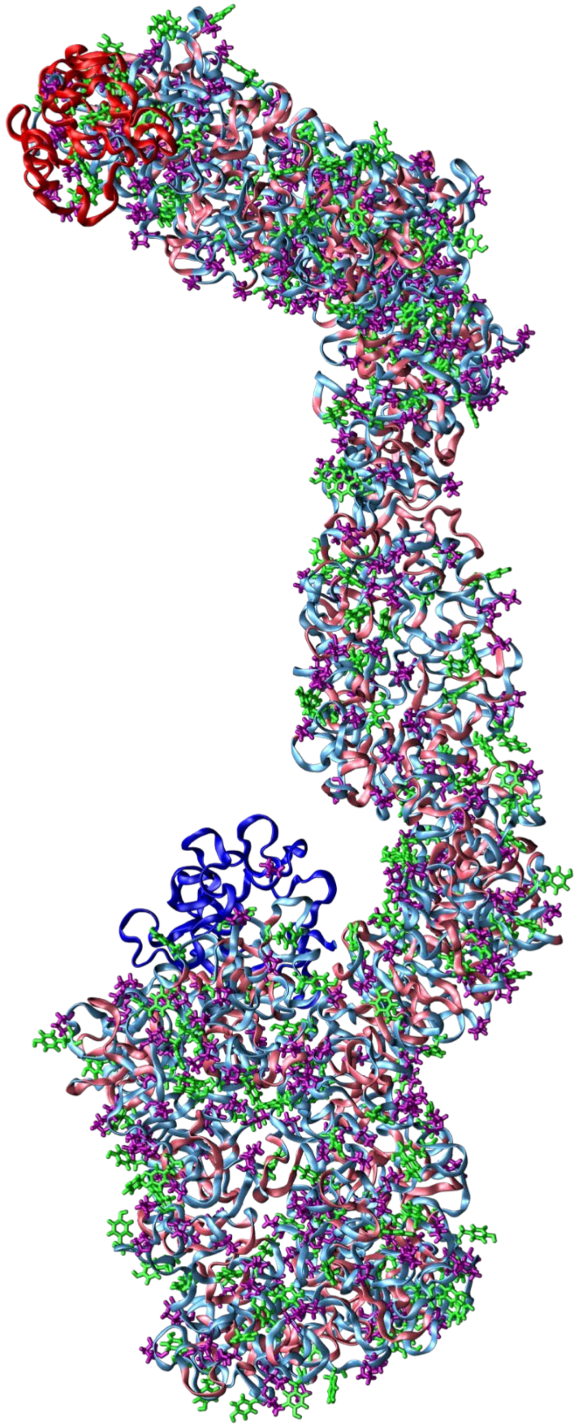

Figure S18. Structure aa_3. Largescale images of the CG-MD models conducted with the MARTINI-2 forcefield in GROMACS for MaSp2 (Run 3). The color coding is as follows, green: Tyr, purple: Pro, pink: Ala, red: N-terminus, blue: C-terminus, light blue: everything else.

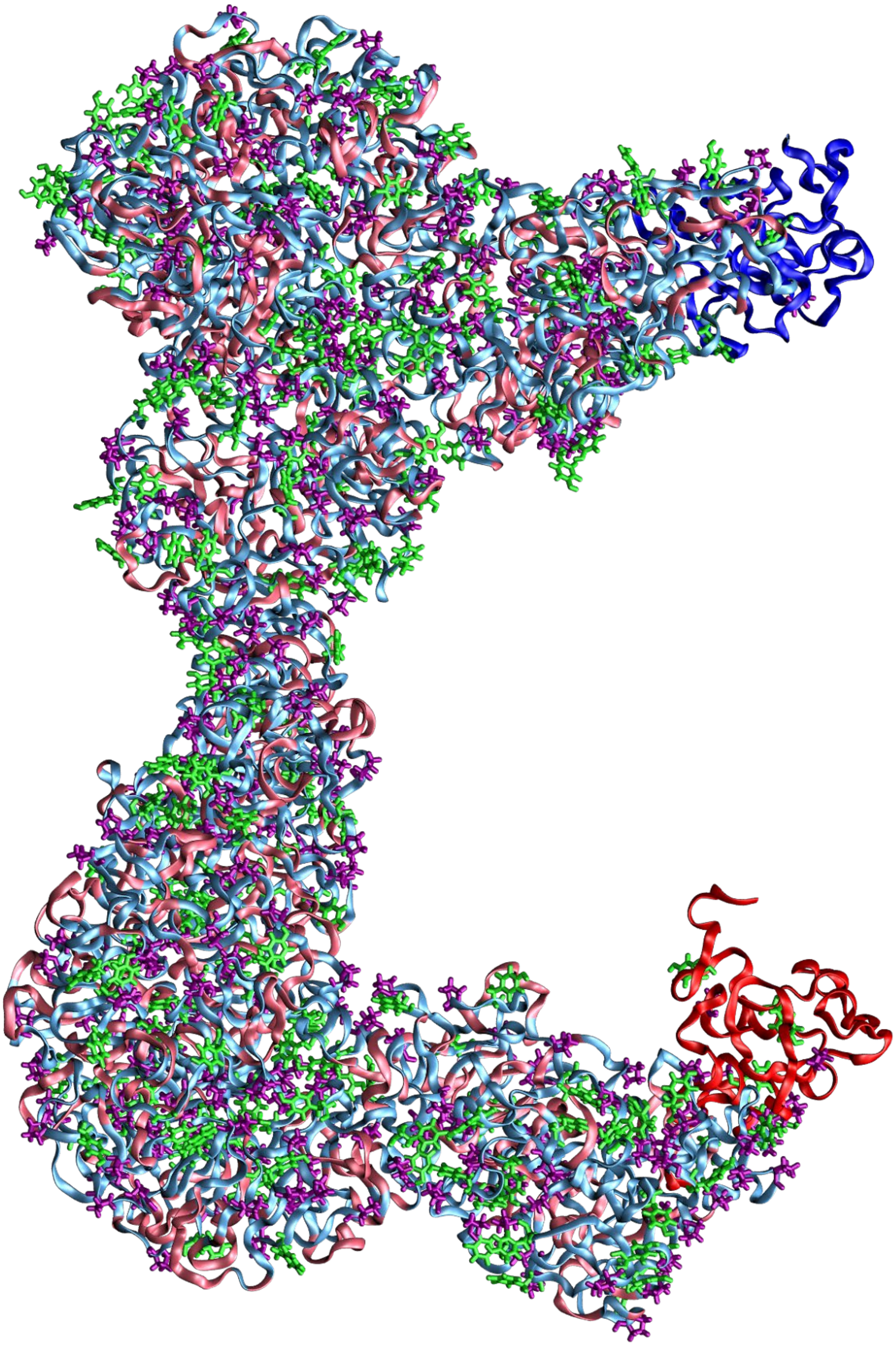

Figure S19. Structure aa_4. Largescale image of the CG-MD model conducted with the MARTINI-2 forcefield in GROMACS for MaSp2 (Run 4). The color coding is as follows, green: Tyr, purple: Pro, pink: Ala, red: N-terminus, blue: C-terminus, light blue: everything else.

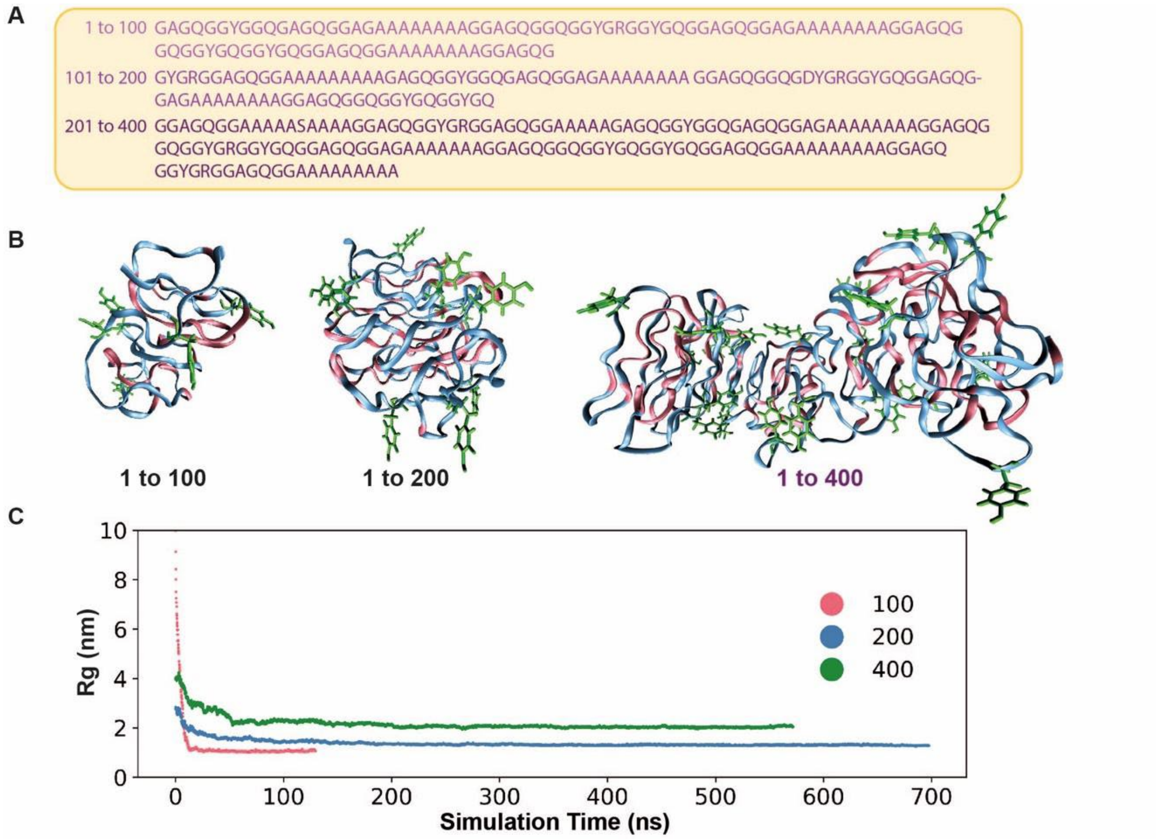

Figure S20. (A) A set of protein fragment lengths (100, 200 and 400 AA) are taken from the MaSp1 repetitive sequence. (B) Simulations were prepared with β-strand starting character using Vesiform to make the backbone and tLeap to generate side chains. The starting structures were then converted to a MARTINI-2 coarse grain mode, solvated, charge balanced, equilibrated, and propagated until the R_g converges, and back converted to atomistic models using CHARMM36 to relax the structures. The same anisotropic tubular structure emerges between 200 and 400 amino acids, with Tyr (green) preferentially located on the surface and poly(Ala) (pink) primarily buried in the core. (C) The smaller fragments were run for a long simulation time to ensure that R_g had indeed converged.

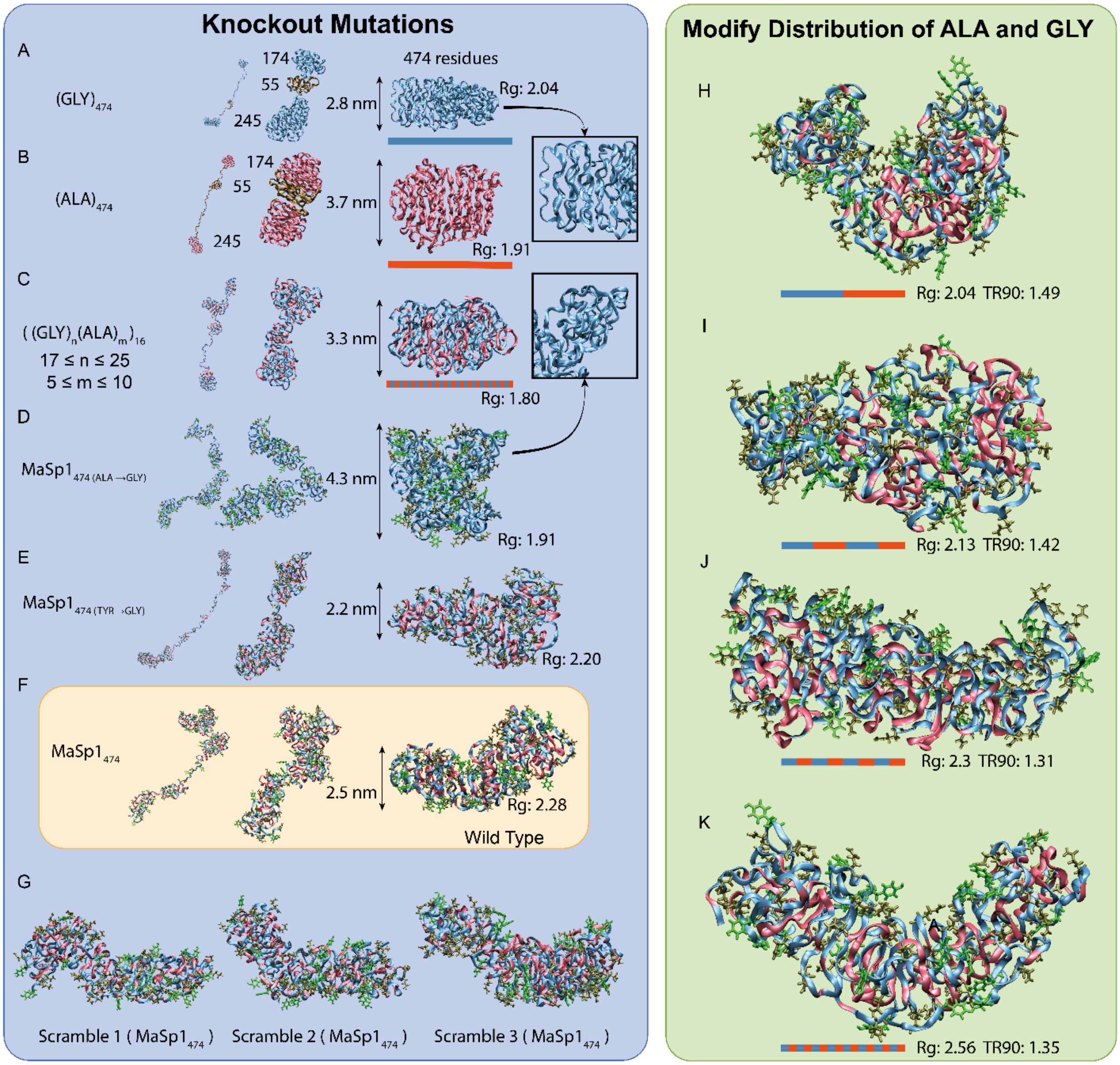

Figure S21. Coarse grain MARTINI2 simulations of a sequence of 474 residues taken from MaSp1 show the effect on the tubular structure of various mutations in the sequence. (A) A chain of 474 Gly, (B) A chain of 474 Ala, (C) All residues in the 474 residue MaSp1 sequence, except Ala, are replaced with GLY, leaving the block co-polymer character of the sequence intact. (D) MaSp1 sequence with all Ala mutated to Gly. (E) All Tyr in the wild-type sequence is mutated to Gly. (F) The 474 residue sub-sequence taken from wild type MaSp1 that is the starting point for all the sequences. (G) Three random scrambles of the wild type sequence. (H) The 16 blocks of poly(Ala) sequences are gathered into a single block and placed at the C-terminus of the sequence. (I) The 16 blocks of poly(Ala) sequence are gathered into two groups and placed at separate parts of the sequence. (J) The poly(Ala) blocks are gathered and placed at four places in the sequence. (K) The poly(Ala) blocks are gathered and placed at 8 places in the sequence.

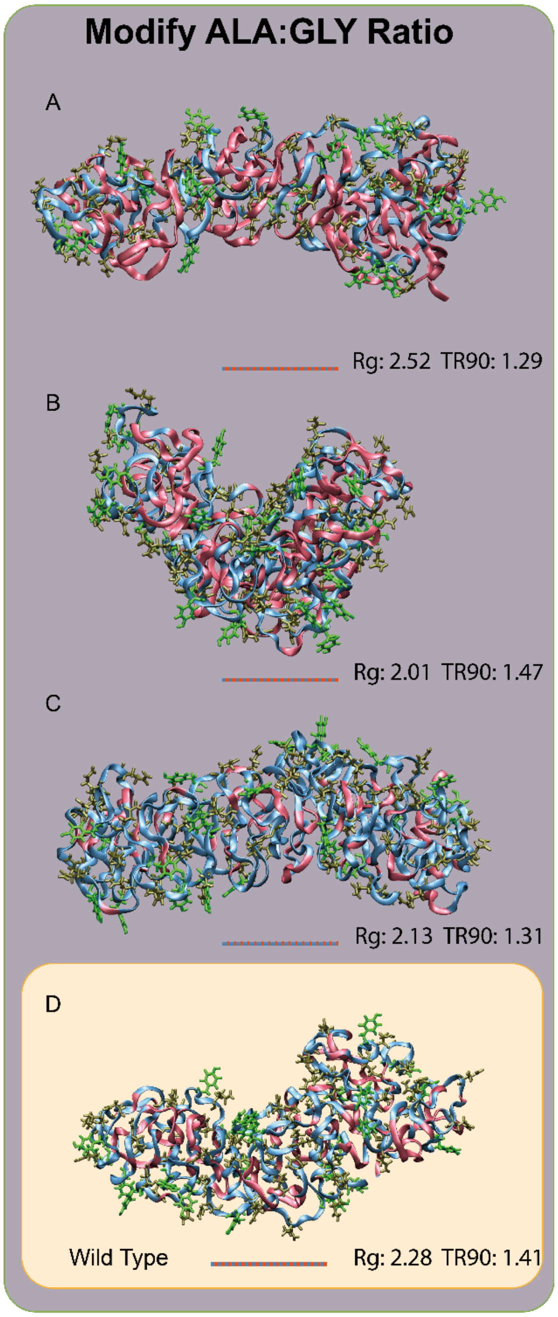

Figure S22. The length of each poly(Ala) region is changed by mutating its surrounding Poly(Gly)/Gly-Gly-X regions to Ala, effectively changing the Ala:Gly ratio. The overall length of the sequence is the same. (A) The Ala regions are expanded by four Ala on each side for every poly(Ala) region, adding 8 Ala to each poly(Ala) run in total. (B) The Ala regions are expanded by 2 Ala on each side of the poly(Ala) region, adding 4 Ala to each poly(Ala) run in total. (C) The poly(Ala) regions are reduced by 2 Ala at the beginning and end of each region, replacing these 4 Ala with Gly.

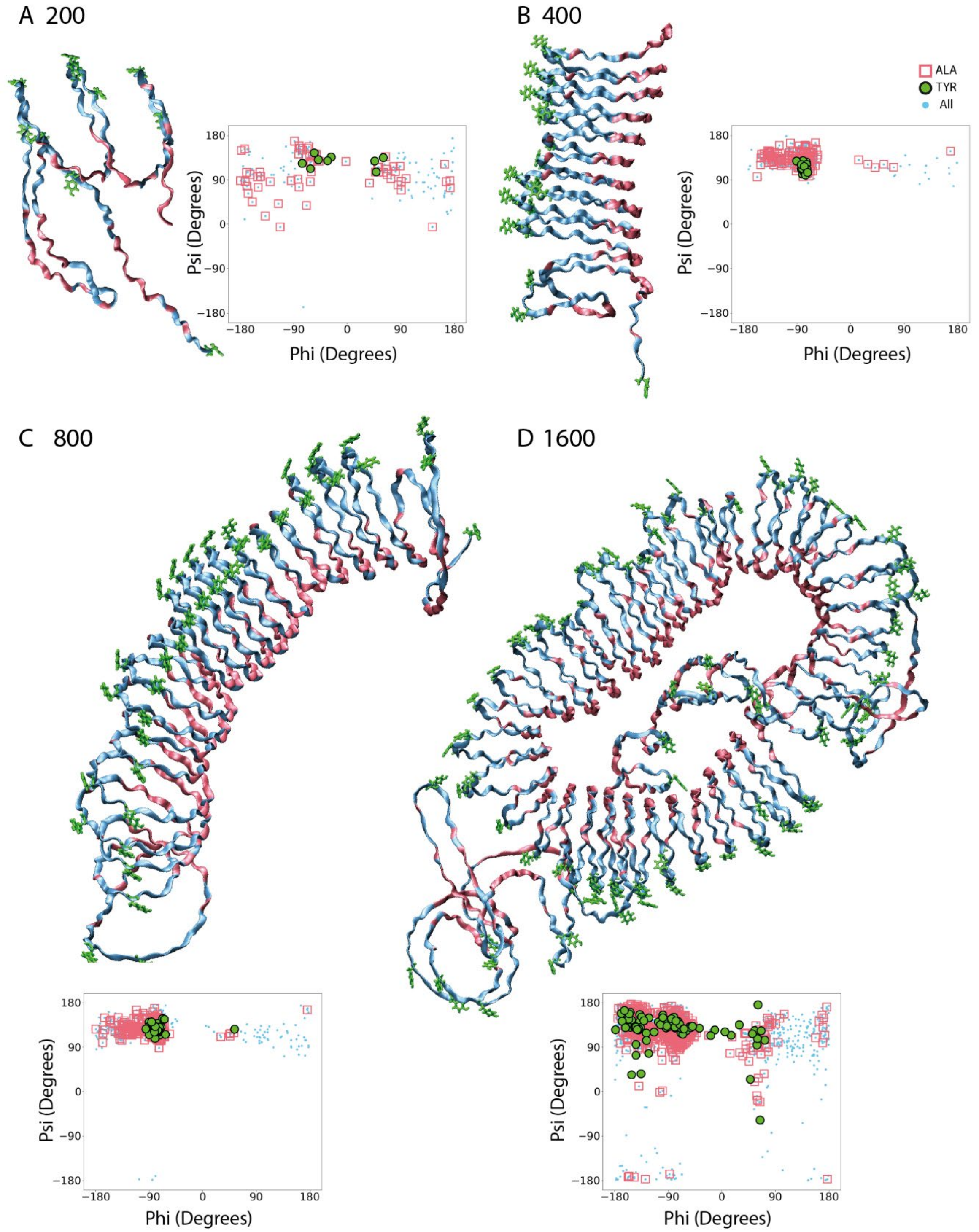

Figure S23. Alphafold2 predictions for varying lengths of the MaSp1 sequence. (A) 200 amino acids, (B) 400 amino acids, (C) 800 Amino acids and (D) 1600 amino acids.

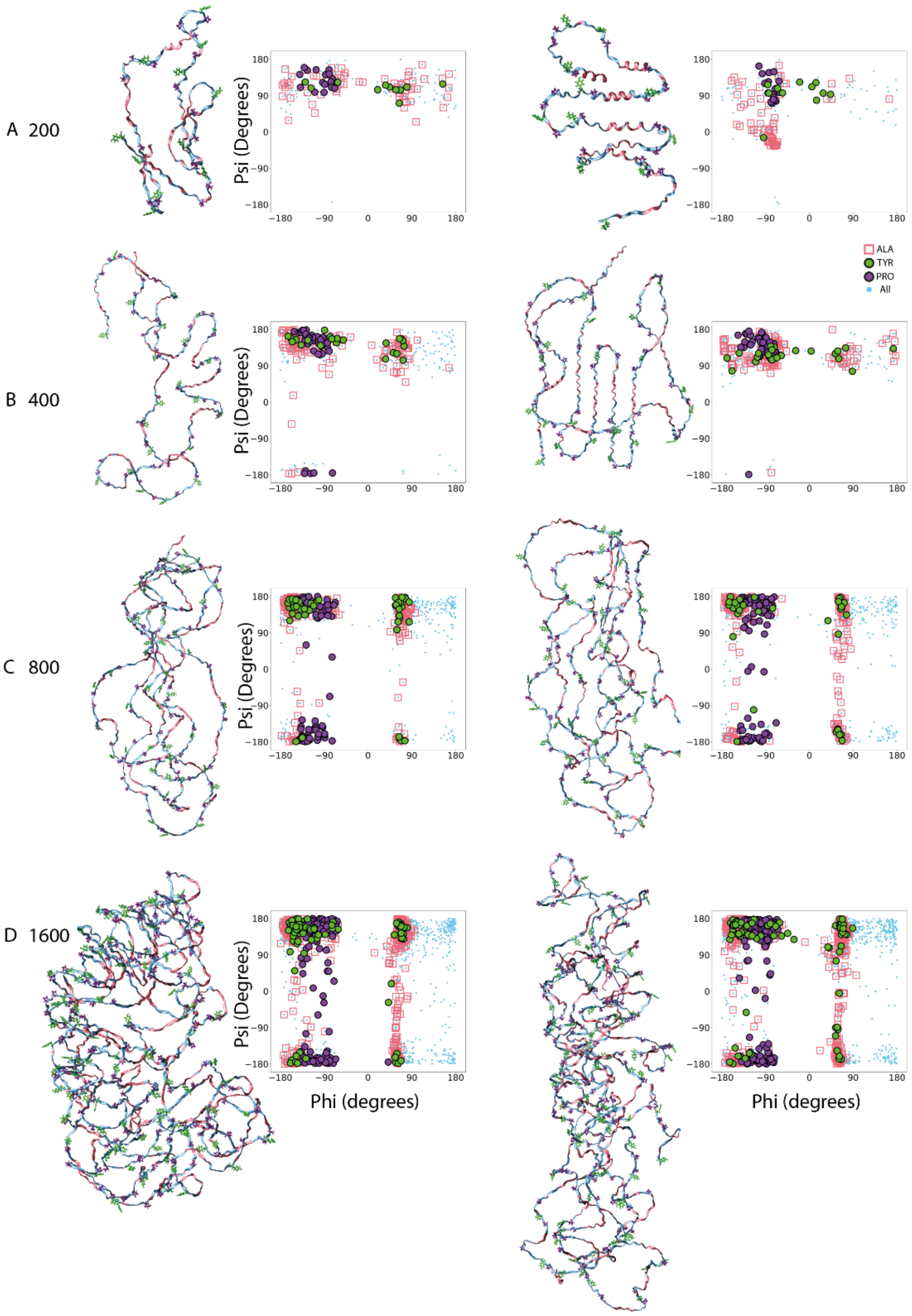

Figure S24. Alphafold2 predictions for varying lengths of the MaSp2 sequence. (A) 200 amino acids, (B) 400 amino acids, (C) 800 Amino acids and (D) 1600 amino acids.

MTWSTRLALSFLFVLCTQSLYALAQANTPWSSKANADAFINSFISAASNTGSFSQDQMEDMSLIGNTLMAAMDNMGGRITPSKLQA
LDMAFASSVAEIAASEGGDLGVTTNAIADALTSAFYQTTGVVNSRFISEIRSLIGMFAQASANDVYASAGSSGGGGYGASSASAAS
ASAAAPSGVAYQAPAQAQISFTLRGQQPVSYGQGGAGPGGAGAAAAAAAAAGGAGQGGQGGYGQGGYGQGGAGQGGSGAAAAAAAA
AGGTGQGGAGQGGAGAAAAAAAAAGGAGQGGQGGYGQGGYGQGGTGQGGAGAAAAAAAAGGAGQGGQGGYGQGGYGQGGYGQGGSG
AAAAAAAAAGGAGQGGQGGYGQGGYGQGGAGQGGAGAAAAAAAAAGGAGQGGYGRGGAGQGGAAAAAAAAAGAGQGGYGGQGAGQG
GSGAAAAAAAAGGAGQGGQGGYGQGGYGQGGSGAAAAAAAAGGAGQGGQGGYGQGGYGQGGAGQGGAGAAAAAAAAGGAGQGGQGG
YGQGGYGQGGAGQGGAGAAAAAAAAGGAGQGGQGGYGQGGYGQGGAGQGGAGAAAAAAAAGGAGQGGQGGYGQGGYGQGGAGQGGA
AAAAAAAAGGAGQGGYGRGGAGQGGAAAAAGAGQGGYGGQGAGQGGAGAAAAAAAAGGAGQGGQGGYGRGGYGQGGA**GQGGAGAA**A
AAAAAGGAGQGGQGGYGQGGYGQGGAGQGGAAAAAAAAGGAGQGGYGRGGAGQGGAAAAAAAAAGAGQGGYGGQGAGQGGAGAAAA
AAAAGGAGQGGQGDYGRGGYGQGGAGQGGAGAAAAAAAAGGAGQGGQGGYGQGGYGQGGAGQGGAAAAASAAAAGGAGQGGYGRGG
AGQGGAAAAAGAGQGGYGGQGAGQGGAGAAAAAAAAGGAGQGGQGGYGRGGYGQGGAGQGGAGAAAAATAAGGAGQGGQGGYGQGG
YGQGGAGQGGAAAAAAAAAGGAGQGGYGRGGAGQGGAAAAAAAAAGAGQGGYGGQGAGQGGAGAAAAAAGGAGQGGQGGYGRGGYG
QGGAGQGGAGAAAAAAAAGGAGQGGQGGYGQGGYGQGGAGQGGAAAAAAAAAGGAGQGGYGRGGAGQGGAAAAAGAGQGGYGGQGA
GQGGAGAAAAASRGAGQGGQGGYGRGGYGQGGAGQGGAGAAAAAAAAGGAGQGGQGGYGQGGYGQGGAGQGGAAAAAAAAGGAGQG
GYGRGGAGQGGAAAAAGAGQGGYGGQGAGQGGAGAAAAAAAAGGAGQGGQGGYGRGGYGQGGA**GQGGAGAA**AAAAAAGGAGQGGQG
GYGQGGYGQGGAGQGGAAAAAAAAAGGAGQGGYGRGGAGQGGAAAAAAAAAGSGQGGYGGQGAGQGGAGAAAAAAAAGGAGQGGQG
GYGRGGYGQGGAGQGGAGAAAAAAAAGGAGQGGQGGYGQGGYGQGGAGQGGAAAAAAAAAAGGAGQGGYGRGGAGQGGAAAAAGAG
QGGYGGQGAGQGGAGAAAAAAAAGGAGQGGQGGYGRGGYGQGGAGQGGAGTAAAAAAAGGAGQGGQGGYGQGGYGQGGAGQGGAAA
AAAAAAGGAGQGGYGRGGAGQGGAAAAAAAAAGAGQGGYGGQGAGQGGAGAAAAAAAAGGAGQGGQGGYGRGGYGQGGA**GQGGAGA**
AAAAAAAGGASQGGQGGYGQGDYGQGGAGQGGAAAAAAAAGGAGQGGYGRGGAGQGGAAAAAGAGQGGYGGQGAGQGGAGAAAAAA
AAGGAGRGGQGGYGRGGYGQGGAGQGGAGAAAAAAAAGGAGQGGQGGYGQGGYGQGGTGQGGAAAAAAAAAGGAGQGGYGRGGAGQ
GGAAAAAAAAAGAGQGGYGGQGAGQGGAGAAAAAAAAGGAGQGGQGGYGRGGYGQGGA**GQGGAGAA**AAAAAAGGAGQGGQGGYGQG
GYGQGGYGQGGAGQGGAAAAAAAAGGAGQGGYGRGGAGQGGAAAAAGAGQGGYGGQGAGQGGAGAAAAAAAAGGAGQGGQGGYGRG
GYGQGGA**GQGGAGAA**AAAAAAGGAGQGGQGGYGQGGYGQGGAGQGGAAAAAAAAAGGAGQGGYGRGGAGQGGAAAAAAAAAGSGQG
GYGGQGAGQGGAGAAAAAAAAGGAGRGGQGGYGRGGYGQGGAGQGGAGAAAAAAAAGGAGQGGQGGYGQGGYGQGGYGQGGAGQGG
AAAAAAAAAAGGAGQGGYGRGGAGQGGAAAAAGAGQGGYGGQGAGQGGAGAAAAAAAAGGAGQGGQGGYGRGGYGQGGA**GQGGAGA**
AAAAAAAGGAGQGGQGGYGQGGNGQGGAGQGGAAAAAAAAGGAGQGGYGRGGAGQGGAAAAAAAAAGAGQGGYGGQGAGQGGAGAA
AAAAAAGGAGQGGQGGYGRGGYGQGGA**GQGGAGAA**AAAAAAGGASQGGQGGYGQGDYGQGGAGQGGAAAAAAAAGGAGQGGYGRGG
AGQGGAAAAAGAGQGGYGGQGAGQGGAGAAAAAAAAGGAGRGGQGGYGRGGYGQGGAGQGGAGAAAAAAAAGGAGQGGQGGYGQGG
YGQGGAGQGGAAAAAAAAAGGAGQGGYGRGGAGQGGAAAAAGAGQGGYGGQGAGQGGAGAAAAAAAAGGAGRGGQGGYGRGGYGQG
GAGQGGAGAAAAAAAAGGAGQGGQGGYGQGGYGQGGAGQGGAAAAAAAAVGGAGQGGYGRGGAGQGGAAAAAAAAAAGSGQGGYGG
QGAGQGGAGAAAAAAAAGGAGQGGQGGYGGGGYGQGGAGQGGAGAAAAAAAAGGAGQGGQGGYGQGGYGQGGAGQGGAAAAAAAAA
GGAGQGGYGRGGAGQGGAAAATGAGQGGYGGQGAGQGGAGAAAAAAAAGGAGQGGQGGYGRGGYGQGGAGQGGAGAAAAAAAAGGA
GQGGQGGYGQGGYGQGGAGQGGAAAAAAAAGGAGQGGYGRGGAGQGGAAAAAAAAAGAGQGGYGGQGAGQGGAGAAAAAAAAGGAG
QGGQGGYGRGGYGQGGA**GQGGAGAA**AAGGAGQGGQGGYGQGGYGQGGAGQGGAAAAAAAAAAGGAGQGGYGGYGQQGGAGAAAAAA
SGPGQIYYGPQSVAAPAAAAASALAAPATSARISSHASALLSNGPTNPASISNVISNAVSQISSSNPGASACDVLVQALLELVTAL
LTIIGSSNIGSVNYDSSGQYAQVVTQSVQNAFA

Figure S25. Full amino acid sequence of *L. hesperus* MaSp1, used in coarse grain simulation runs (see our database). The GQGGAGAAAAAAAAG sequential sequence fully assigned by solution NMR and used for CS-Rosetta structure calculations is highlighted in yellow. This exact repeating unit occurs 39 times in the sequence and when other related sequences, highlighted in red, with shorter poly(Ala) runs are included, accounts for ~20% of the MaSp1 repetitive region. This repeat, a 100-residue continuous repeat highlighted in green and 474-residue sequence underlined was used in atomistic MD simulations (Figure S28, S32). Large portions highlighted in blue of the 100-residue repeat occur multiple times in the MaSp1 sequence. Note that some overlap occurs between the yellow highlight and blue highlight. Such overlap is bolded.

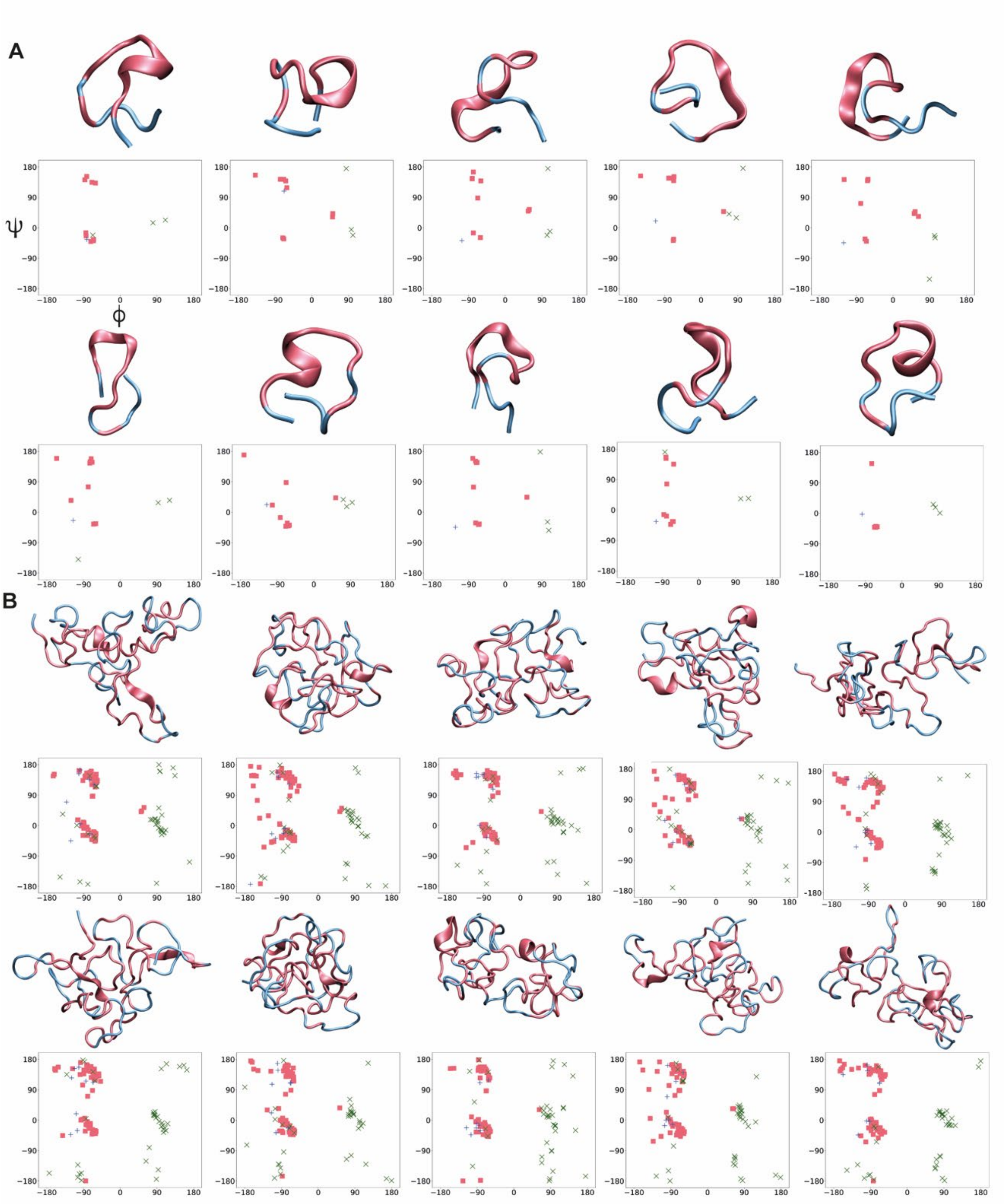

Figure S26. Top ten lowest energy structures determined with CS-Rosetta from solution NMR determined chemical shifts (*SI Appendix* Table S5) for (A) 15-residue and (B) 120-residue GQGGAGAAAAAAAAG repeating unit. The Ramachandran plot for each structure is shown below with Ala indicated by red squares, Gly by green x and Gln by blue +. The Ramachandran plot shows strong clustering for residues exhibiting Type I and Type II β-turns (Fig. S27) with nearly no evidence for β-strand and low population of helices. Pink represents Ala in structures.

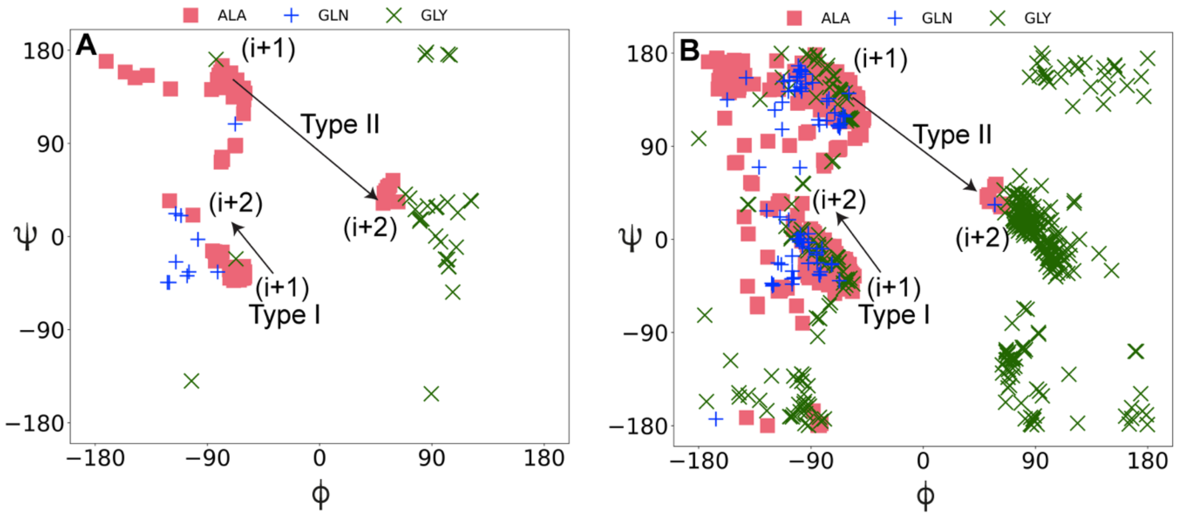

Figure S27. Ramachandran plots for CS-Rosetta determined top ten lowest energy structures. The (A) 15-residue and (B) 120-residue plots are shown. The Ramachandran plots show strong clustering in the Type I and Type II β-turn regions exemplifying that they are the dominant secondary structure in addition to unstructured (random coil) domains.

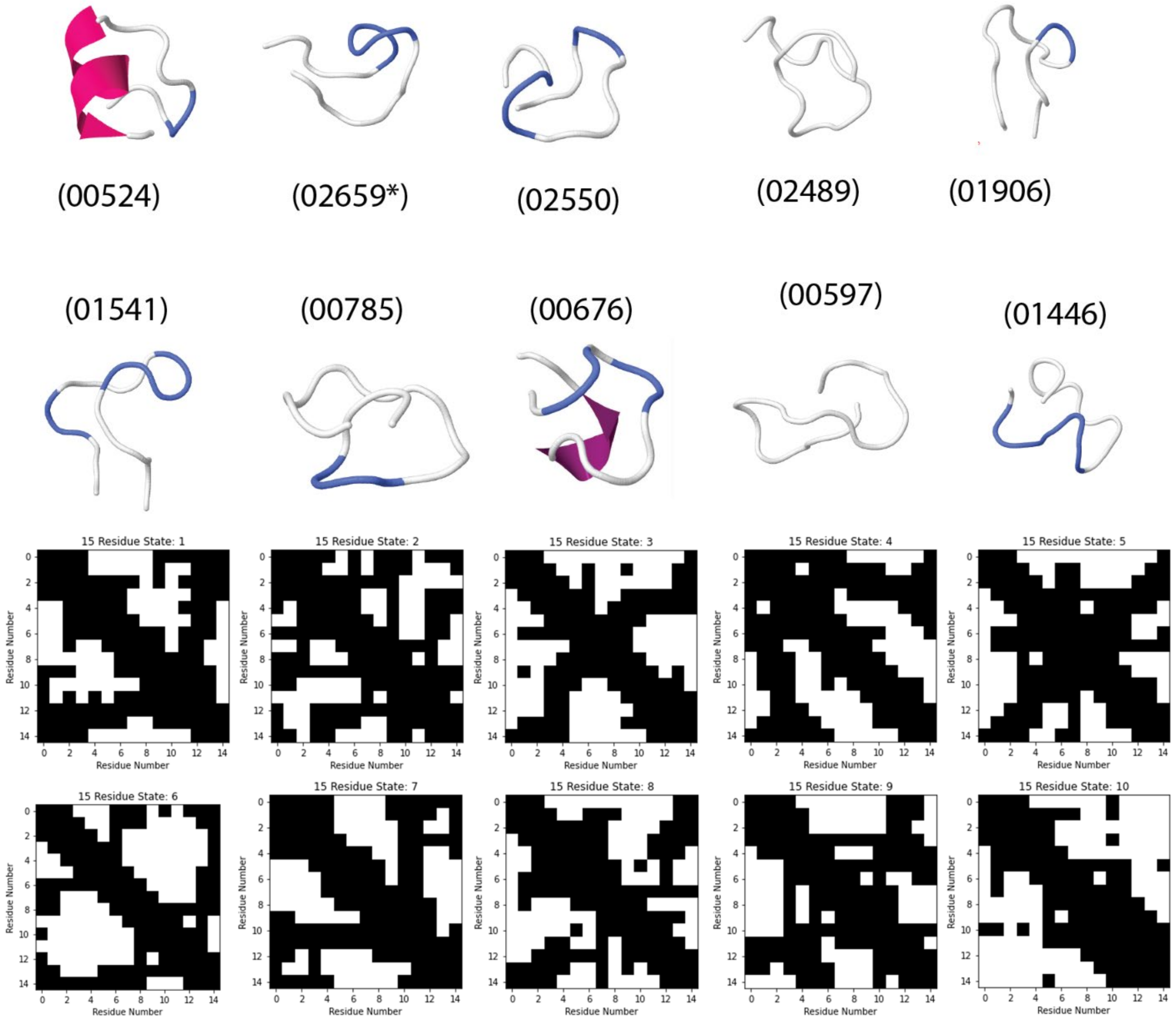

Figure S28. (Top) Top ten lowest energy structures from CS-Rosetta for the 15-residue GQGGAGAAAAAAAAG repeat domain. Standard Jmol color codes apply. Yellow: β−sheet, pink: α-helix, purple: 3_{10}-helix, blue: β-turn, white: random coil. (*) indicates the lowest energy structure. (Bottom) Residue-residue contact maps for structures are shown.

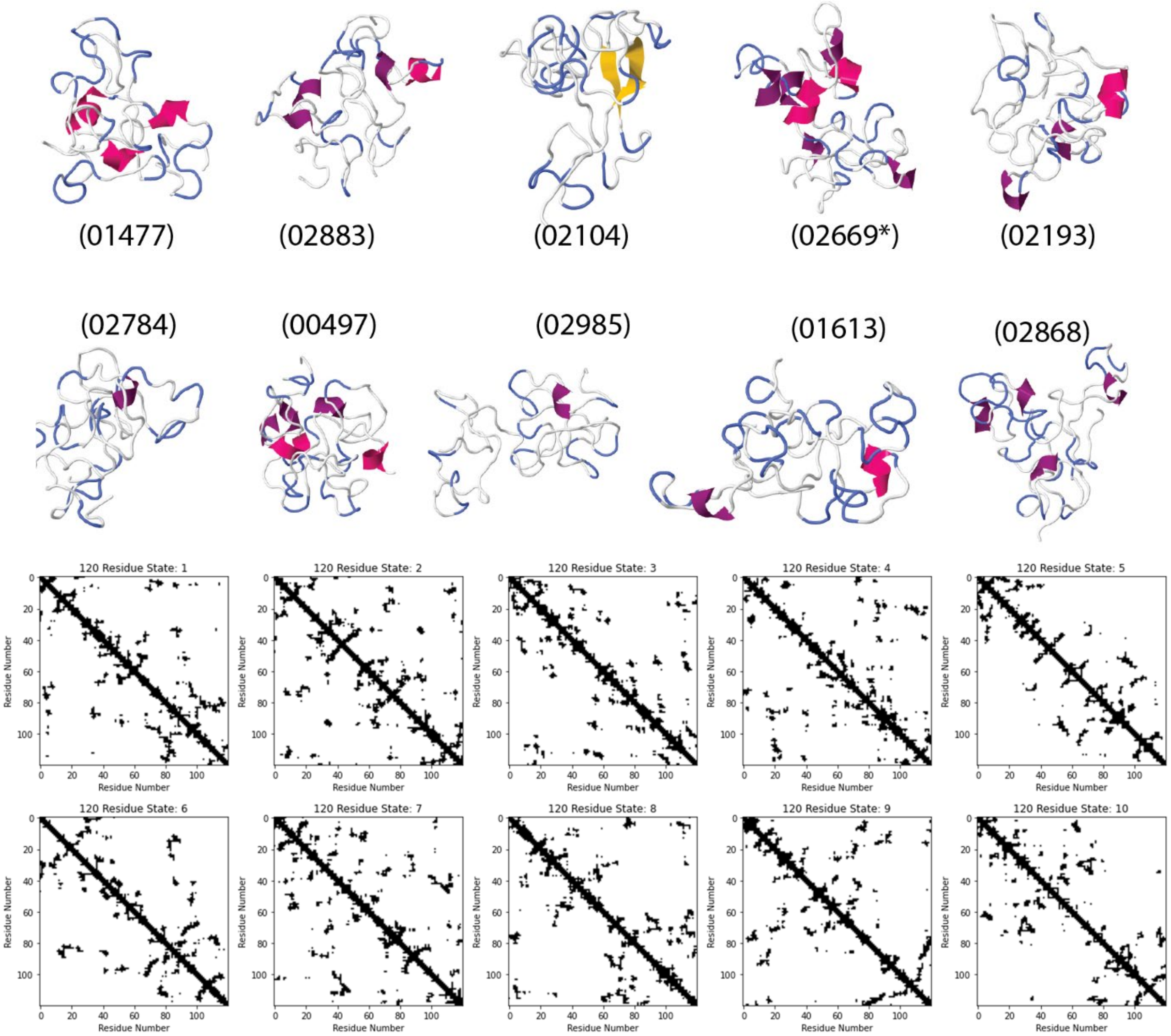

Figure S29. (Top) Top ten lowest energy structures from CS-Rosetta for the 120-residue GQGGAGAAAAAAAAG repeat domain. Standard Jmol color codes apply. Yellow: β−sheet, pink: α-helix, purple: 3_{10}-helix, blue: β-turn, white: random coil. (*) indicates the lowest energy structure. (Bottom) Residue-residue contact maps for structures.

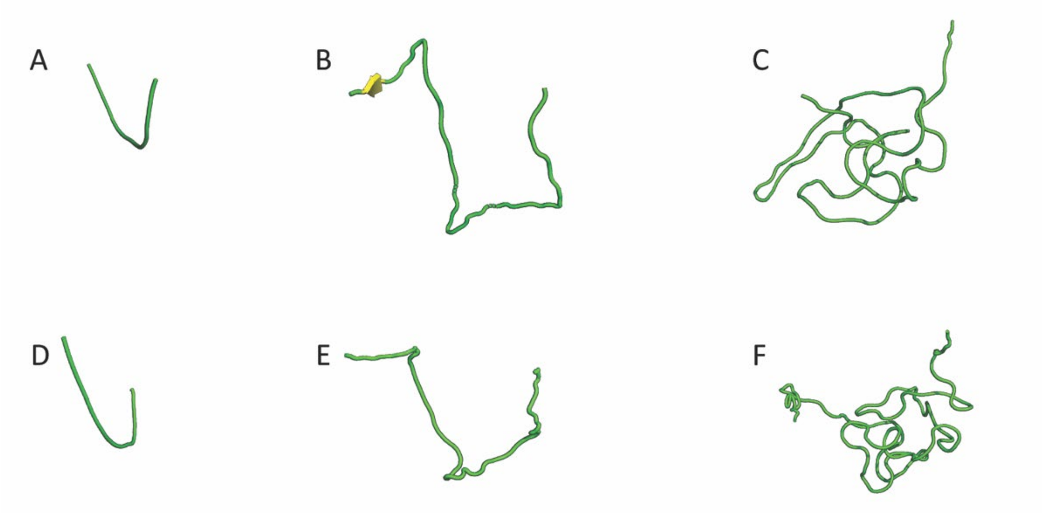

Figure S30. Average structures determined from atomistic MD simulations for (A, D) 15-, (B, E) 60- and (C, F) 100-residue (see Fig. S25) sequences using the CHARMM36m and CHARMMIDPSFF force fields, respectively.

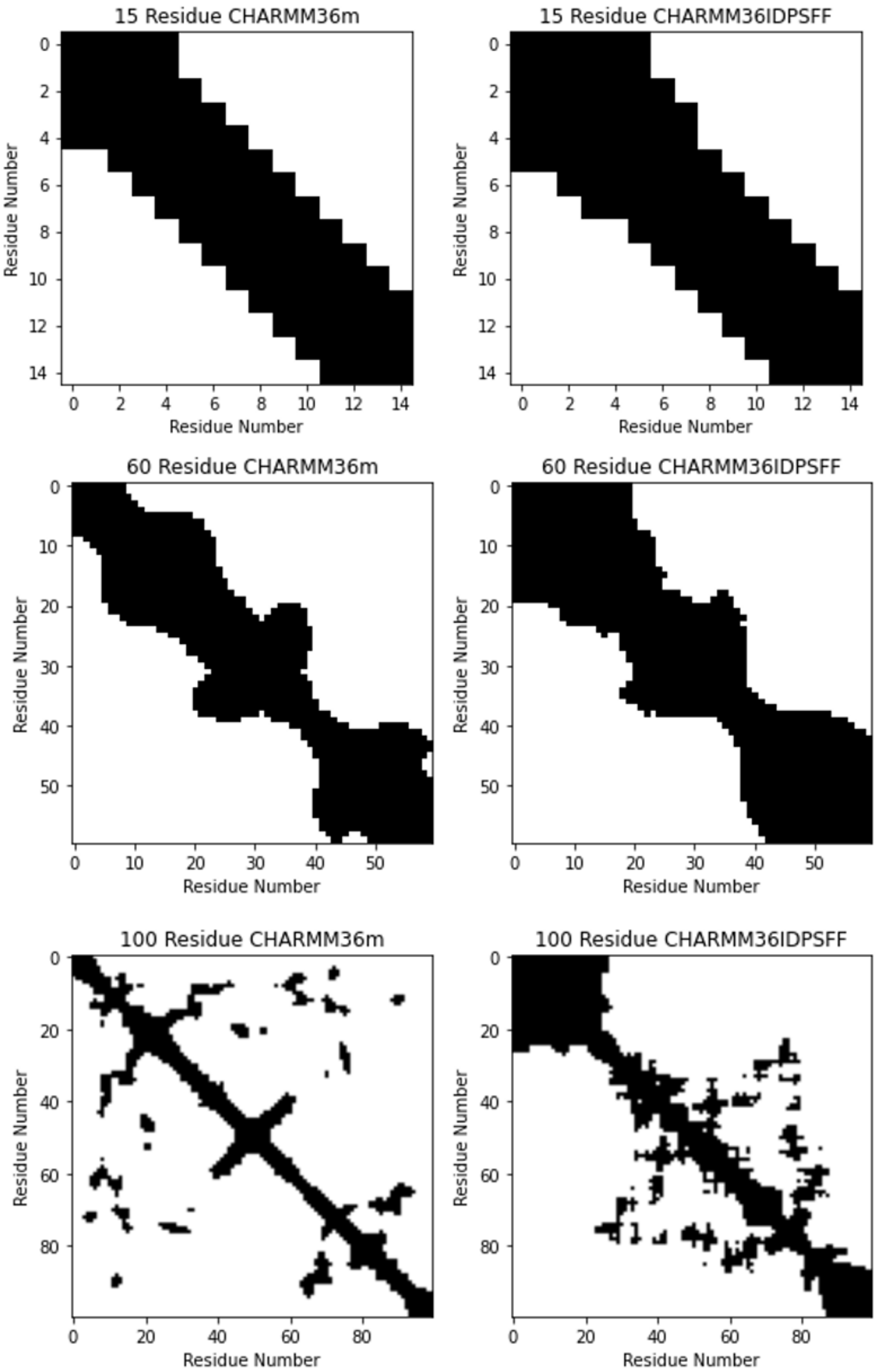

Figure S31. Residue-residue contact maps for average structures determined from atomistic MD simulations for 15-, 60- and 100-residue sequences using the CHARMM36m and CHARMMIDPSFF force fields (see Figure S30).

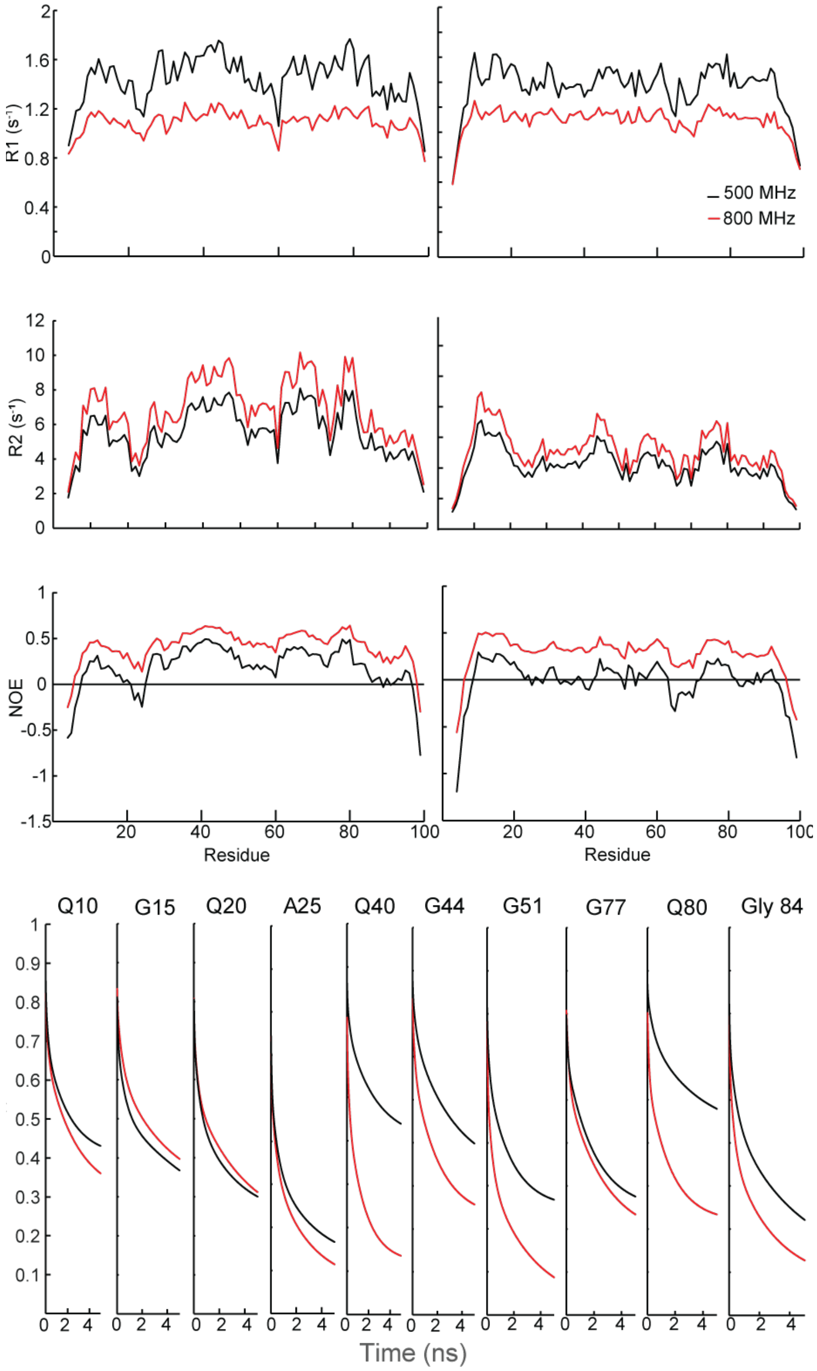

Figure S32. NMR relaxation parameters (R1, R2 and NOE) at 500 (black) and 800 MHz (red) for the 100-residue MaSp1 simulations calculated from the C(t) (bottom) that were determined from CHARMM36m (red) and CHARMM36IDPSFF (black) MD trajectories with SpinRelax (19). The MaSp1silk protein chain is highly dynamic as evidenced by the rapidly decaying C(t) for all residues. The high degree of dynamics is reflected in the NMR relaxation parameters. Specifically, the average NOE is -0.05/0.25 and 0.14/0.38 at 500/800 MHz for CHARMM36m and CHARMM36IDPSFF, respectively.

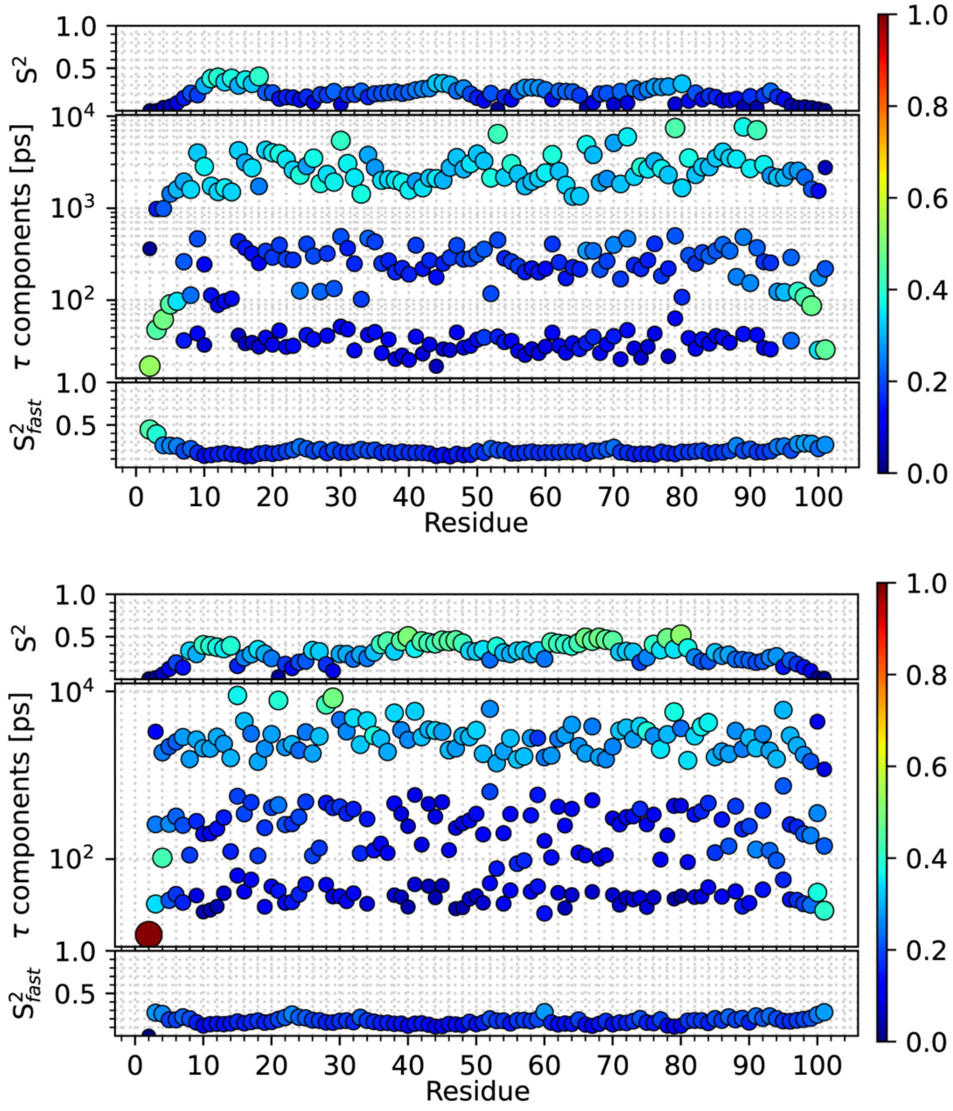

Figure S33. Order parameters and motional timescales determined from CHARMM36m (top) and CHARMM36IDPSFF (bottom) MD trajectories for 100-residue spidroin sequence with SpinRelax. The three subplots show the order parameter, S^2, the set of motional parameters each containing a timescale, τ, and a magnitude indicated in color (right), and the fast motions order parameter, S^2_{fast}. The CHARMM36IDPSFF structure is slightly more rigid presumably due to a higher occurrence of tight β-turns and some helical structures (see Table S6). The average S^2 is 0.29 and 0.37 for the two forcefields, respectively.

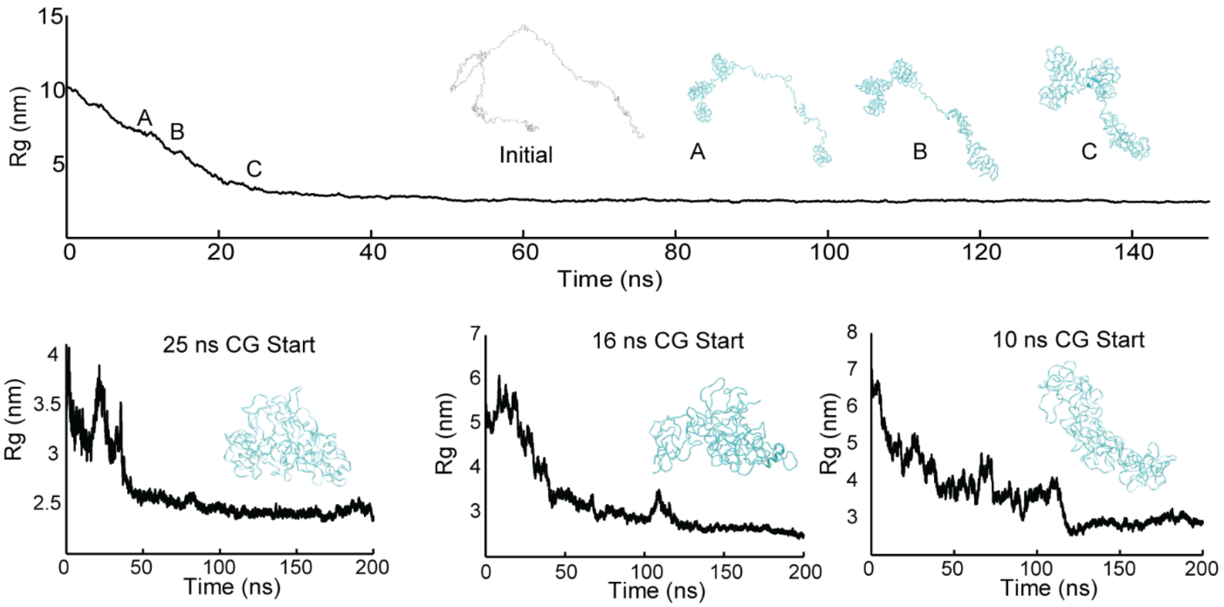

Figure S34. R_g plot as a function of simulation time for 474-residue sequence simulated with MARTINI-2 (Top). The initial structure and simulated structure following different CG-M2 simulation times, (A) 10 ns (B) 16 ns and (C) 25 ns, are shown inset. Different MARTINI starting structures were back-mapped to atomistic and used in CHARMM36m simulations (bottom). The final atomistic structures are shown following 200 ns total simulation time inset. Atomistic structure exhibits a tubular shape reminiscent of the full length M2 models when a 10 ns starting structure is used with similar geometric parameters (Table 2).

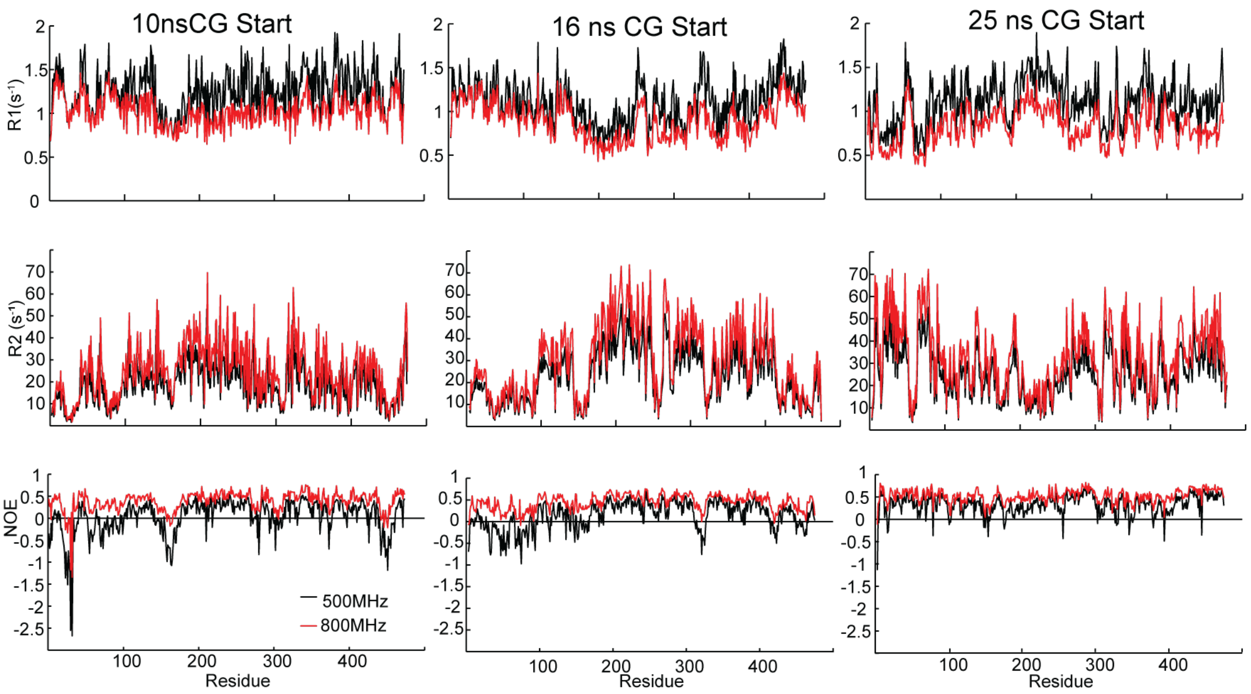

Figure S35. NMR relaxation parameters (R1, R2 and NOE) at 500 (black) and 800 MHz (red) for the 474-residue MaSp1 simulations calculated from the C(t) (Fig. S34) that were determined from CHARMM36m MD trajectories with SpinRelax. The initial staring structure in the atomistic simulation was from 10, 16 and 25 ns M2 timestamps (left, middle, right). The MaSp1silk protein chain displays variable dynamics that depend on the compactness of the starting structure. The higher degree of dynamics for the tubular atomistic structure is reflected in the NMR relaxation parameters and rapidly decaying C(t). Specifically, the average NOE is 0.03/0.39 at 500/800 MHz for CHARMM36 trajectory of the tubular morphology (10 ns CG-M2 timestamp start) is close to experimental values (see Table S7). R2 is higher than anticipated presumably due to motional averaging on the μs-ms timescale not captured in the MD simulation (see discussion main text regarding R1 and R2 results).

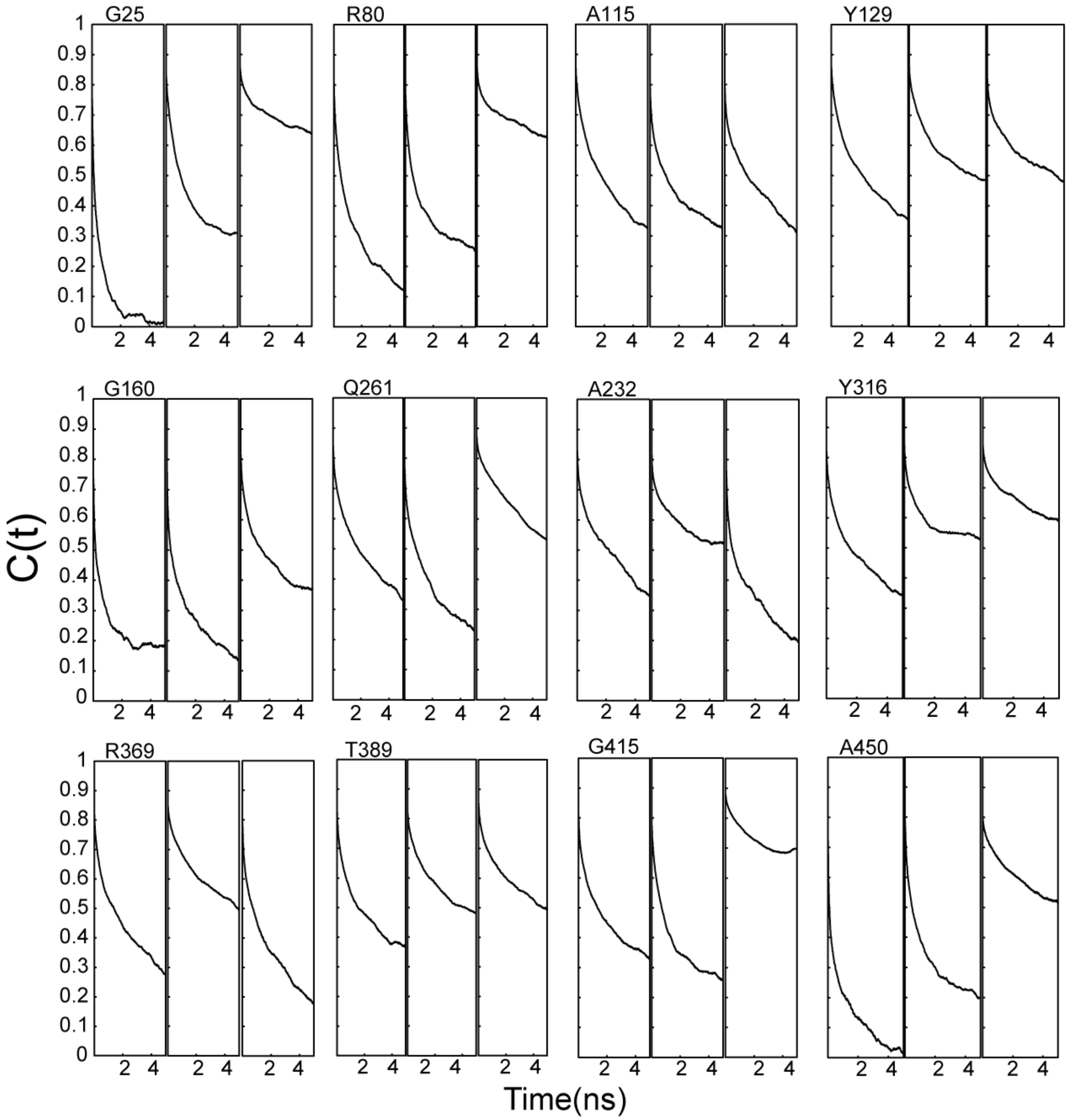

Figure S36. Representative C(t) curves from the 474-atomistic CHARMM36m MD simulations starting from different CG-M2 structures. Twelve different residues across the protein chain are shown above each plot. Panels are 10, 16 and 25 ns CG-M2 starting structure timestamps (left to right). Generally, C(t) curves display less rapid decay for more compact starting structures with the simulation starting from the 10 ns CG-M2 timestamp being the most dynamic.

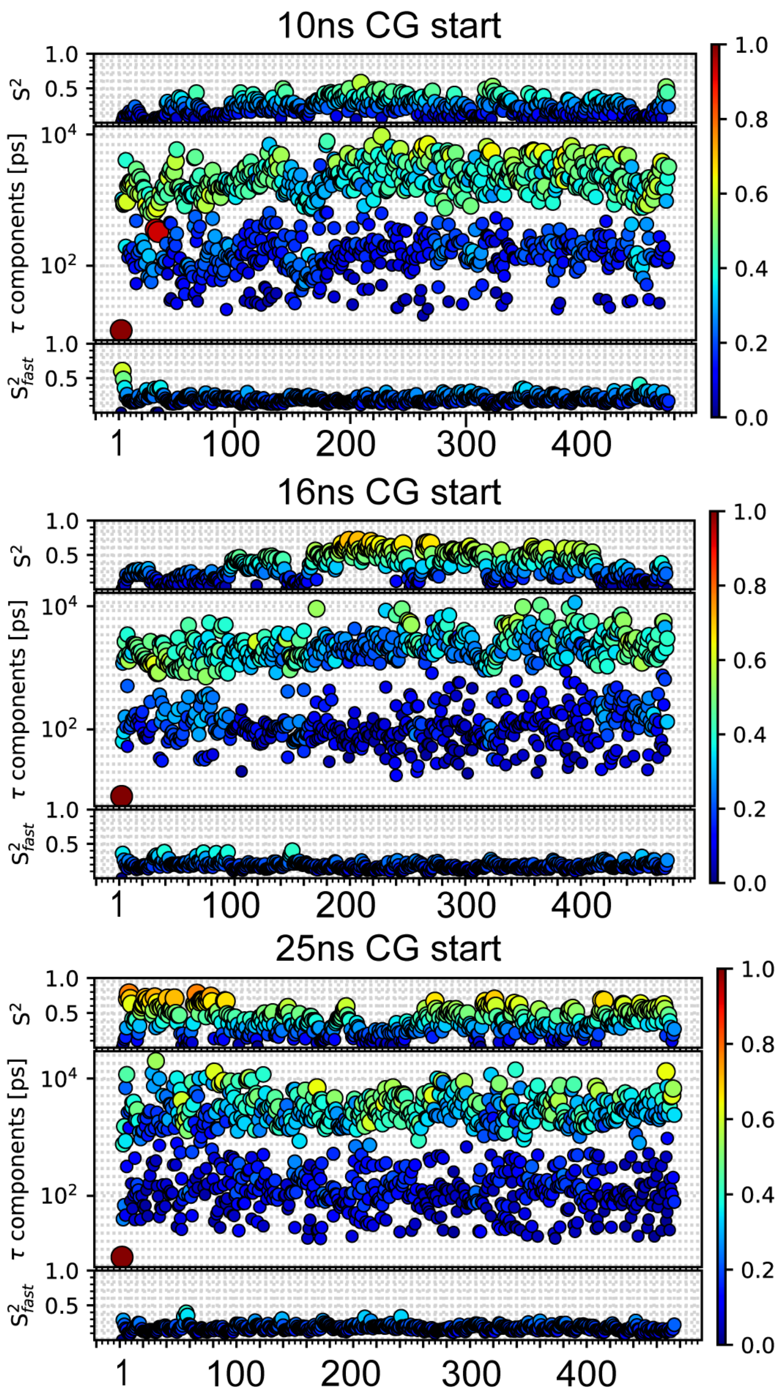

Figure S37. Order parameters and motional timescales determined from CHARMM36m MD trajectories for 474-residue spidroin sequence with SpinRelax. The initial starting structure in the atomistic simulation was from 10, 16 and 25 ns M2 timestamps. The MaSp1silk protein chain displays variable dynamics that depend on the compactness of the starting structure. The higher degree of dynamics for the tubular atomistic structure is reflected in the rapidly decaying C(t) curves compared to other two more compact starting structures (Figure S34). The three subplots show the order parameter, S^2, the set of motional parameters each containing a timescale, τ, and a magnitude indicated in color (right), and the fast motions order parameter, $S^2{}_{fast}$. The 10 ns start shows S^2 ~0.32 on average similar to the 100-resdiue atomistic models while, the 16 and 25 ns starts exhibit higher S^2 ~0.39 and ~0.46 on average, respectively.

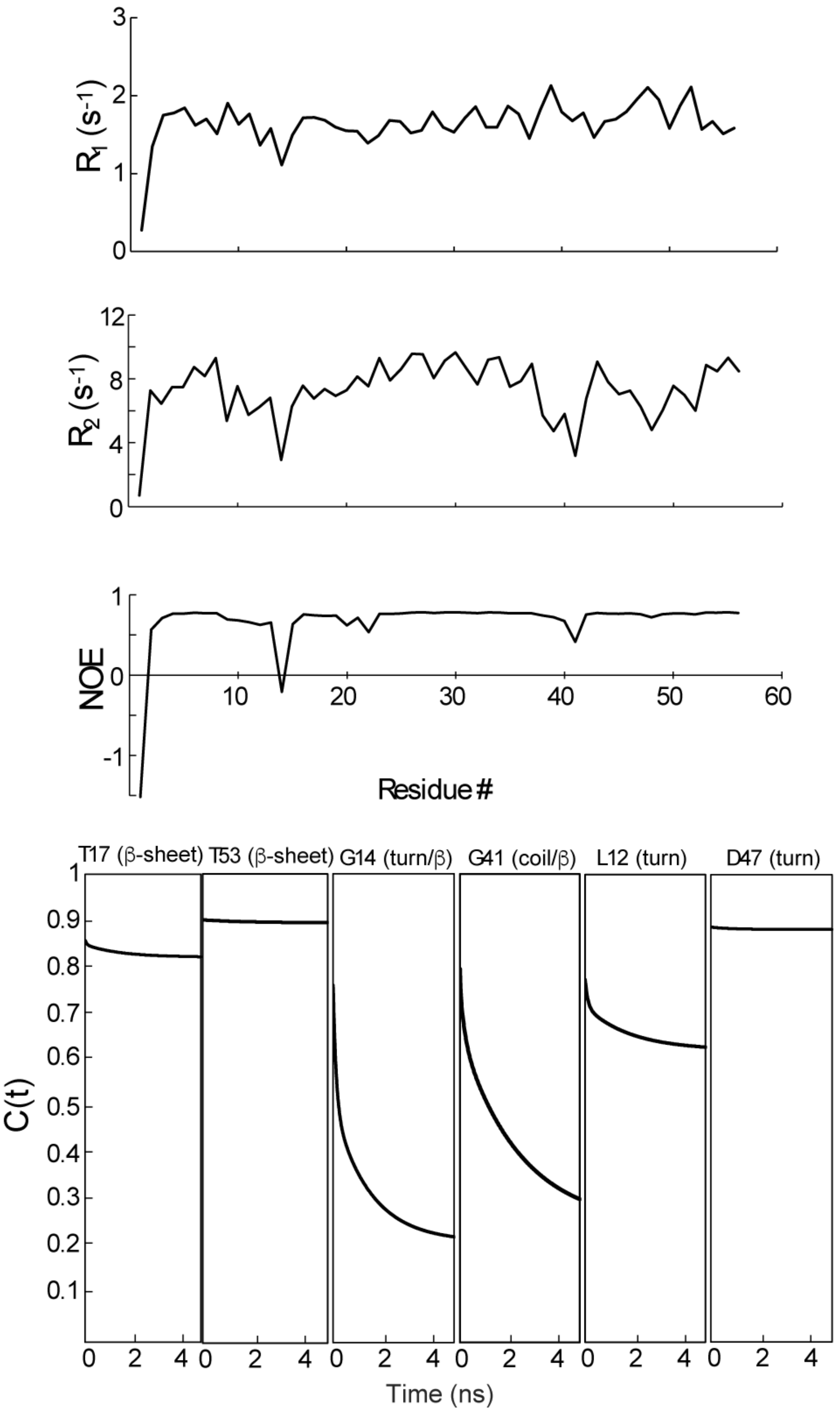

Figure S38. NMR relaxation parameters (R1, R2 and NOE) at 600 MHz for the model protein, GB3, calculated from the correlation functions, C(t), that were determined from CHARMM36m MD trajectories with SpinRelax (19). GB3 is a folded globular protein that is mostly rigid as evidenced by NOE values ~0.7-0.8 with the exception of a few regions including G14 (turn/β), G41 (coil/β) and L12 (turn) that each display decaying C(t) indicating varying degrees of rapid local backbone dynamics. These results agree with those shown in the SpinRelax manuscript (19).

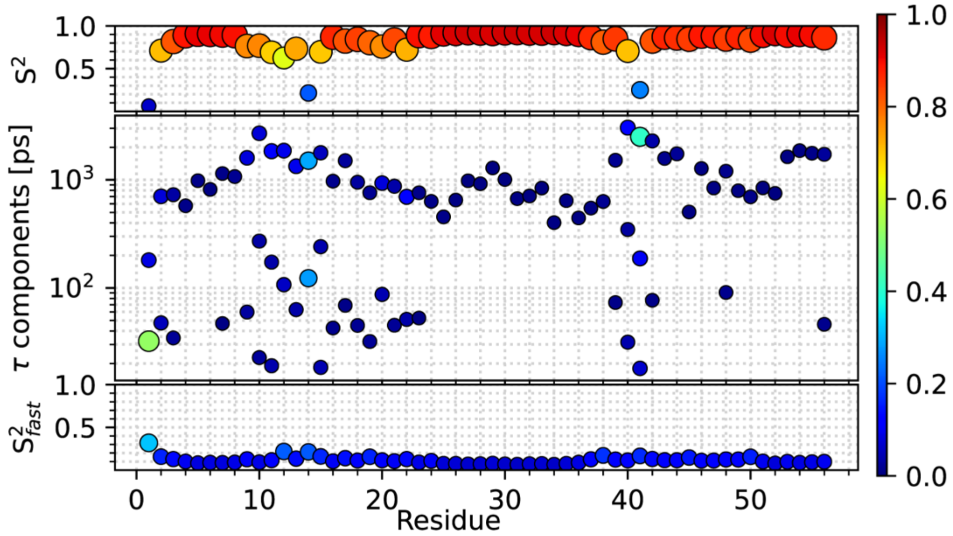

Figure S39. Order parameters and motional timescales determined from CHARMM36m MD trajectories for GB3 with SpinRelax. The three subplots show the order parameter, S^2, the set of motional parameters each containing a timescale, τ, and a magnitude indicated in color (right), and the fast motions order parameter, S^2_{fast}. Rigid (>0.8), intermediate (0.5-0.7) and flexible regions (<0.5) are easily determined from the order parameter (S^2).

Table S1. A-H: 8 distinct pools of structures were curated from various combinations of monomers and dimers drawn from M2 and M3 simulations. The tables correspond to the fitted graphs in Figure S1. The number of structures in each pool is indicated in the parenthesis for each sub-table. The tables report the sub-set of structures from each pool that GAJOE found best explained the experimental data. The numbers in the names refer to the time-stamp in nanoseconds of the structures within their trajectory. The final R_g for each pool comes from Guinier analysis of the fitted curve, rather than a weighted average of the individual R_g. **Observations: C: For M2 Monomers and Dimers** the largest single weighting (86%) is for the starting structure, which is a completely unfolded structure, however tubular structures were given a total of 16% of the weighting. **G: M3 Monomers and Dimers**: Mostly monomers selected across the trajectory and only one dimer. **H: All structures:** Three tubular structures from the early trajectory (130-160 ns) were given a 12% weighting, with a proportion of unfolded to folded around 88%:12%, like the results in table C. It seems that 80-90% disordered and 10-20% compact structures explains the experimental data well.

A: M2 Monomers, χ^2: 74.05, (29)

Name	R_g	DMax	Weight (Fraction)
M2_0000	18.4	55.4	0.88 (21/24)
M2_1000_1	5.8	20.0	0.08 (2/24)
M2_1000_7	5.7	17.8	0.04 (1/24)
**Final Ensemble**	**16.7**	**50.9**	

B: M2-M2 Dimers, χ^2: 12.8, (841)

Name	R_g	DMax	Weight (Fraction)
M2-M2_0000_0000	28.9	99.0	0.53 (8/15)
M2-M2_0000_0180	15.5	57.0	0.07 (1/15
M2-M2_0010_0060	14.7	47.7	0.08 (1/15)
M2-M2_0130_0160	8.5	32.2	0.20 (3/15)
M2-M2_0160_0130	8.3	32.1	0.13 (2/15)
**Final Ensemble**	**8.9**	72.5	

C: M2 Monomers and M2-M2 Dimers, χ^2: 5.8, (870)

Name	R_g	DMax	Weight (Fraction)
M2-M2_0160_0130	8.3	32.1	0.14 (1/7)
M2_0000	18.3	55.4	0.86 (6/7)
**Final Ensemble**	**14.4**	**52.1**	

D: M2-M3 Hybrid Dimers, χ^2: 6.5, (841)

Name	R_g	DMax	Weight (Fraction)
M2-M3_0000_1000	29.1	94.7	0.11 (1/9)
0020_0600	14.8	50.3	0.11 (1/9)
0140_0500	12.2	42.9	0.11 (1/9)
0150_0500	11.7	43.4	0.33 (3/9)
0160_0200	17.4	58.7	0.33 (3/9)
**Finale Ensemble**	**15.1**	**54.9**	

E: M3 Monomers, χ^2: 10.43, (29)

Name	R_g	DMax	Weight (Fraction)
M3_0200	16.6	56.0	0.54 (7/13)
M3_0300	17.3	57.2	0.15 (2/1)
M3_0400	12.9	38.8	0.08 (1/13)
M3_0600	10.9	34.7	0.08 (1/13)
M3_1000	14.9	42.3	0.15 (2/13)
**Final Ensemble**	**15.4**	**51.1**	

F: M3-M3 Dimers, χ^2: 38.4, (841)

Name	R_g	DMax	Weight (Fraction)
0120_0110	34.5	98.4	0.22 (4/18)
0150_0800	29.7	90.7	0.11 (2/18)
0150_1000	31.9	104.5	0.11 (2/18)
0300_1000	31.9	95.0	0.28 (5/18
0600_0080	24.7	72.7	0.28 (5/18)
**Final Ensemble**	**24.8**	**90.9**	

G: M3 Monomers and M3-M3 Dimers, χ^2: 10.0, (870)

Name	R_g	DMax	Weight (Fraction)
M3M3_0180_0180	29.7	82.5	0.09 (1/11)
M3_0200	16.6	56.0	0.27 (3/11)
M3_0300	17.3	57.2	0.18 (2/11)
M3_0400	12.9	38.8	0.09 (1/11)
M3_0600	10.9	34.7	0.18 (2/11)
M3_1000	14.9	42.4	0.18 (2/11)
**Final Ensemble**	**18.0**	**50.7**	

H: M2, M3, M2-M2, M2-M3, M3-M3, χ^2: 2.7, (2581)

Name	R_g	DMax	Weight (Fraction)
M2M2_0160_0130	8.3	32.1	0.055 (1/18)
M2_0140	7.3	24.0	0.055 (1/18)
M3M3_0600_0500	12.3	42.1	0.055 (1/18)
M3_0030	17.1	52.6	0.055 (1/18)
M3_0200	16.6	56.0	0.722 (13/18)
M3_0400	12.9	38.8	0.055 (1/18)
**Final Ensemble**	**13.8**	**50.1**	

Table S2. Geometric parameters of the MaSp models produced in this study. Averaged Contour Length (ACL) is the contour length of the tubular space curve found by averaging the center of mass over successive 150 residue blocks. Max Euclid (MaxE) is the longest Euclidean distance between any two Cα atoms in the structure. R_g is the radius of gyration. R_h is translational hydrodynamic radius computed using HullRad V10. R_t is the tube radius that contains 90% of the residues averaged over two 170 residue sets. R_g/R_h is the shape factor (sphere: 0.77, coil: 0.9 and rod 1.73). R_g/R_t and R_g/MaxE are anisotropy ratios between R_g and the tube radius and MaxE.

Name	ACL (nm)	MaxE (nm)	Rg (nm)	Rh (nm)	Rt (nm)	Rg/Rh	Rg/Rt	Rg/ MaxE
**M2 Monomers 1000 ns – MaSp1**								
**aa_2**	53.0	15.8	5.4	5.9	1.7	0.91	3.13	0.34
**aa_5**	50.8	20.7	6.1	5.9	1.8	1.03	3.41	0.30
**aa_10**	52.4	29.3	8.5	7.1	1.9	1.19	4.59	0.29
**aa_20**	52.3	20.3	6.0	6.3	1.8	0.97	3.39	0.30
**aa_30**	52.9	23.6	7.3	7.1	1.6	1.03	4.52	0.31
**b_1**	51.8	19.2	5.9	6.3	1.7	0.95	3.5	0.31
**b_2**	51.1	21.4	6.6	6.5	1.7	1.02	3.86	0.31
**b_3**	50.7	18.5	5.5	5.7	1.8	0.97	3.11	0.30
**M2 Monomers 1000 ns – MaSp2**								
**aa_1**	67.1	23.8	7.6	6.7	1.9	1.14	4.05	0.32
**aa_2**	62.6	25.7	7.1	7.0	1.9	1.01	3.75	0.28
**aa_3**	59.9	27.2	8.5	7.0	1.8	1.21	4.6	0.31
**aa_4**	64.2	20.4	6.9	6.7	2.0	1.02	3.45	0.34
**AlphaFold – MaSp1**								
**200**	1.1	11.1	3.3	3.5	-	0.95	-	0.30
**400**	6.0	12.2	3.2	3.6	2.1	0.90	1.51	0.26
**800**	14.0	17.8	4.9	5.1	2.2	0.96	2.27	0.28
**1600_1**	57.0	23.7	7.1	7.4	2.6	0.96	2.75	0.30
**1600_2**	52.2	22.6	6.6	7.2	2.4	0.92	2.70	0.29
**1600_3**	46.8	19.2	5.6	6.5	2.2	0.87	2.61	0.29
**1600_4**	36.7	19.4	5.8	6.3	2.3	0.92	2.46	0.30
**1600_0**	89.2	26.6	7.2	6.9	3.2	1.03	2.25	0.27
**Mutational Study**								
**4A Poly(G)**	4.9	6.8	2.0	2.5	1.4	0.83	1.5	0.30
**4B Poly(A)**	4.3	6.1	1.9	2.4	1.7	0.81	1.13	0.31
**4C Poly (AG)**	4.4	6.0	1.8	2.3	1.6	0.77	1.16	0.30
**4D (A->G)**	5.8	5.8	1.9	2.5	1.6	0.75	1.22	0.33
**4E (-TYR)**	5.6	7.7	2.2	2.6	1.4	0.86	1.62	0.29
**4F (WT)**	5.6	7.8	2.3	2.6	1.4	0.89	1.62	0.29
**4G1 Rand**	5.5	7.1	2.1	2.5	1.5	0.87	1.42	0.30
**4G2 Rand**	6.4	8.4	2.5	2.7	1.3	0.93	1.89	0.30
**4G3 Rand**	6.9	9.0	2.7	2.8	1.3	0.97	2.13	0.30
**4H**	5.8	6.4	2.0	2.5	1.5	0.81	1.37	0.32
**4I**	8.1	9.3	2.7	2.6	1.4	0.83	1.50	0.29
**4J**	5.5	7.6	2.3	2.6	1.3	0.88	1.76	0.30
**4K**	6.1	8.3	2.6	2.7	1.4	0.94	1.90	0.31
**1600_0**	89.2	26.6	7.2	6.9	3.2	1.03	2.25	0.27
**Selected M2 Dimers**								
**0ns 0ns**	383.1	107.5	29.9	29.4	-	1.02	-	0.28
**20ns 30ns**	154.3	67.8	23.2	19.1	1.31	1.21	17.71	0.34
**70ns 80ns**	111.8	60.0	17.1	14.7	1.34	1.16	1.15	0.28
**110ns 120ns**	103.8	55.8	14.5	11.3	1.39	1.29	1.08	0.26
Mean (100ns)	***103.4***	***44.5***	***12.4***	***11.3***	***1.37***	***1.10***	***1.27***	***0.28***
**170ns 180ns**	99.9	37.1	11.7	10.2	1.40	1.15	1.22	0.32
**200ns 1000ns**	102.6	35.5	10.6	9.4	1.47	1.13	1.30	0.30
**Atomistic 474 Residues**								

**10 ns (200 ns)**	7.2	9.2	2.9	3.2	1.7	0.91	1.7	0.32
**16 ns (200 ns)**	7.3	8.6	2.5	3.1	1.8	0.81	1.4	0.29
**25 ns (200 ns)**	7.0	7.2	2.4	3.0	2.1	0.80	1.1	0.32
**M3 Monomers**								
**20 ns**	167.2	58.0	17.9	16.0	2.54	1.12	7.0	0.31
**50 ns**	162.1	58.0	18.2	15.8	2.47	1.15	7.4	0.31
**100 ns**	155.1	51.1	17.6	15.5	2.43	1.14	7.2	0.34
**150 ns**	142.6	62.1	18.5	15.5	2.36	1.19	7.8	0.30
**200 ns**	140.9	58.4	17.4	14.3	2.82	1.22	6.2	0.30
**500 ns**	130.1	44.2	13.3	12.3	2.44	1.08	5.5	0.30
**700 ns**	138.3	39.8	12.6	12.2	2.42	1.03	5.2	0.32
**1000 ns**	138.6	43.6	15.2	13.1	2.29	1.16	6.6	0.35
**Selected M3-M3 Dimers**								
**20 ns 30 ns**	376.2	101.85	31.09	24.1	2.41	1.29	12.9	0.31
**70 ns 80 ns**	367.6	85.99	28.14	25.3	2.44	1.11	11.5	0.33
**110 ns 120 ns**	335.8	101.29	34.95	24.1	2.44	1.45	14.3	0.35
**170 ns 180 ns**	323.2	69.90	23.29	20.6	2.35	1.13	9.9	0.33
**200 ns 300 ns**	332.7	81.64	26.83	21.1	2.57	1.27	10.4	0.33
**0500 ns 0600 ns**	300.4	44.18	12.78	14.4	2.46	0.89	5.2	0.29
**0700 ns 0800 ns**	314.0	54.21	18.37	17.4	2.55	1.06	7.2	0.34
**0900 ns 1000ns**	308.3	49.39	16.93	16.9	2.23	1.00	7.6	0.34

Table S3. Solvent accessible surface area occupied by those residues expressed in absolute terms and as a percentage from a range of MD models. M2 Monomers for MaSp1 are averaged over 8 structures and for MaSp2 over 4, with the standard deviation in brackets. Also indicated are the relative proportion of the residues that are buried (inside) and on the surface (outside) as determined by the GetArea algorithm (22). The ratio between the occupied surface area and the number of that type of residue (A/N) or the ratio of outside to inside (O/I) are indicators of residue distribution throughout the structure. Red denotes residues with indicators less than the whole protein, and green denotes residues with indicators greater than the whole protein. Residues colored red are considered predominantly buried residues, whereas those colored green are considered as surface residues.

	N	N(%)	Surface Area	Area(%)	A/N	I%	O%	O/I
**M2 Monomer MaSp1 (8)**								
**ALL**	3132	100.0	73276 (4627)	100.0	1.00	66 (2)	15 (1)	0.23
GLY	1330	42.5	15468 (783)	21.1	0.50	76 (1)	8 (1)	0.11
ALA	1019	32.5	14230 (1345)	19.4	0.60	76 (2)	13 (2)	0.17
SER	76	2.4	2355 (232)	3.2	1.33	51 (3)	26 (6)	0.51
GLN	357	11.4	20570 (1466)	28.1	2.46	34 (4)	31 (4)	0.91
TYR	151	4.8	10408 (993)	14.2	2.95	31 (7)	22 (4)	0.71
ARG	51	1.6	3890 (206)	5.3	3.26	24 (7)	30 (4)	1.25
Others	148	4.8	6355	8.7				
**M2 Monomer MaSp2 (4)**								
**ALL**	3779	100.0	88954 (1291)	100.0	1.00	68 (0.4)	14 (0.4)	0.21
GLY	1267	33.5	12044 (454)	13.5	0.40	81 (1)	5 (0)	0.06
ALA	1176	31.1	14750 (401)	16.6	0.53	79 (1)	11 (1)	0.14
SER	267	7.1	4136 (205)	4.6	0.66	76 (2)	7 (1)	0.09
PRO	324	8.6	14131 (319)	15.9	1.85	37 (2)	36 (3)	0.97
GLN	261	6.9	15738 (543)	17.7	2.56	33 (2)	31 (1)	0.94
TYR	197	5.2	11632 (218)	13.1	2.51	42 (1)	17 (2)	0.40
ARG	68	1.8	5738 (234)	6.5	3.59	20 (5)	35 (4)	1.75
Others	217	5.7	10785	12.1				
**M3 Monomer MaSp1 (50 ns)**								
**ALL**	1730	100.0	139801	100.0	1.00	6	78	13.00
GLY	736	42.5	45213	32.3	0.76	4	76	19.00
ALA	563	32.5	42755	30.6	0.94	6	83	13.83
GLN	199	11.5	4053	2.9	0.25	2	89	44.50
TYR	84	4.9	26762	19.1	3.94	1	83	83.00
Others	148	8.6	21018	15.0	1.76			
**M3 Monomer MaSp1 (150 ns)**								
**ALL**	1730	100.0	137531	100.0	1.00	7	76	10.86
GLY	736	42.5	44952	32.7	0.77	5	75	15.00
ALA	563	32.5	42481	30.9	0.95	6	84	14.00
GLN	199	11.5	4243	3.1	0.27	6	83	13.83
TYR	84	4.9	25335	18.4	3.79	1	83	83.00
Others	148	8.6	20520	14.9	1.74			
**M3 Monomer MaSp1 (1000 ns)**								
**ALL**	1730	100.0	138911	100.0	1.00	6	77	12.83
GLY	736	42.5	45330	32.6	0.77	4	74	18.50
ALA	563	32.5	42477	30.6	0.94	6	82	13.67
GLN	199	11.5	4133	3.0	0.26	3	83	27.67
TYR	84	4.9	25896	18.6	3.84	0	89	inf
Others	148	8.6	21076	15.2	1.77			
**10 ns Coarse Grain 200 ns Atomistic**								
**ALL**	474	100.0	22795	100.0	1.00	32	39	1.22
GLY	219	46.2	6998	30.7	0.66	37	32	0.86
ALA	164	34.6	7799	34.2	0.99	32	45	1.41
GLN	57	12.0	5099	22.4	1.86	21	51	2.43
TYR	24	5.1	2108	9.2	1.83	21	25	1.19
Others	10	2.1	791	3.5	1.65			

**16 ns Coarse Grain (200 ns Atomistic)**									
**ALL**	474	100.0	22818	100.0	1.00	34	38	1.12	
GLY	219	46.2	6990	30.6	0.66	38	32	0.84	
ALA	164	34.6	8083	35.4	1.02	34	41	1.21	
GLN	57	12.0	4861	21.3	1.77	23	53	2.30	
TYR	24	5.1	1998	8.8	1.73	25	33	1.32	
Others	10	2.1	885	3.9	1.84				
**25 ns Coarse Grain 200 ns Atomistic**									
**ALL**	474	100.0	23924	100.0	1.00	30	41	1.37	
GLY	219	46.2	7342	30.7	0.66	34	34	1.00	
ALA	164	34.6	8015	33.5	0.97	30	43	1.43	
GLN	57	12.0	5202	21.7	1.81	19	58	3.05	
TYR	24	5.1	2164	9.0	1.79	29	33	1.14	
Others	10	2.1	1201	5.0	2.38				

Table S4: Alphafold plDDT scores for various subsets of the MaSp1 and MaSp2 sequences presented by column in rank order. The N- and C-termini alone score highly because they are well characterized in the PDB. The N-terminus scores lower because a long linker length was included in that analysis. The number in the first column 200 to 1600 indicates the length of each sequence, but none of the sequences score highly which is expected for an IDP.

Rank	0	1	2	3	4
			**MaSp1**		
NTerm	68.0	67.6	67.1	64.5	61.4
CTerm	90.9	90.8	90.7	89.2	88.4
200	38.2	37.8	37.3	35.6	29.8
400	37.9	34.6	34.0	30.6	29.5
800	34.9	32.6	32.3	27.0	25.5
1600	36.9	34.2	29.9	27.0	26.8
			**MaSp2**		
NTerm	82.0	80.6	29.8	29.3	79.0
CTerm	88.8	88.8	87.5	87.5	87.4
200	39.3	39.1	38.7	38.6	32.2
400	45.1	43.6	41.5	39.7	39.4
800	40.8	38.7	38.1	36.4	36.4
1600	37.3	37.2	35.5	34.0	32.8

Table S5. Solution NMR isotropic chemical shifts for *L. heperus* MaSp1 GQGGAGAAAAAAAAG repeating domain. These chemical shifts were used as inputs for TALOS-N ϕ/ψ restraints and CS-Rosetta structural ensemble determination.

No.	Name	Atom	Chemical shift	No.	Name	Atom	Chemical shift	No.	Name	Atom	Chemical shift	No.	Name	Atom	Chemical shift
1	G	CA	45.5	5	A	CA	52.95	8	A	HB	1.41	12	A	NH	8.14
1	G	NH	8.52	5	A	CB	19.36	8	A	C	175.3	12	A	N15	122.9
1	G	N15	109.9	5	A	NH	8.27	9	A	CA	52.81	12	A	HA	4.26
1	G	HA2	3.89	5	A	N15	123.75	9	A	CB	19.27	12	A	HB	1.41
1	G	HA3	3.89	5	A	HA	4.26	9	A	NH	8.14	12	A	C	175.3
1	G	C	174.3	5	A	HB	1.41	9	A	N15	122.9	13	A	CA	52.81
2	Q	CA	56.09	5	A	C	175.3	9	A	HA	4.26	13	A	CB	19.27
2	Q	CB	29.5	6	G	CA	45.5	9	A	HB	1.41	13	A	NH	8.14
2	Q	NH	8.26	6	G	NH	8.52	9	A	C	175.3	13	A	N15	122.9
2	Q	N15	119.68	6	G	N15	109.9	10	A	CA	52.81	13	A	HA	4.26
2	Q	HA	4.36	6	G	HA2	3.89	10	A	CB	19.27	13	A	HB	1.41
2	Q	HB	3.97	6	G	HA3	3.89	10	A	NH	8.14	13	A	C	175.3
2	Q	C	175.88	6	G	C	174.3	10	A	N15	122.9	14	A	CA	52.81
3	G	CA	45.5	7	A	CA	53.06	10	A	HA	4.26	14	A	CB	19.27
3	G	NH	8.51	7	A	CB	19.52	10	A	HB	1.41	14	A	NH	8.25
3	G	N15	109.85	7	A	NH	8.11	10	A	C	175.3	14	A	N15	123.1
3	G	HA2	3.89	7	A	N15	123.9	11	A	CA	52.81	14	A	HA	4.33
3	G	HA3	3.89	7	A	HA	4.26	11	A	CB	19.27	14	A	HB	1.39
3	G	C	174.3	7	A	HB	1.41	11	A	NH	8.14	14	A	C	175.3
4	G	CA	45.5	7	A	C	175.3	11	A	N15	122.9	15	G	CA	45.5
4	G	NH	8.21	8	A	CA	52.81	11	A	HA	4.26	15	G	NH	8.52
4	G	N15	108.6	8	A	CB	19.27	11	A	HB	1.41	15	G	N15	109.9
4	G	HA2	3.89	8	A	NH	8.14	11	A	C	175.3	15	G	HA2	3.89
4	G	HA3	3.89	8	A	N15	122.9	12	A	CA	52.81	15	G	HA3	3.89
4	G	C	174.3	8	A	HA	4.26	12	A	CB	19.27	15	G	C	174.3

Table S6. DSSP secondary structure quantification for the atomistic MD structures simulated for the 15- , 60-, and 100-residue sequence (Fig. S30), as well as three structures simulated for the 474-residue sequence starting from the CG-M2 simulation times of 10, 16 and 25 ns (Fig. S34). Two force fields were used: [a]CHARMM36m and [b]CHARMMIDPSFF. Secondary structure quantification is from DSSP analysis of the simulated structures expressed as a percent with the average reported (see Methods).

Secondary Structure	15-residue[a]	15-residue[b]	60-residue[a]	60-residue[b]	100-residue[a]	100-residue[b]	474-residue[a] (10ns CG)	474-residue[a] (16ns CG)	474-residue[a] (25ns CG)
α-Helix	2.2	2.2	1.0	6.5	0.1	8.6	2.2	2.5	1.0
β-Bridge	0.2	0.2	4.9	4.6	8.1	2.2	5.1	5.5	6.5
β-Strand	0.1	0.1	5.2	1.8	1.1	0.4	1.9	1.6	2.5
3_{10}-Helix	0.5	0.5	0.9	5.1	1.2	15.0	1.0	1.8	1.2
π-Helix	0.0	0.0	0.0	0.1	0.0	0.4	0.0	0.0	0.0
pp helix	14.7	14.7	12.0	11.8	8.5	4.6	5.9	5.1	5.9
β-Turn	4.5	4.5	7.1	17.5	10.5	26.3	8.7	9.8	9.0
Bend	20.5	20.5	28.5	19.4	30.4	18.1	34.3	30.5	33.4
Unstructured	57.4	57.4	40.3	33.3	40.2	24.6	40.9	43.2	40.6

Table S7. ^{15}N NMR relaxation parameters (R_1, R_2, and heteronuclear NOE) for MaSp spider silk proteins obtained from experiment (24) and calculated from atomistic MD simulations using SpinRelax (19). Simulated values are shown for (a) 100-residue trajectories using CHARMM36m and CHARMM36IDPSFF, and for (b) 474-residue trajectories initialized from CG-M2 structures at 10, 16, and 25 ns. Simulations reproduce key trends in NOE, while R_2 values increase with trajectory length and degree of local order.

	Experiment (500/800MHz)	[a]CHARMM36m (500/800MHz)	[a]CHARMM36IDPSFF (500/800 MHz)	[b]CHARMM36m (CG-10ns) (500/800MHz)	[b]CHARMM36m (CG-16ns) (500/800MHz)	[b]CHARMM36m (CG-25ns) (500/800MHz)
R1 (s^{-1})	2.8 / 2.6	1.3 / 1.1	1.4 / 1.1	1.2 / 1.0	1.0 / 0.85	1.0 / 0.78
R2 (s-^{1})	4.9 / 5.9	4.1 / 5.0	5.4 / 6.7	13.2 / 16.7	14.9 / 18.9	19.2 / 24.3
NOE	-0.56 / 0.34	-0.05 / 0.25	0.14 / 0.38	0.03 / 0.39	0.14 / 0.45	0.33 / 0.52

References

1. N. A. Ayoub, J. E. Garb, R. M. Tinghitella, M. A. Collin, C. Y. Hayashi, Blueprint for a High-Performance Biomaterial: Full-Length Spider Dragline Silk Genes. *PLoS ONE* **2**, e514 (2007).
2. A. Irbäck, S. Mohanty, PROFASI: A Monte Carlo simulation package for protein folding and aggregation. *Journal of Computational Chemistry* **27**, 1548–1555 (2006).
3. G. Askarieh *et al.*, Self-assembly of Spider Silk Proteins is Controlled by a pH-sensitive Relay. *Nature* **465**, 236–238 (2010).
4. F. Hagn *et al.*, A conserved spider silk domain acts as a molecular switch that controls fibre assembly. *Nature* **465**, 239–242 (2010).
5. W. Lee, M. Rahimi, Y. Lee, A. Chiu, POKY: a software suite for multidimensional NMR and 3D structure calculation of biomolecules. *Bioinformatics* **37**, 3041–3042 (2021).
6. W. Lee, M. Tonelli, J. L. Markley, NMRFAM-SPARKY: enhanced software for biomolecular NMR spectroscopy. *Bioinformatics* **31**, 1325–1327 (2014).
7. F. Delaglio *et al.*, NMRPipe: A multidimensional spectral processing system based on UNIX pipes. *Journal of Biomolecular NMR* **6**, 277–293 (1995).
8. D. R. Muhandiram, L. E. Kay, Gradient-Enhanced Triple-Resonance Three-Dimensional NMR Experiments with Improved Sensitivity. *Journal of Magnetic Resonance, Series B* **103**, 203–216 (1994).
9. M. Wittekind, HNCACB, a highsensitivity 3D NMR experiment to correlate amideproton and nitrogen resonances with the α-carbon and β-carbon resonances in proteins. *J. Magn. Reson. ser. B* **101**, 214–217 (1993).
10. S. Grzesiek, A. Bax, Amino acid type determination in the sequential assignment procedure of uniformly 13C/15N-enriched proteins. *Journal of Biomolecular NMR* **3**, 185–204 (1993).
11. W. Bermel, I. C. Felli, R. Kümmerle, R. Pierattelli, 13C Direct-detection biomolecular NMR. *Concepts in Magnetic Resonance Part A* **32A**, 183–200 (2008).
12. Y. Shen, A. Bax, Protein backbone and sidechain torsion angles predicted from NMR chemical shifts using artificial neural networks. *Journal of Biomolecular NMR* **56**, 227–241 (2013).
13. Y. Shen *et al.*, Consistent blind protein structure generation from NMR chemical shift data. *Proc Natl Acad Sci U S A* **105**, 4685–4690 (2008).
14. Y. Shen, R. Vernon, D. Baker, A. Bax, De novo protein structure generation from incomplete chemical shift assignments. *Journal of Biomolecular NMR* **43**, 63–78 (2009).
15. N. Michaud-Agrawal, E. J. Denning, T. B. Woolf, O. Beckstein, MDAnalysis: A toolkit for the analysis of molecular dynamics simulations. *Journal of Computational Chemistry* **32**, 2319–2327 (2011).
16. J. Huang *et al.*, CHARMM36m: an improved force field for folded and intrinsically disordered proteins. *Nature Methods* **14**, 71–73 (2017).
17. H. Liu, D. Song, H. Lu, R. Luo, H.-F. Chen, Intrinsically disordered protein-specific force field CHARMM36IDPSFF. *Chemical Biology & Drug Design* **92**, 1722–1735 (2018).
18. W. G. Touw *et al.*, A series of PDB-related databanks for everyday needs. *Nucleic Acids Research* **43**, D364–D368 (2014).
19. P.-c. Chen, M. Hologne, O. Walker, J. Hennig, Ab Initio Prediction of NMR Spin Relaxation Parameters from Molecular Dynamics Simulations. *Journal of Chemical Theory and Computation* **14**, 1009–1019 (2018).

20. J. García de la Torre, M. L. Huertas, B. Carrasco, HYDRONMR: Prediction of NMR Relaxation of Globular Proteins from Atomic-Level Structures and Hydrodynamic Calculations. *Journal of Magnetic Resonance* **147**, 138–146 (2000).
21. P. J. Fleming, K. G. Fleming, HullRad: Fast Calculations of Folded and Disordered Protein and Nucleic Acid Hydrodynamic Properties. *Biophysical Journal* **114**, 856–869 (2018).
22. R. Fraczkiewicz, W. Braun, Exact and efficient analytical calculation of the accessible surface areas and their gradients for macromolecules. *Journal of Computational Chemistry* **19**, 319–333 (1998).
23. D. J. Wales, J. M. Carr, Quasi-Continuous Interpolation Scheme for Pathways between Distant Configurations. *J Chem Theory Comput* **8**, 5020–5034 (2012).
24. D. Xu, J. L. Yarger, G. P. Holland, Exploring the backbone dynamics of native spider silk proteins in Black Widow silk glands with solution-state NMR spectroscopy. *Polymer* **55**, 3879–3885 (2014).
